# Rapid access to polycyclic N-heteroarenes from unactivated, simple azines via a base-promoted Minisci-type annulation

**DOI:** 10.1038/s41467-022-30086-0

**Published:** 2022-05-03

**Authors:** Jae Bin Lee, Gun Ha Kim, Ji Hwan Jeon, Seo Yeong Jeong, Soochan Lee, Jaehyun Park, Doyoung Lee, Youngkook Kwon, Jeong Kon Seo, Joong-Hyun Chun, Seok Ju Kang, Wonyoung Choe, Jan-Uwe Rohde, Sung You Hong

**Affiliations:** 1 Department of Chemistry, Ulsan National Institute of Science and Technology (UNIST), 50 UNIST-gil, Ulsan, 44919 Republic of Korea; 2 School of Energy and Chemical Engineering, UNIST, 50 UNIST-gil, Ulsan, 44919 Republic of Korea; 3 UNIST Central Research Facilities (UCRF), UNIST, 50 UNIST-gil, Ulsan, 44919 Republic of Korea; 4 Department of Nuclear Medicine, Yonsei University College of Medicine, Seoul, 03722 Republic of Korea

**Keywords:** Organocatalysis, Synthetic chemistry methodology

## Abstract

Conventional synthetic methods to yield polycyclic heteroarenes have largely relied on metal-mediated arylation reactions requiring pre-functionalised substrates. However, the functionalisation of unactivated azines has been restricted because of their intrinsic low reactivity. Herein, we report a transition-metal-free, radical relay π-extension approach to produce N-doped polycyclic aromatic compounds directly from simple azines and cyclic iodonium salts. Mechanistic and electron paramagnetic resonance studies provide evidence for the in situ generation of organic electron donors, while chemical trapping and electrochemical experiments implicate an iodanyl radical intermediate serving as a formal biaryl radical equivalent. This intermediate, formed by one-electron reduction of the cyclic iodonium salt, acts as the key intermediate driving the Minisci-type arylation reaction. The synthetic utility of this radical-based annulative π-extension method is highlighted by the preparation of an N-doped heptacyclic nanographene fragment through fourfold C–H arylation.

## Abstract (web summary)

The functionalisation of unactivated azines has been restricted because of their intrinsic low reactivity. Here the authors show a transition-metal-free, radical relay π-extension approach to produce N-doped polycyclic aromatic compounds directly from simple azines and cyclic iodonium salts.

## Introduction

Polycyclic arenes have received considerable attention not only in the synthetic chemistry community but also in the chemical industry because of their controllable electronic, electrochemical, and photophysical properties^1–5^. While the preparation of such compounds, including nanographene segments, can, in principle, be achieved in a top-down manner from bulk materials or in a bottom-up approach from molecular building blocks, the bottom-up assembly is advantageous, because it allows for the precise control of molecular size and structure^6–9^. Transition-metal-catalysed cross-coupling and C–H arylation reactions have been extensively explored in the synthesis of structurally well-defined polycyclic arenes^10–18^. However, these methods require multistep procedures, including halogenation or metalation reactions, to pre-functionalize substrates. In addition, the assembled, arylated products must be converted into the fully fused polycyclic arenes by intramolecular dehydrogenative oxidation (Scholl reaction)^19^.

**Fig. 1:**
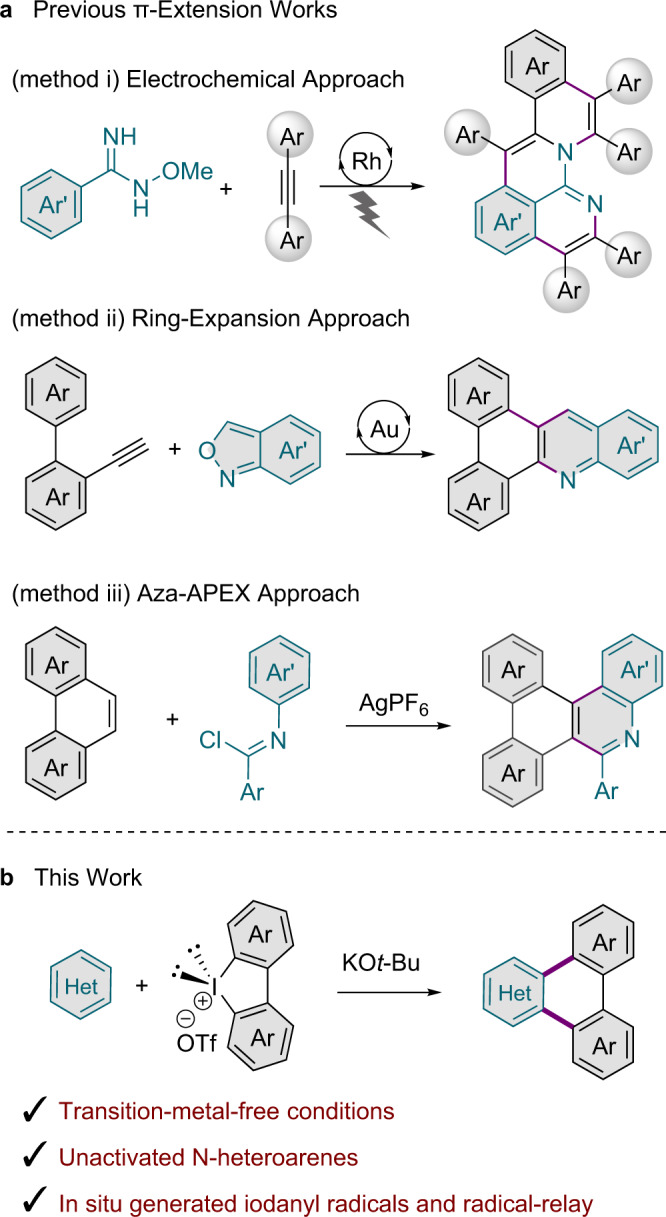
Annulative strategies to access polycyclic heteroaromatic systems. **a** Previous π-extension works. **b** This work: iodanyl-Minisci-type radical approach.

Metal-catalysed annulative π-extension (APEX) reactions are regarded as an appealing approach for rapidly constructing polycyclic arenes^20–23^, while obviating the need for pre-activation of substrates and Scholl oxidation. Recently, synthetic methods to produce N-doped polycyclic aromatic compounds (N-PACs) through π-extension chemistry have also been reported. Heteroatoms embedded within π-conjugated systems can alter their topology, band gap, and optoelectronic and electrochemical properties^24^. Ackermann and colleagues developed a Rh-electrocatalytic C–H functionalisation method (Fig. 1a, method (i)) that uses directing-group-bearing arene substrates and alkynes^25^. Hashimi et al. reported a gold-catalysed ring-expansion using *o*-ethynylbiaryls as π-extension agents (Fig. 1a, method (ii))^26^. Additionally, the Itami group described an aza-annulative approach (Fig. 1a, method (iii)) facilitating the extension of polycyclic aromatic hydrocarbons in their *K*-region positions^27^. However, these methods have still been limited to electron-rich (hetero)arene substrates and require the aid of transition-metal catalysis or silver-assisted oxidation.

Azines, six-membered-ring heteroarenes bearing nitrogen atom(s), are privileged structural moieties in biological science, pharmaceutical industry, and materials chemistry^28^. Despite significant progress, annulative transformations of unactivated, simple azines, free of pre-installed functional groups, remain a highly challenging topic because of their electron-deficient character and potential for undesired coordination^29,30^. Herein, we show the direct transformation of simple and electron-deficient heteroarenes to N-PACs based on an APEX reaction that is initiated by electron transfer and proceeds through radical relay events akin to the Minisci reaction (Fig. 1b).

## Results and discussion

### Development and optimisation

**Fig. 2:**
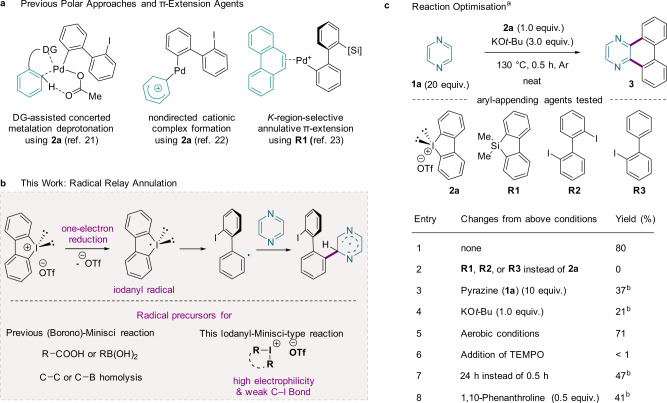
Polar *versus* radical annulation approaches. **a** Transition-metal-catalysed polar annulation approaches. **b** Transition-metal-free radical relay annulation approach, wherein an in situ generated iodanyl radical directly activates a C–H bond of an unactivated azine. **c** Reaction optimisation. ^a^Standard reaction conditions: **1a** (4.0 mmol, 20 equiv.), **2a** (0.20 mmol, 1.0 equiv.), KO*t*-Bu (0.60 mmol, 3.0 equiv.) at 130 °C under Ar for 0.5 h. Isolated yields. ^b^GC yields using *n*-dodecane as an internal standard. DG directing group; ^−^OTf triflate (CF_3_SO_3_^−^); KO*t*-Bu potassium *tert*-butoxide.

To develop a method for the synthesis of N-PACs from electron-deficient heteroarenes, we initially sought to extend existing metal-catalysed APEX reactions to the annulation of pyrazine (**1a**). In particular, we attempted to apply Itami’s cationic-palladium-catalysed *K-*region-selective APEX method, our directing-group-assisted palladium-catalysed APEX method, and an acid-promoted electrophilic APEX method (Fig. 2a; see also Supplementary Information Section 1.4)^21–23^. However, these methods failed to yield the target product **3**, which was likely due to catalyst deactivation.

Inspired by the recently developed organocatalytic methods for the construction of biaryl moieties through aryl radical species^31–40^, we then considered radical reactions as an alternative to polar reactions. In base-promoted homolytic aromatic substitution (BHAS) or unimolecular radical nucleophilic substitution (S_RN_1) reactions, aryl radicals are generated by single-electron transfer (SET) from organic electron donors (OEDs) to aryl halides. As π-extension agents, cyclic diaryliodonium salts^21,22^ and dibenzosilole derivatives^23^ have been successfully applied in both directed and nondirected C–H annulations (Fig. 2a). Given the enhanced electrophilic character as well as the weak C–I bond of hypervalent iodine(III) reagents^41–45^, we surmised that an iodanyl radical may be generated from a cyclic iodonium salt through one-electron reduction and may act as a formal biaryl radical equivalent. Coupling of a C-centred radical, formed through subsequent homolysis of the C–I bond, with an unactivated azine may then give a heteroarene radical through an iodanyl-Minisci-type radical relay reaction (Fig. 2b).

Therefore, we examined transition-metal-free screening conditions for the annulation of **1a** by varying the base, organic additive, aryl-appending partner, solvent, and reaction temperature (see also the Supplementary Information, Section 1.5). Remarkably, the use of potassium *tert*-butoxide (KO*t*-Bu) and cyclic diphenyliodonium salt **2a** at 130 °C yielded the desired dibenzo[*f*,*h*]quinoxaline (**3**) in 80% yield (entry 1 in Fig. 2c). Intriguingly, other aryl-appending agents (**R1**, **R2**, and **R3**) failed to deliver the target compound, indicating the importance of iodonium salt **2a** (entry 2). **1a** was used not only as the substrate but also as the solvent, and an excess of it (20 equiv.) was required for optimal yield (entry 3). The use of KO*t*-Bu (3.0 equiv.) was determined to be crucial, because reducing its amount or replacing it with other bases significantly diminished the product yields (entry 4; see also Supplementary Tables 1 and 2). Under aerobic conditions the APEX product **3** was obtained in a slightly reduced yield (entry 5). However, the addition of 2,2,6,6-tetramethyl-1-piperidinyloxy (TEMPO) inhibited the reaction, indicating the involvement of a radical pathway in this annulation (entry 6). A longer reaction time lowered the yield (entry 7; see also Supplementary Table 4). Organic additives, including 1,10-phenanthroline and its derivatives, did not improve the reaction efficiency (entry 8; see also Supplementary Table 6), consistent with a role of **1a** as an inherent additive that produces OEDs in the presence of KO*t*-Bu.

### Scope of the reaction

**Fig. 3:**
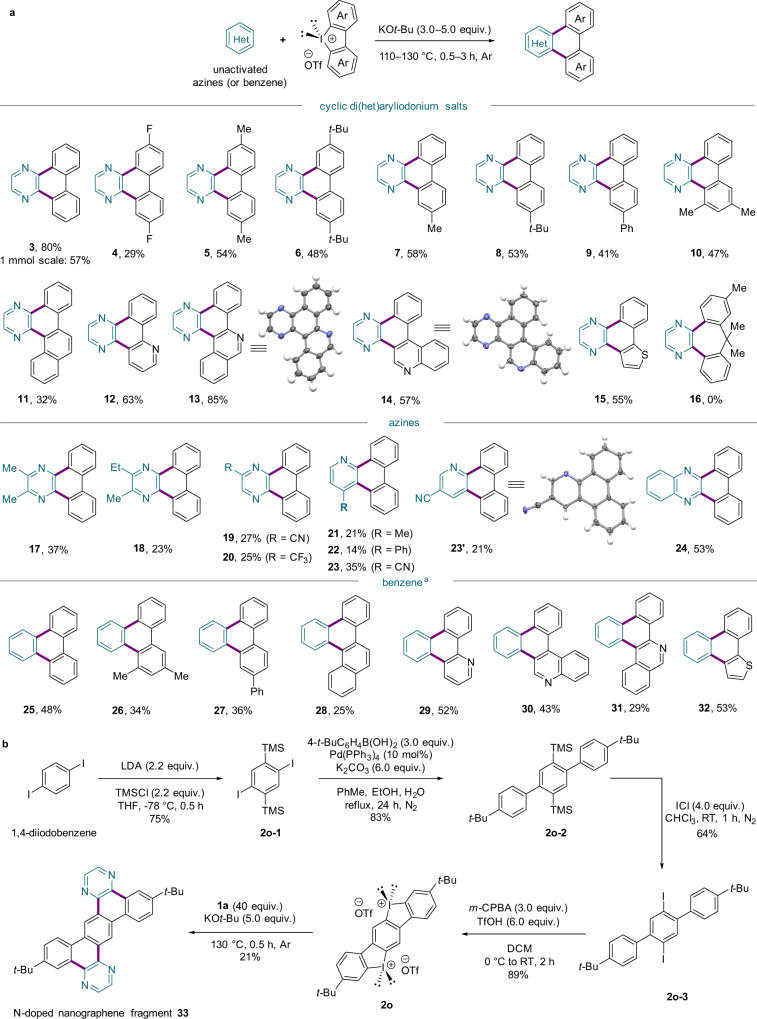
Scope of transition-metal-free APEX reactions. **a** Reactions were carried out on a 0.20 mmol scale for cyclic aryliodonium salt **2**. ^a^Conditions for reactions with benzene: **1a** (2.0 equiv., used as an additive), **2** (1.0 equiv.), KO*t*-Bu (5.0 equiv.) in benzene (2 mL) at 110 °C under Ar for 3 h. **b** Access to an N-doped nanographene fragment. Isolated yields are reported.

A series of iodonium salts (**2**) was explored using this protocol (Fig. 3a). While a fluorine-substituted aryl-transfer reagent gave **4** in a decreased yield, a broad range of alkyl- and aryl-substituted cyclic diaryliodonium salts tolerated the annulation conditions and provided the corresponding products (**5**–**11**) in moderate yields. Various electron-rich and electron-deficient heterocyclic iodonium salts were also subjected to the reaction conditions to yield the desired π-extended N-PACs (**12**–**15**). However, a six-membered cyclic iodonium salt did not operate with this protocol and failed to produce the corresponding product (**16**). Substituted azines gave products **17**–**23**' in diminished yields, and quinoxaline provided the site-selectively annulated product **24** in 53% yield. Furthermore, we also conducted additional substrate scope studies using quinoxaline (see also the Supplementary Information, Section 1.6.2). The desired products were observed, and interestingly the reactivity pattern of this radical APEX strategy involving the azine-moiety activation within the quinoxaline framework is complementary to Itami’s *M*-region APEX^46^ that operates through a two-step dearomatization-annulation approach. This annulation protocol not only is compatible with simple azines but can also be amended to unactivated benzene to produce the PACs **25**–**32**. These reactions were performed with **1a** as an additive (2 equiv.) to provide a pathway for OED formation (*vide infra*). Notably, products **30** and **31** were inaccessible through direct C–H arylation reactions^22^. APEX reactions to access well-defined PACs or N-PACs typically have low-to-moderate yields because they (i) employ unfunctionalized substrates and (ii) involve twofold C–C bond formation via C–H functionalization. Moreover, the annulative functionalization of simple azines has not been demonstrated yet. Given the difficulties in constructing a diverse array of tailored N-PACs, the reaction scope and yields in this work are comparable to those of previously reported APEX methods^22,27^.

### Access to an N-doped heptacyclic nanographene fragment

Remarkably, this protocol was readily applicable to a one-pot, fourfold arylation that yielded the N-doped heptacyclic nanographene fragment **33** in a bottom-up manner (Fig. 3b). Our synthetic route commenced with 1,4-diiodo-2,5-bis(trimethylsilyl)benzene (**2o-1**), which was prepared in 75% yield by treating trimethylsilyl chloride (TMSCl) and 1,4-diiodobenzene with lithium diisopropylamide (LDA) in THF at −78 °C^47^. Subsequent Suzuki–Miyaura cross-coupling with 4-*tert*-butylphenylboronic acid as the aryl-coupling partner was employed to afford disilylteraryl **2o-2** in 83% yield. Iodination of **2o-2** with ICl provided diiodoteraryl **2o-3**, which was converted into bis(iodonium) salt **2o** by *meta*-chloroperbenzoic acid (*m*-CPBA)-mediated oxidation. Finally, the desired N-doped nanographene fragment **33** could be obtained in 21% yield via our annulation protocol.

### Electron paramagnetic resonance (EPR) spectroscopy

**Fig. 4:**
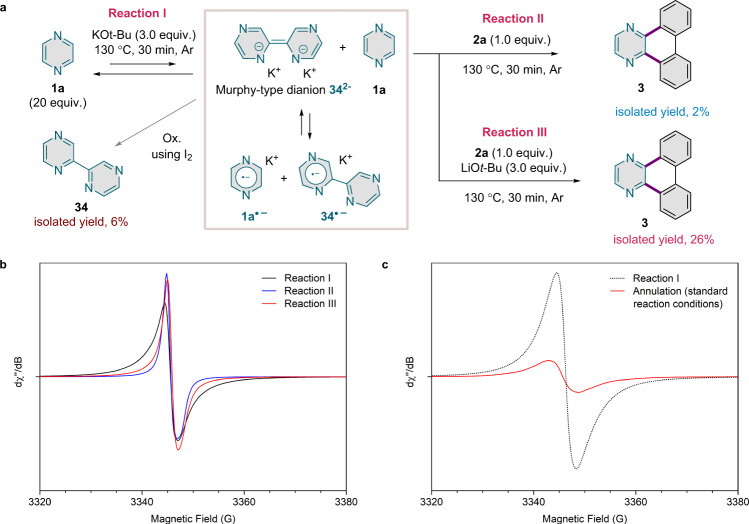
EPR spectroscopic studies. **a** Experiments illustrating the dual role of KO*t*-Bu. **b** EPR spectra (X-band, 295 K) of samples from the reaction of **1a** with KO*t*-Bu (reaction I; solid black line), from the two-step reaction of **1a** with KO*t*-Bu and then with **2a** (reaction II; solid blue line), and from the two-step reaction of **1a** with KO*t*-Bu and then with **2a** and LiO*t*-Bu (reaction III; solid red line). **c** EPR spectra (X-band, 295 K) of samples from the reaction of **1a** with KO*t*-Bu (reaction I; dotted black line) and from the annulation of **1a** with KO*t*-Bu and **2a** (solid red line), with sample transfer conducted in an Ar atmosphere in both cases. The spectrum of the annulation was acquired using a microwave power one hundred times that used for the spectrum of the **1a**–KO*t*-Bu reaction.

To gain insight into the reaction mechanism of the annulation of pyrazine, we first considered the reaction of **1a** with KO*t*-Bu (Fig. 4a, reaction I; see also Supplementary Information Sections 1.9 and 1.10). The related reactions of 1,10-phenanthroline or pyridine with KO*t*-Bu were reported to proceed through deprotonation and coupling of the N-heterocyclic compound to afford electron-rich dianions^32,48^. Additionally, in the case of 1,10-phenanthroline, radical species were independently observed by the Murphy^32^ and the Jutand/Lei^33^ groups. Indeed, EPR spectra of samples from the reaction of **1a** with KO*t*-Bu exhibit a resonance signal centred at *g* = 2.003 (Fig. 4b and Supplementary Information Section 1.11). This signal appears to be very similar to the one reported for the corresponding reaction of 1,10-phenanthroline in toluene^33^, which was attributed to a mixture of the phenanthroline and diphenanthrolinyl radical anions^32^. Based on that precedent, we suggest that **1a** first produces the dianion **34**^**2−**^ as a strong OED and then comproportionates with the dianion to give the two corresponding radical anions (**1a·**^**−**^/**34·**^**−**^) as additional OEDs. This reaction sequence is supported by the isolation of 2,2'-bipyrazine (**34**) after oxidative workup using iodine (Fig. 4a and Supplementary Information Section 1.9).

We then evaluated the possibility of product formation from the proposed dianion/radical anion species. Pre-activation of **1a** with KO*t*-Bu for 30 min followed by addition of iodonium salt **2a** gave product **3** only in a marginal yield (Fig. 4a, reaction II, and Supplementary Information Section 1.10). Presumably, KO*t*-Bu is required in the ensuing reaction but had been entirely consumed by reaction with **1a** prior to the addition of **2a**. By contrast, **3** was observed when LiO*t*-Bu was added along with **2a** in the second step (reaction III), indicating that a base is required to complete the reaction. Moreover, EPR spectra of samples from these two-step experiments show a substantial decrease of the intensity of the resonance signal (Fig. 4b and Supplementary Section 1.11, Supplementary Fig. 13), demonstrating that one or both of the radical anions are directly involved in the reaction with **2a**.

To elucidate the intermediacy of radical species in the annulation of **1a** under standard conditions, we measured EPR spectra of samples taken from this reaction. These spectra also display a resonance signal at *g* = 2.003, but this signal is of much lower intensity than that of the pyrazine-derived radical anions **1a·**^**−**^/**34·**^**−**^ (Fig. 4c). It also is broader (peak-to-peak separation, 6 G vs 4 G) and less symmetric (Supplementary Fig. 14). These differences indicate that the radical anions **1a·**^**−**^/**34·**^**−**^ do not accumulate substantially, if at all, and that other radical species are present during the annulation. Furthermore, those species are, unlike **1a·**^**−**^/**34·**^**−**^, fairly sensitive to air, as is evident from the considerably lower signal intensity and the appearance of a shoulder in spectra of samples handled in air (Supplementary Fig. 16).

### Mechanistic studies: iodanyl radical intermediacy and electron transfer

**Fig. 5:**
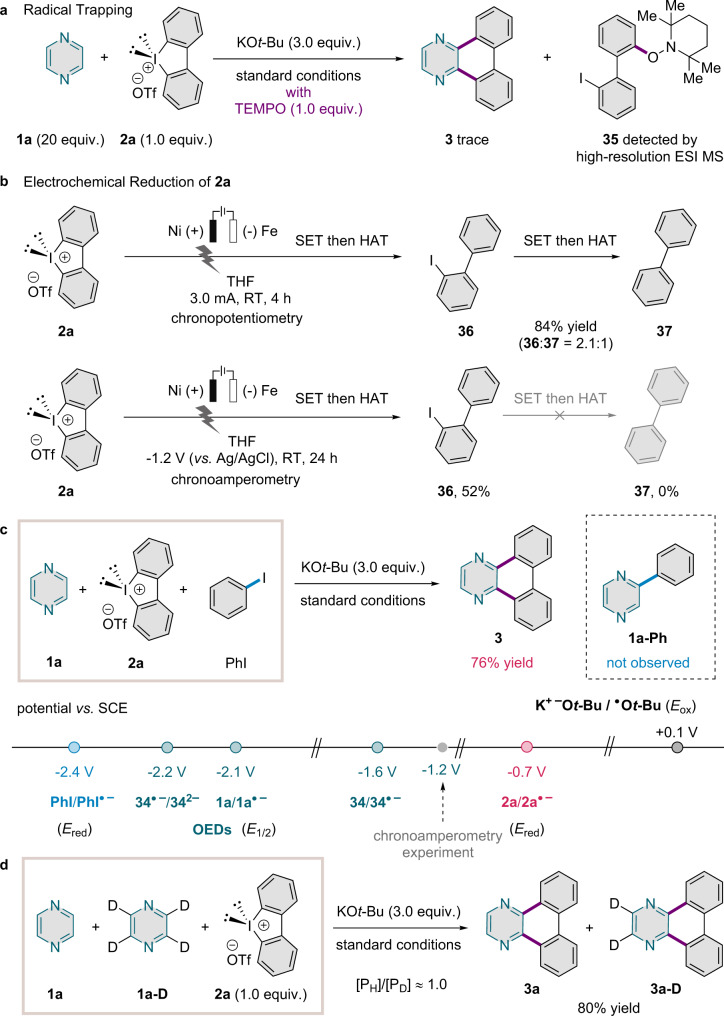
Mechanistic studies. **a** Radical trapping. **b** Electrochemical reduction of **2a**. **c** Competition experiments and CV measurements. Potentials were converted to the SCE scale using ferrocene as an external standard, where necessary. **d** KIE experiment.

The combined reaction system of KO*t*-Bu and iodonium salt allowed the otherwise challenging APEX of simple azines. In this section, we provide experimental evidence of an iodanyl radical intermediate and the involvement of an electron transfer process in the reaction mechanism. Analysis of the radical trapping experiment described above by high-resolution electrospray ionization mass spectrometry (ESI MS) revealed the formation of the TEMPO adduct of an iodobiphenyl radical (**35**) (Fig. 5a; see also Supplementary Information Section 1.12). This radical may have been derived from **2a** by one-electron reduction to give an iodanyl radical intermediate and subsequent C–I homolysis. To probe whether formation of this radical by electron transfer from a strong electron donor is feasible, we performed several electrochemical and competition experiments. The electrochemical reduction of iodonium salt **2a** (Fig. 5b) under chronopotentiometric conditions (at a constant current of 3.0 mA in THF) afforded both 2-iodobiphenyl (**36**) and biphenyl (**37**). In contrast, reduction under chronoamperometric conditions (at a constant potential of −1.2 V *versus* Ag/AgCl in THF) afforded only **36**, revealing the importance of the redox potential in the selective formation of **36**. The formation of compound **36** can be explained by hydrogen-atom transfer (HAT) to an iodobiphenyl radical. The source of the H atoms is the solvent, THF, as was confirmed by an experiment with deuterated solvent, THF-*d*_8_ (see the Supplementary Information Section 1.13).

A competition experiment of the arylation with iodonium salt **2a** and PhI produced only **3** and no **1a-Ph**, revealing that the hypervalent iodine compound was inherently more reactive (Fig. 5c). Compound **2a** also exhibits a much more favourable reduction potential (*E*_red_^2a/2a•−^ = −0.7 V *versus* SCE in DMF) than PhI (*E*_red_^PhI/PhI•−^ = −2.4 V *versus* SCE in DMF)^32^. For comparison, the reduction potential of Ph_2_IBF_4_ in MeCN was reported as −0.58 V (*versus* Ag/AgCl)^49^. The reduction potential of 2-iodobiphenyl is −2.2 V (*versus* SCE in DMF), which is significantly more negative than the potential applied in the chronoamperometric experiment. Relevant redox potentials of the OEDs presumably present (i.e., **34**^2−^, **34·**^**−**^, and **1a·**^−^) were determined from cyclic voltammograms of **1a** and **34**. Unlike the mismatched potential (*E*_ox_) of KO*t*-Bu^32,33^, these redox potentials are located in the matched potential range of −1.6–−2.2 V toward iodonium salt **2a** (Fig. 5c; see also Supplementary Information Section 1.15).

The H/D kinetic isotope effect (KIE) for this annulation reaction was also investigated, using **1a** and its fully deuterated isotopologue (**1a-D**), to examine whether deprotonation reactions are involved in the rate-determining step (RDS) (Fig. 5d; see Supplementary Information Section 1.16.2). In a competition experiment, with the iodonium salt as the limiting reactant, a KIE value of [P_H_]/[P_D_] ≈ 1.0 was observed, indicating that the RDS was not associated with deprotonation of C–H bond by KO*t*-Bu. This result is in the line with the previous results from the Shirakawa/Hayashi group on the metal-free synthesis of biaryl compounds through radical chemistry^39^. As suggested by Murphy and Shirakawa/Hayashi for the relevant BHAS reactions of arenes, OED formation^50^ or one-electron reduction^39^ might be related to the RDS in this radical relay annulation.

### Mechanistic discussion

BHAS has been applied as a transition-metal-free method to furnish biphenyl compounds^39,40^. The combination of alkali metal *tert*-butoxides, commonly KO*t*-Bu and NaO*t*-Bu, with organic additives plays a critical role in the initiation of the radical relay by converting aryl halides into aryl radicals, which have emerged as versatile synthetic intermediates in the construction of C–C bonds. In this work, we questioned whether cyclic diaryliodonium salts can act as radical precursors in annulation reactions. The working hypothesis was validated by a combined mechanistic investigation: Organic radical species attributable to OED formation were detected by EPR spectroscopy and shown to be consumed in the reaction with cyclic diphenyliodonium salt **2a**, forming the annulation product **3** (Fig. 4). The involvement of radical intermediates in the annulation reaction was confirmed by a trapping experiment, which completely shut down the reaction and gave rise to the TEMPO-iodobiphenyl adduct (Fig. 2c, entry 6, and Fig. 5a). An iodobiphenyl radical intermediate was also implicated in the electrochemical reduction of **2a** (Fig. 5b), and cyclic voltammetry demonstrated matched redox potentials for the reduction of cyclic diphenyliodonium salt **2a** by the OEDs identified in our mechanistic and spectroscopic studies (**34**^2−^, **34**^•−^, and **1a·**^**−**^; Fig. 5c).

**Fig. 6:**
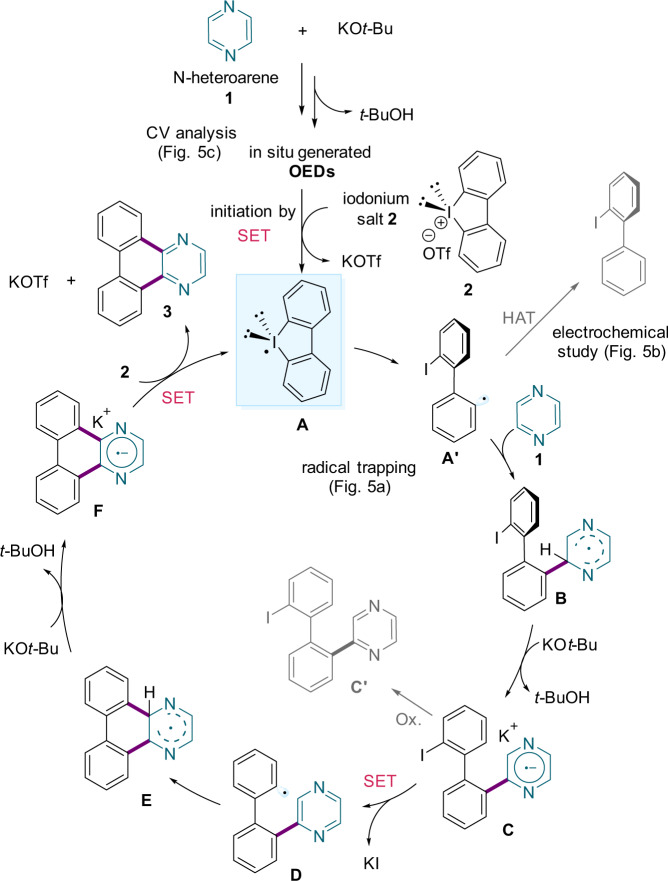
Proposed iodanyl-Minisci-type APEX reaction mechanism of simple azines. This radical relay-based annulation approach allows the formation of polycyclic N-heteroarenes.

On the basis of our experimental studies and literature reports^32–40,51–55^, we propose that the mechanism of this annulation proceeds via a base-promoted cascade of two radical relay events (Fig. 6). The reaction pathway is initiated through the one-electron reduction of electrophilic diaryliodonium salt **2** by the OEDs, formed in situ from **1** and KO*t-*Bu, to generate reactive iodanyl radical **A**^45,56^. Homolysis of the C–I bond affords the carbon-centred radical **A'**, which undergoes an iodanyl-Minisci-type addition to heteroarene **1** to afford aryl-heteroaryl radical species **B**. Subsequent deprotonation by KO*t*-Bu produces monoarylated radical anion **C**, whose oxidation product (**C'**) was detected by high-resolution ESI MS (see also the Supplementary Information Section 1.17). Intramolecular SET generates carbon-centred radical **D** upon release of KI, which is followed by radical addition and deprotonation of intermediate **E** by KO*t*-Bu to provide radical anion **F**. Finally, SET from **F** to iodonium salt **2** regenerates iodanyl radical **A**, while furnishing the desired annulation product **3**. A table summarizing the mechanistic and spectroscopic studies is also shown in the Supplementary Information (Section 1.19).

We have demonstrated a Minisci-type annulation of simple azines to rapidly access polycyclic N-heteroarenes. Mechanistic and spectroscopic studies provide experimental evidence of one-electron reduction of a cyclic iodonium salt by in situ generated organic electron donors affording an iodanyl radical, which may trigger a radical relay cascade to complete the annulative aryl-appending process. The synthetic utility of this iodanyl-Minisci-type reaction is highlighted by the preparation of an N-doped heptacyclic nanographene fragment. We anticipate that our transition-metal-free, radical-based protocol and mechanistic studies can contribute to the straightforward construction of complex polycyclic heteroaromatic compounds.

## Methods

### General procedure for APEX of azines with cyclic iodonium salts

In an Ar-filled glove box, a screw-cap 1-dram vial was charged with cyclic diaryliodonium salt **2** (0.20 mmol, 1.0 equiv.), KO*t*-Bu (0.60 mmol, 3.0 equiv.) and azine **1** (4.0 mmol, 20 equiv.). After the reaction mixture was stirred at 130 °C for 30 min, it was allowed to cool to RT and diluted with DCM (2 mL). After the sample was sonicated for 1 h at RT, it was directly purified by column chromatography to afford the desired APEX product. See Supplementary Information for experimental details and the characterisation of each compound.

## Supplementary information

Supplementary Information

## Data Availability

The data that support the findings of this study are included in this published article and its Supplementary Information files. The crystallographic data generated in this study have been deposited at the Cambridge Crystallographic Data Centre (CCDC) under deposition numbers 2047992 (**13**), 2047991 (**14**), and 2116428 (**23'**). These crystallographic data can be obtained free of charge from the Cambridge Crystallographic Data Centre via http://www.ccdc.cam.ac.uk/data_request/cif.
