# Supplementary material for: Rapid access to polycyclic N-heteroarenes from unactivated, simple azines via a base-promoted Minisci-type annulation
Source: Nat Commun. 2022 May 3;13:2421. doi: 10.1038/s41467-022-30086-0 (PMC9065069; doi:10.1038/s41467-022-30086-0)
Supplement: Supplementary file 1 — Supplementary Information [file 41467_2022_30086_MOESM1_ESM.pdf]

Supplementary Information

Rapid access to polycyclic N-heteroarenes from unactivated, simple azines via a base-promoted Minisci-type annulation

Jae Bin Lee, Gun Ha Kim, Ji Hwan Jeon, Seo Yeong Jeong, Soochan Lee, Jaehyun Park, Doyoung Lee, Youngkook Kwon, Jeong Kon Seo, Joong-Hyun Chun, Seok Ju Kang, Wonyoung Choe, Jan-Uwe Rohde* and Sung You Hong*

*Corresponding authors

E-mails: rohde@unist.ac.kr, syhong@unist.ac.kr

Table of Contents

1. Supplementary Methods ---------- 3

1.1. General Information ---------- 3

1.2. Materials and Methods ---------- 3

1.3. Preparation of Cyclic Diaryliodonium Salts 2 ---------- 4

1.4. Investigation of Previously Reported APEX Protocols for N-PAC 3 ---------- 8

1.5. Reaction Optimisation ---------- 10

1.6. Synthesis of Azatriphenylene and Its Analogues ---------- 16

1.7. Synthesis of Triphenylene and Its Analogues ---------- 30

1.8. Access to an N-Doped Heptacyclic Nanographene Fragment (33) ---------- 34

1.9. Formation of 2,2'-Bipyrazine (34) ---------- 35

1.10. Two-Step Annulation Experiments ---------- 37

1.11. EPR Spectroscopic Analysis ---------- 39

1.12. Chemical Inhibition Experiment with TEMPO ---------- 45

1.13. Electrochemical Reduction ---------- 46

1.14. Competition Experiment Between 2a and PhI ---------- 50

1.15. Cyclic Voltammetry Studies ---------- 51

1.16. Deuterium-Labelling Experiments ---------- 55

1.17. Evidence of a Reaction Intermediate ---------- 58

1.18. X-Ray Crystallographic Data of 13, 14, and 23' ---------- 59

1.19. Summary of the Mechanistic Studies ---------- 62

1.20. NMR Spectra ---------- 63

2. Supplementary References ---------- 97

1. Supplementary Methods

1.1. General Information

Proton, carbon, and fluorine nuclear magnetic resonance spectra were taken on an Agilent 400-MR DD2 (^{1}H NMR: 400 MHz; ^{13}C NMR: 100 MHz; ^{19}F NMR: 376 Hz) or on an Agilent/Varian 600 spectrometer (^{1}H NMR: 600 MHz; ^{13}C NMR: 150 MHz). Chemical shifts (δ) are quoted in ppm, and referenced to residual solvent resonances (^{1}H NMR: $CHCl_3$ at 7.26 ppm, DMSO at 2.50 ppm, C_2HDCl_4 at 6.00 ppm and ^{13}C NMR: $CDCl_3$ at 77.16 ppm, DMSO-d_6 at 39.52 ppm, $C_2D_2Cl_4$ at 73.80 ppm). α,α,α-Trifluorotoluene ($CDCl_3$ at -62.61, and DMSO-d_6 at -60.94)[1] as the external standard were used for ^{19}F NMR spectra. Data for ^{1}H NMR are reported as follows: δ (ppm), multiplicity (s = singlet; d = doublet; t = triplet; q = quartet; m = multiplet), coupling constants *J* (given in Hz), and then integration.

Thin layer chromatography (TLC) was performed on Merck 250 μm thick silica gel 60 F_{254} plates. Visualisation of the plate was achieved using an ultraviolet lamp (λ_{max}, 254 nm). Purification was done by column chromatography using Merck silica gel 60 (0.040-0.063 mm).

GC analysis was performed on an Agilent Technologies GCMS 7890A equipped with a BR-ms column (30 m x 0.25 mm x 0.25 μm, pressure = 20.0 kPa, detector = EI, 300 °C) with nitrogen gas as carrier.

High resolution mass spectrometry (HRMS) spectra were determined on a Q Exactive™ Plus Hybrid Quadrupole-Orbitrap™ mass spectrometer from Thermo Scientific.

Cyclic voltammetry was conducted according to the previously reported literature[2]. Electrochemical evaluation of cyclic diphenyliodonium salt was conducted by using a multichannel potentiostat (VMP3 Multichannel Workstation, BioLogic) and a three-electrode beaker cell.

Electrolysis was performed in a standard two-electrode and three-electrode vial cell controlled by a potentiostat (SP-300, NeoScience). Fe metal and Ni foam (1.0 cm x 1.2 cm; area = 1.2 cm^2) were connected to copper wire, in which the upper part of copper wire was separated with rubber bracket to avoid the short of the electrode system.

1.2. Materials and Methods

Unless otherwise noted, all commercial reagents were purchased from standard suppliers (Sigma-Aldrich, Alfa Aesar, or TCI) and stored in an Ar-filled glovebox. Potassium *tert*-butoxide was purchased from Sigma-Aldrich and was used as received.

1.3. Preparation of Cyclic Diaryliodonium Salts 2

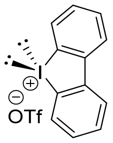

2a

Compound **2a** was prepared according to a previously reported procedure. Spectral data matched the literature values[3].

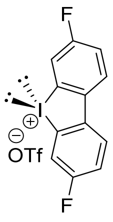

2b

Compound **2b** was prepared according to a previously reported procedure. Spectral data matched the literature values[3].

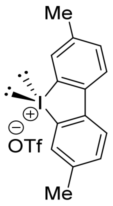

2c

Compound **2c** was prepared according to a previously reported procedure. Spectral data matched the literature values[3].

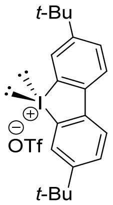

2d

Compound **2d** was prepared according to a previously reported procedure. Spectral data matched the literature values[3].

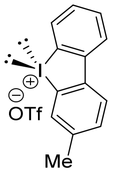

2e

Compound **2e** was prepared according to a previously reported procedure. Spectral data matched the literature values[3].

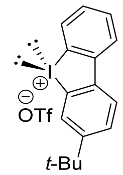

2f

Compound **2f** was prepared according to a previously reported procedure. Spectral data matched the literature values[3].

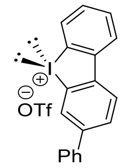

2g

Compound **2g** was prepared according to a previously reported procedure. Spectral data matched the literature values[3].

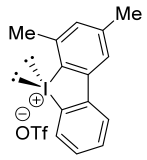

2h

Compound **2h** was prepared according to a previously reported procedure. Spectral data matched the literature values[4].

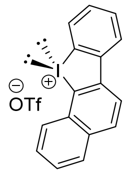

2i

Compound **2i** was prepared according to a previously reported procedure. Spectral data matched the literature values[5].

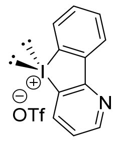

2j

Compound **2j** was prepared according to a previously reported procedure. Spectral data matched the literature values[6].

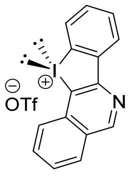

2k

Compound **2k** was prepared according to a previously reported procedure. Spectral data matched the literature values[7].

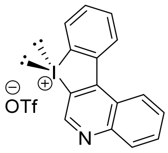

2l

Compound **2l** was prepared according to a previously reported procedure. Spectral data matched the literature values[7].

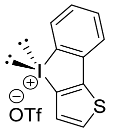

2m

Compound **2m** was prepared according to a previously reported procedure. Spectral data matched the literature values[5].

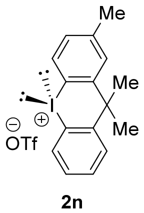

2n

Compound **2n** was prepared according to a previously reported procedure. Spectral data matched the literature values[8].

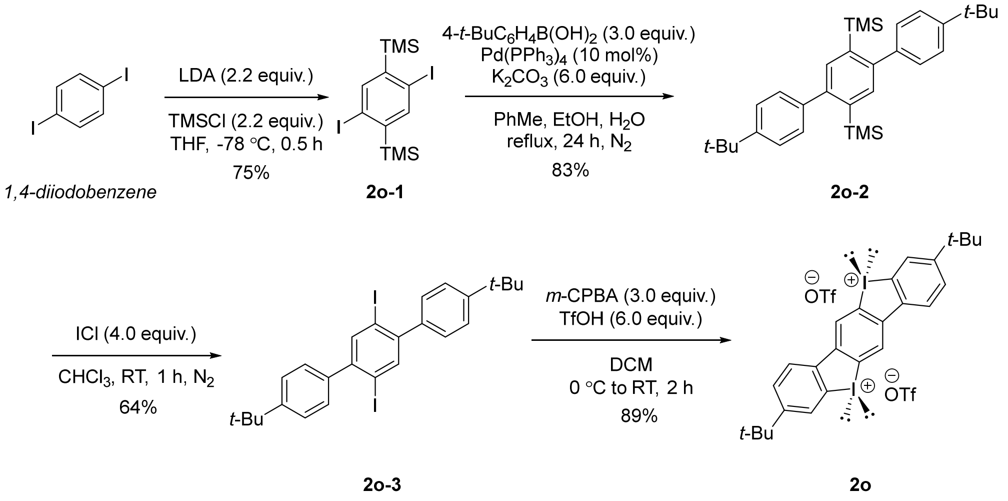

Supplementary Figure 1 Reaction Synthesis of compound **2o**

2o-1 was prepared according to a previously reported procedure[9].

A flame dried round bottom flask (100 mL) was charged with 4-*tert*-butylphenyl boronic acid (9.0 mmol, 3.0 equiv.), K_2CO_3 (18.0 mmol, 6.0 equiv.), $Pd(PPh_3)_4$ (0.3 mmol, 0.1 equiv.), **2o-1** (3.0 mmol, 1.0 equiv.), PhMe (30 mL), EtOH (10 mL), and H_2O (10 mL). After the reaction mixture was refluxed (100 °C) for 24 h under a nitrogen atmosphere, the reaction mixture was extracted with EtOAc. The organic layer was dried over Na_2SO_4, filtered, concentrated under reduced pressure, and purified by column chromatography to afford compound **2o-2** (1.21 g, 83%) as a white solid.

After bubbling nitrogen through the solution of **2o-2** (2.67 mmol, 1.0 equiv.) in $CHCl_3$ (120 mL) for 20 min, an ICl solution (1 M in DCM, 10.7 mmol, 4.0 equiv.) was added in a dropwise manner. After the reaction mixture was stirred for 1 h, the reaction was quenched with aq. $Na_2S_2O_3$ solution and extracted with DCM. The organic layer was dried over Na_2SO_4, filtered, concentrated under reduced pressure, and purified by column chromatography to afford compound **2o-3** (1.02 g, 64%) as a white solid.

m-CPBA (70 wt%, 2.53 mmol, 3.0 equiv.) and TfOH (5.05 mmol, 6.0 equiv.) were added to a solution of **2o-3** (0.84 mmol, 1.0 equiv.) in anhydrous DCM (30 mL) at 0 °C. After the reaction mixture was stirred for 2 h at RT, the volatile solvent was evaporated and Et_2O (100 mL) was added. The precipitate was collected and washed with Et_2O several times to afford compound **2o** (0.70 g, 89%) as a brown solid.

^{1}H NMR (400 MHz, DMSO-*d$_6$*) δ 8.96 (s, 2H), 8.30 (d, *J* = 1.8 Hz, 2H), 8.24 (d, *J* = 8.5 Hz, 2H), 8.04 (dd, *J* = 8.4, 1.8 Hz, 2H), 1.42 (s, 18H); **^{13}C NMR** (100 MHz, DMSO-*d$_6$*) δ 156.10, 143.05, 137.62, 129.45, 128.09, 127.45, 127.00, 125.07, 123.57, 36.16, 31.29; **^{19}F NMR** (376 MHz, DMSO-*d$_6$*) δ -77.38; **ESI HRMS**: m/z $[M-2OTf]^{2+}$ calcd for $C_{26}H_{26}I_2$: 296.0057; found: 296.0057.

1.4. Investigation of Previously Reported APEX Protocols for N-PAC 3

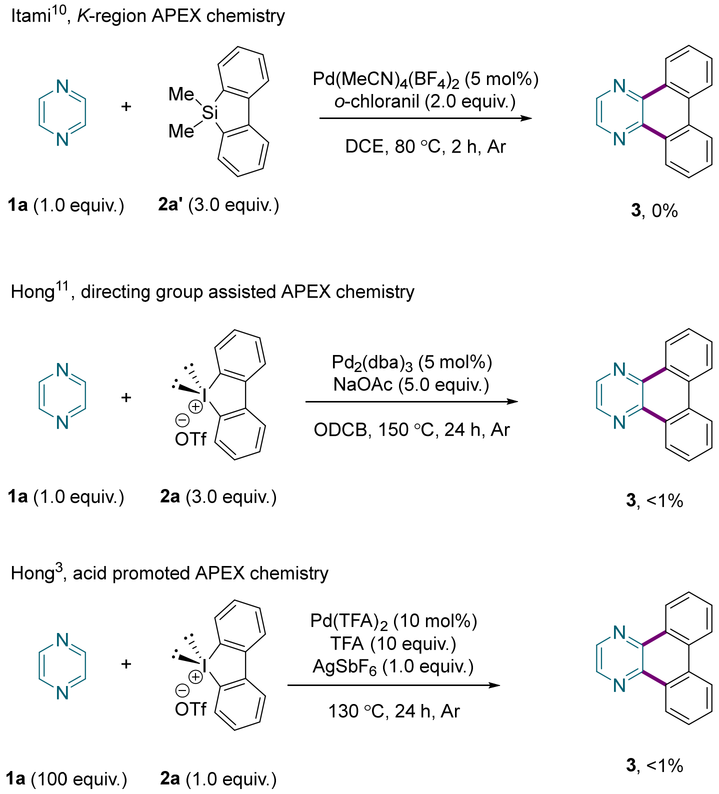

Supplementary Figure 2 Pd-catalysed APEX reactions

We initially investigated the annulation of pyrazine (**1a**) by employing a previously developed metal-catalysed annulation reactions; (i) Itami's cationic palladium catalysed *K*-region selective APEX, (ii) directing-group-assisted palladium-catalysed APEX, and (iii) acid-promoted electrophilic APEX methods. However, they did not successfully yield the target product **3**.

Detailed reaction procedure of *K*-region APEX[10]

In an Ar-filled glove box, a screw-cap 1-dram vial was charged with **1a** (0.20 mmol, 1.0 equiv.), 9,9-dimethyl-9*H*-9-silafluorene (**2a′**) (0.60 mmol, 3.0 equiv.), $Pd(MeCN)_4(BF_4)_2$ (0.01 mmol, 0.05 equiv.), *o*-chloranil (0.40 mmol, 2.0 equiv.) and DCE (2 mL). After the reaction mixture was stirred at 80 °C for 2 h, the mixture was diluted with DCM (2 mL) and checked by TLC analysis to detect the presence of compound **3**.

Detailed reaction procedure of directing group assisted APEX[11]

In an Ar-filled glove box, a screw-cap 1-dram vial was charged with **1a** (0.20 mmol, 1.0 equiv.), cyclic diphenyliodonium salt **2a** (0.60 mmol, 3.0 equiv.), $Pd_2(dba)_3$ (0.01 mmol, 0.05 equiv.), NaOAc (1.0 mmol, 5.0 equiv.) and ODCB (2 mL). After the reaction mixture was stirred at 150 °C for 24 h, the mixture was diluted with DCM (2 mL) and checked by TLC analysis to detect the presence of compound **3**.

Detailed reaction procedure of acid promoted APEX[3]

In an Ar-filled glove box, a screw-cap 1-dram vial was charged with **1a** (20 mmol, 100 equiv.), cyclic diphenyliodonium salt **2a** (0.20 mmol, 1.0 equiv.), $Pd(TFA)_2$ (0.02 mmol, 0.1 equiv.), TFA (2.0 mmol, 10 equiv.), and $AgSbF_6$ (0.20 mmol, 1.0 equiv.). After the reaction mixture was stirred at 130 °C for 24 h, the mixture was diluted with DCM (2 mL) and checked by TLC analysis to detect the presence of compound **3**.

1.5. Reaction Optimisation

Supplementary Table 1 Reaction optimisation[a]

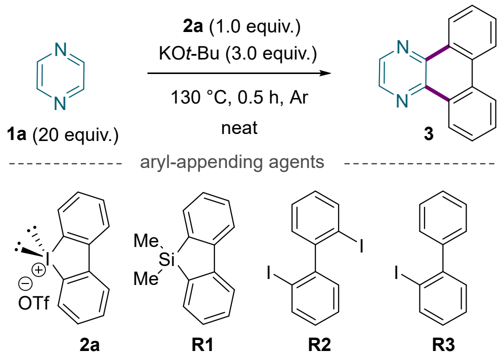

Entry	Deviations from standard conditions	Yield (%)[b]
1	None	80
2	**R1** instead of **2a**	0
3	**R2** instead of **2a**	0
4	**R3** instead of **2a**	0
5	LiO*t*-Bu instead of KO*t*-Bu	7
6	NaO*t*-Bu instead of KO*t*-Bu	8
7	Sr(O*i*-Pr)$_2$ instead of KO*t*-Bu	5
8	LiHMDS instead of KO*t*-Bu	37
9	Without KO*t*-Bu	0
10	Aerobic conditions	71

[a]Standard conditions: **1a** (4.0 mmol, 20 equiv.), **2a** (0.20 mmol, 1.0 equiv.), KO*t*-Bu (0.60 mmol, 3.0 equiv.), 130 ℃, 0.5 h under Ar. [b]Isolated yield.

Supplementary Table 2 Optimisation of KO*t*-Bu equivalents[a]

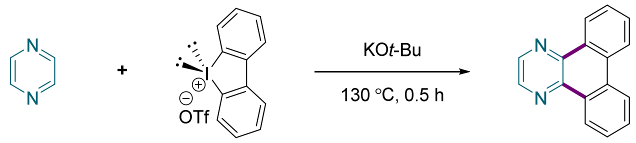

1a **2a** **3**

Entry	Equiv. of KO*t*-Bu	Yield (%)[b]
1	0.5	14
2	1.0	21
3	1.5	25
4	2.0	27
5	2.5	37
6	3.0	68
7	3.5	59
8	4.0	56
9	4.5	57
10	5.0	48

[a]Standard conditions: **1a** (4.0 mmol, 20 equiv.), **2a** (0.20 mmol, 1.0 equiv.), KO*t*-Bu, 130 °C, 0.5 h. [b]GC yields using *n*-dodecane as an internal standard.

Supplementary Table 3 Optimisation of pyrazine equivalents[a]

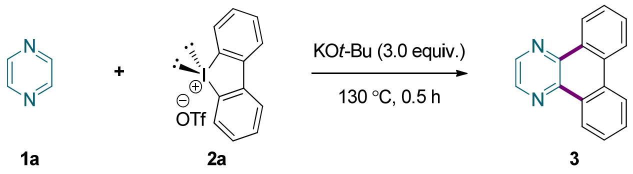

Entry	Equiv. of pyrazine	Yield (%)[b]
1	1.0	-
2	5.0	20
3	10.0	37
4	20.0	68
5	30.0	57
6	40.0	63
7	50.0	59

[a]Reaction conditions: **1a**, **2a** (0.20 mmol, 1.0 equiv.), KO*t*-Bu (0.60 mmol, 3.0 equiv.), 130 °C, 0.5 h. [b]GC yields using *n*-dodecane as an internal standard.

Supplementary Table 4 Optimisation of reaction time[a]

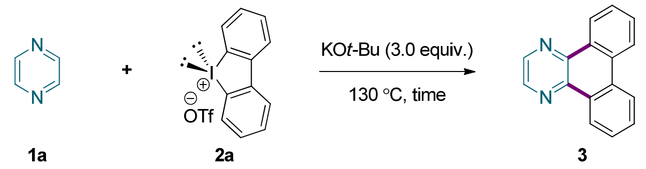

Entry	Time	Yield (%)[b]
1	5 min	35
2	10 min	42
3	30 min	68
4	1 h	56
5	2 h	54
6	4 h	40
7	6 h	36
8	24 h	47

[a]Reaction conditions: **1a** (4.0 mmol, 20 equiv.), **2a** (0.20 mmol, 1.0 equiv.), KO*t*-Bu (0.60 mmol, 3.0 equiv.), 130 °C, time. [b]GC yields using *n*-dodecane as an internal standard.

Supplementary Table 5 Optimisation of reaction temperature[a]

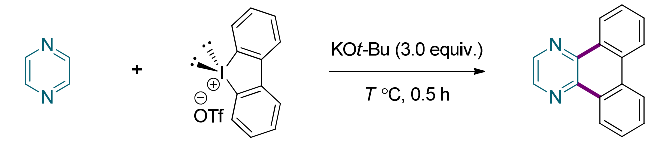

1a 2a 3

Entry	Temperature	Yield (%)[b]
1	80	56
2	100	54
3	110	62
4	120	64
5	130	68
6	140	43

[a]Reaction conditions: **1a** (4.0 mmol, 20 equiv.), **2a** (0.20 mmol, 1.0 equiv.), KO*t*-Bu (0.60 mmol, 3.0 equiv.), *T* °C, 0.5 h. [b]GC yields using *n*-dodecane as an internal standard.

Supplementary Table 6 Effect of additives[a]

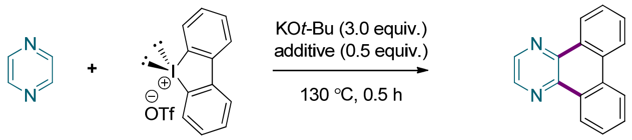

1a **2a** **3**

selected additives

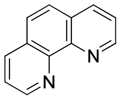

A1

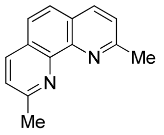

A2

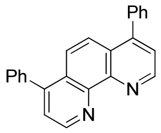

A3

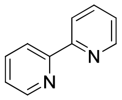

A4

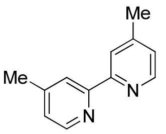

A5

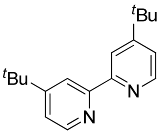

A6

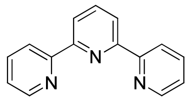

A7

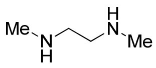

A8

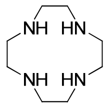

A9

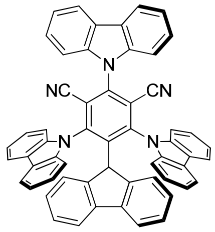

A10

Entry	Additives	Yield (%)[b]
1	None	68
2	**A1**	41
3	**A2**	50
4	**A3**	51
5	**A4**	48
6	**A5**	47
7	**A6**	58
8	**A7**	34
9	**A8**	38
10	**A9**	23
11	**A10**	23

[a]Reaction conditions: **1a** (4.0 mmol, 20 equiv.), **2a** (0.20 mmol, 1.0 equiv.), additive (0.10 mmol, 0.5 equiv.), KO*t*-Bu (0.60 mmol, 3.0 equiv.), 130 °C, 0.5 h. [b]GC yields using *n*-dodecane as an internal standard.

1.6. Synthesis of Azatriphenylene and Its Analogues

1.6.1. Substrate Scope

Dibenzo[*f*,*h*]quinoxaline (3)

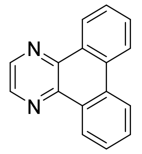

3

In an Ar-filled glove box, a screw-cap 1-dram vial was charged with cyclic diaryliodonium salt **2a** (0.20 mmol, 1.0 equiv.), KO*t*-Bu (0.60 mmol, 3.0 equiv.) and pyrazine (4.0 mmol, 20 equiv.). After the reaction mixture was stirred at 130 °C for 30 min, it was allowed to cool to RT and diluted with DCM (2 mL). After sonicating the sample for 1 h at RT, the reaction mixture was directly purified by column chromatography to afford compound **3**.

Yellow solid (37 mg, 80%); TLC R_f = 0.5 (*n*-hexane:Et_2O = 20:1); **^{1}H NMR** (400 MHz, $CDCl_3$) δ 9.24 (dd, *J* = 8.0, 1.3 Hz, 2H), 8.90 (s, 2H), 8.63 (dd, *J* = 8.0, 1.3 Hz, 2H), 7.77 (m, 4H).

The NMR spectrum of compound **3** matched previously reported literature data[12].

6,11-Difluorodibenzo[*f*,*h*]quinoxaline (4)

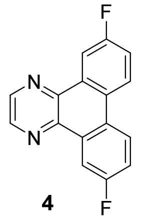

4

In an Ar-filled glove box, a screw-cap 1-dram vial was charged with cyclic diaryliodonium salt **2b** (0.20 mmol, 1.0 equiv.), KO*t*-Bu (0.60 mmol, 3.0 equiv.) and pyrazine (4.0 mmol, 20 equiv.). After the reaction mixture was stirred at 130 °C for 30 min, it was allowed to cool to RT and diluted with DCM (2 mL). After sonicating the sample for 1 h at RT, the reaction mixture was directly purified by column chromatography to afford compound **4**.

Yellow solid (15 mg, 29%); TLC R_f = 0.3 (*n*-hexane:Et_2O = 10:1); **^{1}H NMR** (400 MHz, $CDCl_3$) δ 8.93 (s, 2H), 8.84 (dd, *J* = 10.1, 2.9 Hz, 2H), 8.53 (dd, *J* = 9.0, 5.0 Hz, 2H), 7.52 (m, 2H); **^{13}C NMR** (100 MHz, $CDCl_3$) δ 163.26, 160.80, 143.96, 140.92, 131.38, 131.29, 127.30, 124.84, 124.75, 118.13, 117.89, 110.77, 110.54; **^{19}F NMR** (376 MHz, $CDCl_3$) δ -112.69; **ESI HRMS**: m/z $[M+H]^+$ calcd for $C_{16}H_9F_2N_2$: 267.0728; found: 267.0729.

6,11-Dimethyldibenzo[*f,h*]quinoxaline (5)

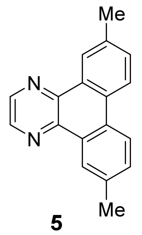

5

In an Ar-filled glove box, a screw-cap 1-dram vial was charged with cyclic diaryliodonium salt **2c** (20 mmol, 1.0 equiv.), KO*t*-Bu (0.60 mmol, 3.0 equiv.) and pyrazine (4.0 mmol, 20 equiv.). After the reaction mixture was stirred at 130 °C for 30 min, it was allowed to cool to RT and diluted with DCM (2 mL). After sonicating the sample for 1 h at RT, the reaction mixture was directly purified by column chromatography to afford compound **5**.

White solid (28 mg, 54%); TLC $R_f = 0.3$ (*n*-hexane:Et_2O = 10:1); ^{1}**H NMR** (400 MHz, $CDCl_3$) δ 8.97 (d, $J = 1.8$ Hz, 2H), 8.87 (s, 2H), 8.45 (d, $J = 8.3$ Hz, 2H), 7.58 (dd, $J = 8.3$, 1.9 Hz, 2H), 2.62 (s, 6H); ^{13}**C NMR** (100 MHz, $CDCl_3$) δ 143.00, 141.41, 137.05, 130.86, 129.17, 129.09, 124.87, 122.34, 21.45; **ESI HRMS**: m/z $[M+H]^+$ calcd for $C_{18}H_{15}N_2$: 259.1230; found: 259.1229.

6,11-Di-*tert*-butyldibenzo[*f,h*]quinoxaline (6)

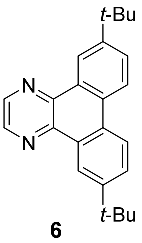

6

In an Ar-filled glove box, a screw-cap 1-dram vial was charged with cyclic diaryliodonium salt **2d** (0.20 mmol, 1.0 equiv.), KO*t*-Bu (0.60 mmol, 3.0 equiv.) and pyrazine (4.0 mmol, 20 equiv.). After the reaction mixture was stirred at 130 °C for 30 min, it was allowed to cool to RT and diluted with DCM (2 mL). After sonicating the sample for 1 h at RT, the reaction mixture was directly purified by column chromatography to afford compound **6**.

White solid (33 mg, 48%); TLC $R_f = 0.3$ (*n*-hexane:Et_2O = 10:1); ^{1}**H NMR** (400 MHz, $CDCl_3$) δ 9.23 (d, $J = 2.1$ Hz, 2H), 8.90 (s, 2H), 8.54 (d, $J = 8.6$ Hz, 2H), 7.85 (dd, $J = 8.6$, 2.2 Hz, 2H), 1.52 (s, 18H); ^{13}**C NMR** (100 MHz, $CDCl_3$) δ 150.85, 143.55, 142.39, 129.71, 129.66, 128.01, 122.96, 121.69, 35.62, 31.94; **ESI HRMS**: m/z $[M+H]^+$ calcd for $C_{24}H_{27}N_2$: 343.2169; found: 343.2167.

6-Methyldibenzo[*f,h*]quinoxaline (7)

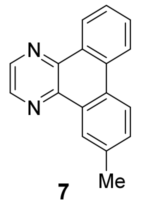

7

In an Ar-filled glove box, a screw-cap 1-dram vial was charged with cyclic diaryliodonium salt **2e** (0.20 mmol, 1.0 equiv.), KO*t*-Bu (0.60 mmol, 3.0 equiv.) and pyrazine (4.0 mmol, 20 equiv.). After the reaction mixture was stirred at 130 °C for 30 min, it was allowed to cool to RT and diluted with DCM (2 mL). After sonicating the sample for 1 h at RT, the reaction mixture was directly purified by column chromatography to afford compound **7**.

Yellow solid (28 mg, 58%); TLC R_f = 0.3 (*n*-hexane:Et_2O = 8:1); **^{1}H NMR** (400 MHz, $CDCl_3$) δ 9.20 (m, 1H), 9.02 – 8.96 (m, 1H), 8.88 (m, 2H), 8.62 – 8.56 (m, 1H), 8.52 (d, *J* = 8.4 Hz, 1H), 7.75 (m, 2H), 7.62 (m, 1H), 2.64 (s, 3H); **^{13}C NMR** (100 MHz, $CDCl_3$) δ 143.85, 143.79, 142.12, 141.88, 138.21, 132.02, 131.56, 130.03, 129.94, 129.63, 127.74, 125.81, 125.57, 123.21, 123.01, 22.12; **ESI HRMS**: m/z $[M+H]^+$ calcd for $C_{17}H_{13}N_2$: 245.1073; found: 245.1072.

6-(*tert*-Butyl)dibenzo[*f,h*]quinoxaline (8)

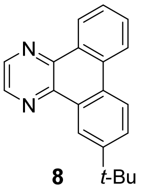

8

In an Ar-filled glove box, a screw-cap 1-dram vial was charged with cyclic diaryliodonium salt **2f** (0.20 mmol, 1.0 equiv.), KO*t*-Bu (0.60 mmol, 3.0 equiv.) and pyrazine (4.0 mmol, 20 equiv.). After the reaction mixture was stirred at 130 °C for 30 min, it was allowed to cool to RT and diluted with DCM (2 mL). After sonicating the sample for 1 h at RT, the reaction mixture was directly purified by column chromatography to afford compound **8**.

Yellow solid (30 mg, 53%); TLC R_f = 0.3 (*n*-hexane:Et_2O = 8:1); **^{1}H NMR** (400 MHz, $CDCl_3$) δ 9.26 (d, *J* = 2.2 Hz, 1H), 9.22 – 9.17 (m, 1H), 8.93 – 8.86 (m, 2H), 8.62 – 8.54 (m, 2H), 7.88 – 7.83 (m, 1H), 7.82 – 7.67 (m, 2H), 1.53 (s, 9H); **^{13}C NMR** (100 MHz, $CDCl_3$) δ 151.32, 143.81, 143.72, 142.21, 142.10, 131.93, 130.06, 130.02, 130.00, 129.62, 128.06, 127.76, 125.79, 123.13, 123.07, 121.77, 35.65, 31.93; **ESI HRMS**: m/z $[M+H]^+$ calcd for $C_{20}H_{19}N_2$: 287.1543; found: 287.1542.

6-Phenyldibenzo[*f,h*]quinoxaline (9)

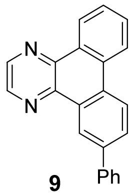

9

In an Ar-filled glove box, a screw-cap 1-dram vial was charged with cyclic diaryliodonium salt **2g** (0.20 mmol, 1.0 equiv.), KO*t*-Bu (0.60 mmol, 3.0 equiv.) and pyrazine (4.0 mmol, 20 equiv.). After the reaction mixture was stirred at 130 °C for 30 min, it was allowed to cool to RT and diluted with DCM (2 mL). After sonicating the sample for 1 h at RT, the reaction mixture was directly purified by column chromatography to afford compound **9**.

Yellow solid (25 mg, 41%); TLC R_f = 0.5 (*n*-hexane:Et_2O = 4:1); **^{1}H NMR** (400 MHz, $CDCl_3$) δ 9.47 (d, *J* = 2.1 Hz, 1H), 9.24 – 9.18 (m, 1H), 8.91 (s, 2H), 8.69 – 8.58 (m, 2H), 8.04 (dd, *J* = 8.5, 2.1 Hz, 1H), 7.89 – 7.85 (m, 2H), 7.77 (m, 2H), 7.56 – 7.50 (m, 2H), 7.46 – 7.40 (m, 1H); **^{13}C NMR** (100 MHz, $CDCl_3$) δ 144.04, 143.93, 142.12, 141.94, 140.87, 140.67, 131.68, 130.86, 130.63, 130.26, 130.10, 129.38, 128.89, 128.15, 128.13, 127.85, 125.86, 123.92, 123.85, 123.23; **ESI HRMS**: m/z $[M+H]^+$ calcd for $C_{22}H_{15}N_2$: 307.1230; found: 307.1230.

5,7-Dimethyldibenzo[*f,h*]quinoxaline (10)

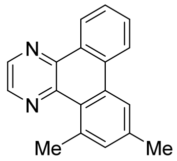

10

In an Ar-filled glove box, a screw-cap 1-dram vial was charged with cyclic diaryliodonium salt **2h** (0.20 mmol, 1.0 equiv.), KO*t*-Bu (0.60 mmol, 3.0 equiv.) and pyrazine (4.0 mmol, 20 equiv.). After the reaction mixture was stirred at 130 °C for 30 min, it was allowed to cool to RT and diluted with DCM (2 mL). After sonicating the sample for 1 h at RT, the reaction mixture was directly purified by column chromatography to afford compound **10**.

Yellow solid (24 mg, 47%); TLC R_f = 0.5 (*n*-hexane:Et_2O = 10:1); **^{1}H NMR** (400 MHz, $CDCl_3$) δ 9.19 (d, *J* = 1.7 Hz, 1H), 8.90 (d, *J* = 2.0 Hz, 1H), 8.80 (d, *J* = 2.1 Hz, 1H), 8.63 (d, *J* = 6.8 Hz, 1H), 8.38 (s, 1H), 7.78 – 7.66 (m, 2H), 7.40 (s, 1H), 3.21 (s, 3H), 2.60 (s, 3H); **^{13}C NMR** (100 MHz, CDCl3) δ 144.70, 142.18, 142.05, 141.68, 140.12, 138.84, 134.13, 133.29, 132.35, 130.63, 129.90, 127.89, 126.35, 125.71, 123.62, 121.78, 27.62, 22.27; **ESI HRMS**: m/z $[M+H]^+$ calcd for $C_{18}H_{15}N_2$: 259.1230; found: 259.1228.

Benzo[*f*]naphtho[2,1-*h*]quinoxaline (11)

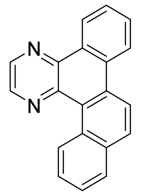

11

In an Ar-filled glove box, a screw-cap 1-dram vial was charged with cyclic diaryliodonium salt **2i** (0.20 mmol, 1.0 equiv.), KO*t*-Bu (0.60 mmol, 3.0 equiv.) and pyrazine (4.0 mmol, 20 equiv.). After the reaction mixture was stirred at 130 °C for 30 min, it was allowed to cool to RT and diluted with DCM (2 mL). After sonicating the sample for 1 h at RT, the reaction mixture was directly purified by column chromatography to afford compound **11**.

White solid (18 mg, 32%); TLC R_f = 0.3 (*n*-hexane:Et_2O = 10:1); **^{1}H NMR** (400 MHz, $CDCl_3$) δ 9.37 – 9.30 (m, 1H), 9.23 (d, *J* = 8.7 Hz, 1H), 9.08 – 9.02 (m, 1H), 8.97 (m, 3H), 8.12 – 8.03 (m, 2H), 7.85 – 7.74 (m, 2H), 7.72 – 7.62 (m, 2H); **^{13}C NMR** (100 MHz, $CDCl_3$) δ 143.56, 143.26, 141.04, 140.82, 134.73, 130.96, 130.56, 129.63, 129.19, 128.53, 128.43, 128.39, 128.35, 128.25, 127.90, 126.96, 126.61, 126.20, 125.09, 121.57; **ESI HRMS**: m/z $[M+H]^+$ calcd for $C_{20}H_{13}N_2$: 281.1073; found: 281.1073.

Benzo[*f*]pyrido[3,2-*h*]quinoxaline (12)

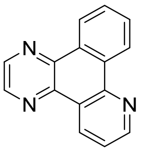

12

In an Ar-filled glove box, a screw-cap 1-dram vial was charged with cyclic diaryliodonium salt **2j** (0.20 mmol, 1.0 equiv.), KO*t*-Bu (0.60 mmol, 3.0 equiv.) and pyrazine (4.0 mmol, 20 equiv.). After the reaction mixture was stirred at 130 °C for 30 min, it was allowed to cool to RT and diluted with DCM (2 mL). After sonicating the sample for 1 h at RT, the reaction mixture was directly purified by column chromatography to afford compound **12**.

White solid (29 mg, 63%); TLC R_f = 0.3 (*n*-hexane:Et_2O = 4:1); **^{1}H NMR** (400 MHz, $CDCl_3$) δ 9.40 (dd, *J* = 8.1, 1.8 Hz, 1H), 9.29 – 9.24 (m, 1H), 9.19 – 9.14 (m, 1H), 9.08 (dd, *J* = 4.4, 1.8 Hz, 1H), 8.92 (dd, *J* = 15.7, 2.1 Hz, 2H), 7.91 – 7.81 (m, 2H), 7.66 (dd, *J* = 8.1, 4.4 Hz, 1H); **^{13}C NMR** (100 MHz, $CDCl_3$) δ 151.55, 148.32, 144.59, 144.13, 142.08, 141.04, 133.54, 132.95, 131.57, 130.33, 129.78, 125.42, 125.19, 125.11, 123.16; **ESI HRMS**: m/z $[M+H]^+$ calcd for $C_{15}H_{10}N_3$: 232.0869; found: 232.0865.

Benzo[*c*]pyrazino[2,3-*a*]phenanthridine (13)

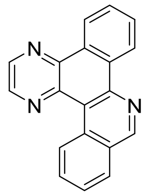

13

In an Ar-filled glove box, a screw-cap 1-dram vial was charged with cyclic diaryliodonium salt **2k** (0.20 mmol, 1.0 equiv.), KO*t*-Bu (0.60 mmol, 3.0 equiv.) and pyrazine (4.0 mmol, 20 equiv.). After the reaction mixture was stirred at 130 °C for 30 min, it was allowed to cool to RT and diluted with DCM (2 mL). After sonicating the sample for 1 h at RT, the reaction mixture was directly purified by column chromatography to afford compound **13**.

White solid (48 mg, 85%); TLC R_f = 0.3 (*n*-hexane:Et_2O = 4:1); **^{1}H NMR** (400 MHz, $CDCl_3$) δ 10.70 (dt, *J* = 8.8, 0.9 Hz, 1H), 9.54 (d, *J* = 0.9 Hz, 1H), 9.44 (d, *J* = 8.3 Hz, 1H), 9.24 (d, *J* = 1.5 Hz, 1H), 9.07 (d, *J* = 2.0 Hz, 1H), 8.97 (d, *J* = 2.0 Hz, 1H), 8.16 (d, *J* = 1.5 Hz, 1H), 8.00 – 7.82 (m, 3H), 7.76 (ddd, *J* = 8.0, 6.9, 1.1 Hz, 1H); **^{13}C NMR** (100 MHz, $CDCl_3$) δ 155.16, 144.89, 143.56, 142.84, 142.69, 142.30, 134.55, 133.59, 132.32, 131.29, 130.21, 129.52, 129.22, 129.19, 128.68, 127.76, 126.02, 124.86, 118.31; **ESI HRMS**: m/z $[M+H]^+$ calcd for $C_{19}H_{12}N_3$: 282.1026; found: 282.1024.

Benzo[*k*]pyrazino[2,3-*i*]phenanthridine (14)

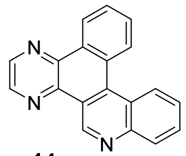

14

In an Ar-filled glove box, a screw-cap 1-dram vial was charged with cyclic diaryliodonium salt **2l** (0.20 mmol, 1.0 equiv.), KO*t*-Bu (0.60 mmol, 3.0 equiv.) and pyrazine (4.0 mmol, 20 equiv.). After the reaction mixture was stirred at 130 °C for 30 min, it was allowed to cool to RT and diluted with DCM (2 mL). After sonicating the sample for 1 h at RT, the reaction mixture was directly purified by column chromatography to afford compound **14**.

Yellow solid (32 mg, 57%); TLC R_f = 0.3 (*n*-hexane:Et_2O = 1:3); **^{1}H NMR** (400 MHz, $CDCl_3$) δ 10.56 (s, 1H), 9.40 – 9.21 (m, 1H), 8.98 – 8.77 (m, 4H), 8.35 (dd, *J* = 8.3, 1.4 Hz, 1H), 7.88 – 7.78 (m, 3H), 7.71 (ddd, *J* = 8.4, 6.9, 1.5 Hz, 1H); **^{13}C NMR** (100 MHz, $CDCl_3$) δ 148.27, 148.17, 144.14, 143.88, 140.86, 139.82, 133.82, 132.13, 130.24, 129.19, 129.16, 128.89, 128.79, 128.65, 126.96, 126.85, 125.30, 123.40, 121.51; **ESI HRMS**: m/z $[M+H]^+$ calcd for $C_{19}H_{12}N_3$: 282.1026; found: 282.1024.

Benzo[*f*]thieno[2,3-*h*]quinoxaline (15)

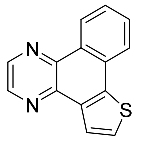

15

In an Ar-filled glove box, a screw-cap 1-dram vial was charged with cyclic diaryliodonium salt **2m** (0.20 mmol, 1.0 equiv.), KO*t*-Bu (0.60 mmol, 3.0 equiv.) and pyrazine (4.0 mmol, 20 equiv.). After the reaction mixture was stirred at 130 °C for 30 min, it was allowed to cool to RT and diluted with DCM (2 mL). After sonicating the sample for 1 h at RT, the reaction mixture was directly purified by column chromatography to afford compound **15**.

White solid (26 mg, 55%); TLC R_f = 0.3 (*n*-hexane:Et_2O = 4:1); **^{1}H NMR** (400 MHz, $CDCl_3$) δ 9.26 – 9.21 (m, 1H), 8.94 – 8.88 (m, 2H), 8.28 (d, *J* = 5.3 Hz, 1H), 8.19 – 8.14 (m, 1H), 7.80 – 7.70 (m, 2H), 7.63 (d, *J* = 5.3 Hz, 1H); **^{13}C NMR** (100 MHz, $CDCl_3$) δ 144.07, 142.99, 141.53, 140.83, 140.56, 135.69, 130.14, 130.05, 129.23, 127.53, 126.25, 126.19, 124.88, 124.17; **ESI HRMS**: m/z $[M+H]^+$ calcd for $C_{14}H_9N_2S$: 237.0481; found: 237.0479.

2,3-Dimethyldibenzo[*f*,*h*]quinoxaline (17)

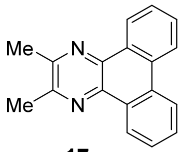

17

In an Ar-filled glove box, a screw-cap 1-dram vial was charged with cyclic diaryliodonium salt **2a** (0.20 mmol, 1.0 equiv.), KO*t*-Bu (0.60 mmol, 3.0 equiv.) and 2,3-dimethylpyrazine (8.0 mmol, 40 equiv.). After the reaction mixture was stirred at 130 °C for 1 h, it was allowed to cool to RT and diluted with DCM (2 mL). After sonicating the sample for 1 h at RT, the reaction mixture was directly purified by column chromatography to afford compound **17**.

Yellow solid (19 mg, 37%); TLC R_f = 0.3 (*n*-hexane:Et_2O = 20:1); **^{1}H NMR** (400 MHz, $CDCl_3$) δ 9.27 – 9.20 (m, 2H), 8.66 – 8.61 (m, 2H), 7.74 (m, 4H), 2.82 (s, 6H); **^{13}C NMR** (100 MHz, $CDCl_3$) δ 152.22, 138.80, 131.37, 130.54, 129.10, 127.85, 125.38, 123.12, 23.31; **ESI HRMS**: m/z $[M+H]^+$ calcd for $C_{18}H_{15}N_2$: 259.1230; found: 259.1229.

2-Ethyl-3-methyldibenzo[*f*,*h*]quinoxaline (18)

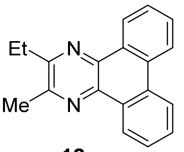

18

In an Ar-filled glove box, a screw-cap 1-dram vial was charged with cyclic diaryliodonium salt **2a** (0.20 mmol, 1.0 equiv.), KO*t*-Bu (0.60 mmol, 3.0 equiv.) and 2-ethyl-3-methyl-pyrazine (8.0 mmol, 40 equiv.). After the reaction mixture was stirred at 130 °C for 1 h, it was allowed to cool to RT and diluted with DCM (2 mL). After sonicating the sample for 1 h at RT, the reaction mixture was directly purified by column chromatography to afford compound **18**.

Yellow solid (13 mg, 23%); TLC R_f = 0.4 (*n*-hexane:Et_2O = 20:1); **^{1}H NMR** (400 MHz, $CDCl_3$) δ 9.31 – 9.14 (m, 2H), 8.69 – 8.53 (m, 2H), 7.81 – 7.59 (m, 4H), 3.10 (q, *J* = 7.5 Hz, 2H), 2.83 (d, *J* = 1.0 Hz, 3H), 1.53 (t, *J* = 7.5 Hz, 3H); **^{13}C NMR** (100 MHz, $CDCl_3$) δ 156.16, 151.80, 138.76, 138.45, 131.36, 130.80, 130.59, 129.19, 129.03, 127.82, 127.81, 127.58, 125.51, 125.37, 123.12, 123.08, 28.96, 22.80, 12.31; **ESI HRMS**: m/z $[M+H]^+$ calcd for $C_{19}H_{17}N_2$: 273.1386; found: 273.1385.

Dibenzo[*f*,*h*]quinoxaline-2-carbonitrile (19)

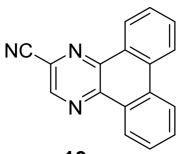

19

In an Ar-filled glove box, a screw-cap 1-dram vial was charged with cyclic diaryliodonium salt **2a** (0.20 mmol, 1.0 equiv.), KO*t*-Bu (0.60 mmol, 3.0 equiv.) and pyrazinecarbonitrile (8.0 mmol, 40 equiv.). After the reaction mixture was stirred at 130 °C for 1 h, it was allowed to cool to RT and diluted with DCM (2 mL). After sonicating the sample for 1 h at RT, the reaction mixture was directly purified by column chromatography to afford compound **19**.

Yellow solid (14 mg, 27%); TLC R_f = 0.4 (*n*-hexane:Et_2O = 4:1); **^{1}H NMR** (400 MHz, $CDCl_3$) δ 9.16 (m, 2H), 9.12 (s, 1H), 8.60 (m, 2H), 7.89 – 7.82 (m, 2H), 7.79 – 7.72 (m, 2H); **^{13}C NMR** (100 MHz, $CDCl_3$) δ 145.24, 143.33, 142.84, 141.46, 132.48, 131.73, 131.24, 130.76, 129.42, 128.41, 128.18, 128.15, 128.03, 127.95, 127.53, 126.34, 125.82, 125.20, 122.81, 122.67, 122.57, 116.29; **ESI HRMS**: m/z $[M+H]^+$ calcd for $C_{17}H_{10}N_3$: 256.0869; found: 256.0870.

2-(Trifluoromethyl)dibenzo[*f*,*h*]quinoxaline (20)

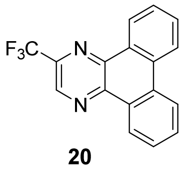

20

In an Ar-filled glove box, a screw-cap 1-dram vial was charged with cyclic diaryliodonium salt **2a** (0.20 mmol, 1.0 equiv.), KO*t*-Bu (0.60 mmol, 3.0 equiv.) and 2-(trifluoromethyl)pyrazine (8.0 mmol, 40 equiv.). After the reaction mixture was stirred at 130 °C for 1 h, it was allowed to cool to RT and diluted with DCM (2 mL). After sonicating the sample for 1 h at RT, the reaction mixture was directly purified by column chromatography to afford compound **20**.

Yellow solid (15 mg, 25%); TLC R_f = 0.4 (*n*-hexane:Et_2O = 20:1); **^{1}H NMR** (400 MHz, $CDCl_3$) δ 9.26 – 9.05 (m, 3H), 8.62 (m, 2H), 7.91 – 7.80 (m, 2H), 7.77 (m, 2H); **^{13}C NMR** (100 MHz, $CDCl_3$) δ 143.93, 142.25, 141.90, 140.91, 140.05, 140.02, 139.99, 139.96, 132.76, 132.35, 131.25, 131.01, 129.39, 128.50, 126.61, 126.50, 123.45, 123.38, 123.26, 120.72; **^{19}F NMR** (376 MHz, $CDCl_3$) δ -66.70; **ESI HRMS**: m/z $[M+H]^+$ calcd for $C_{17}H_{10}F_3N_2$: 299.0791; found: 299.0789.

4-Methyldibenzo[*f*,*h*]quinoline (21)

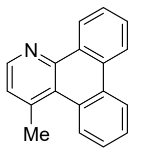

21

In an Ar-filled glove box, a screw-cap 1-dram vial was charged with cyclic diaryliodonium salt **2a** (0.20 mmol, 1.0 equiv.), KO*t*-Bu (0.60 mmol, 3.0 equiv.) and 4-methylpyridine (8.0 mmol, 40 equiv.). After the reaction mixture was stirred at 130 °C for 1 h, it was allowed to cool to RT and diluted with DCM (2 mL). After sonicating the sample for 1 h at RT, the reaction mixture was directly purified by column chromatography to afford compound **21**.

Yellow solid (10 mg, 21%); TLC R_f = 0.3 (*n*-hexane:Et_2O = 10:1); **^{1}H NMR** (400 MHz, $CDCl_3$) δ 9.34 – 9.28 (m, 1H), 8.80 (d, *J* = 4.6 Hz, 1H), 8.71 (dd, *J* = 8.1, 1.5 Hz, 1H), 8.65 – 8.55 (m, 2H), 7.76 – 7.67 (m, 3H), 7.63 (m, 1H), 7.41 – 7.38 (m, 1H), 3.08 (s, 3H); **^{13}C NMR** (100 MHz, $CDCl_3$) δ 131.77, 131.34, 129.45, 128.99, 128.11, 127.78, 126.66, 126.51, 126.36, 123.96, 122.77, 26.77; **ESI HRMS**: m/z $[M+H]^+$ calcd for $C_{18}H_{14}N$: 244.1121; found: 244.1120.

4-Phenyldibenzo[*f*,*h*]quinoline (22)

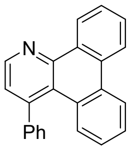

22

In an Ar-filled glove box, a screw-cap 1-dram vial was charged with cyclic diaryliodonium salt **2a** (0.20 mmol, 1.0 equiv.), KO*t*-Bu (0.60 mmol, 3.0 equiv.) and 4-phenylpyridine (8.0 mmol, 40 equiv.). After the reaction mixture was stirred at 130 °C for 1 h, it was allowed to cool to RT and diluted with DCM (2 mL). After sonicating the sample for 1 h at RT, the reaction mixture was directly purified by column chromatography to afford compound **22**.

Yellow solid (9 mg, 14%); TLC R_f = 0.5 (*n*-hexane:Et_2O = 3:1); **^{1}H NMR** (400 MHz, $CDCl_3$) δ 9.38 – 9.34 (m, 1H), 8.92 (d, *J* = 4.5 Hz, 1H), 8.59 (m, 2H), 7.77 – 7.73 (m, 2H), 7.70 – 7.66 (m, 1H), 7.54 – 7.41 (m, 6H), 7.39 (d, *J* = 4.5 Hz, 1H), 7.10 (m, 1H); **^{13}C NMR** (100 MHz, $CDCl_3$) δ 148.00, 147.82, 147.39, 142.65, 131.39, 130.94, 130.79, 129.57, 129.08, 128.82, 128.53, 128.34, 127.89, 127.41, 127.09, 125.66, 125.20, 124.94, 123.09, 123.03, 122.19; **ESI HRMS**: m/z $[M+H]^+$ calcd for $C_{23}H_{16}N$: 306.1277; found: 306.1276.

Dibenzo[*f*,*h*]quinoline-4-carbonitrile (23)

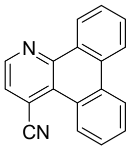

23

In an Ar-filled glove box, a screw-cap 1-dram vial was charged with cyclic diaryliodonium salt **2a** (0.20 mmol, 1.0 equiv.), KO*t*-Bu (0.60 mmol, 3.0 equiv.) and 4-pyridinecarbonitrile (8.0 mmol, 40 equiv.). After the reaction mixture was stirred at 130 °C for 1 h, it was allowed to cool to RT and diluted with DCM (2 mL). After sonicating the sample for 1 h at RT, the reaction mixture was directly purified by column chromatography to afford compound **23**.

Yellow solid (18 mg, 35%); TLC R_f = 0.3 (*n*-hexane:Et_2O = 4:1); **^{1}H NMR** (400 MHz, $CDCl_3$) δ 9.61 – 9.54 (m, 1H), 9.33 – 9.27 (m, 1H), 9.05 (d, *J* = 4.5 Hz, 1H), 8.72 (dd, *J* = 8.1, 1.5 Hz, 1H), 8.62 (dd, *J* = 8.1, 1.2 Hz, 1H), 7.88 (d, *J* = 4.5 Hz, 1H), 7.84 – 7.70 (m, 4H); **^{13}C NMR** (100 MHz, $CDCl_3$) δ 148.78, 148.07, 132.06, 131.41, 130.57, 130.55, 130.04, 128.48, 128.18, 128.15, 127.17, 126.45, 124.09, 123.94, 123.01, 119.93, 115.88; **ESI HRMS**: m/z $[M+H]^+$ calcd for $C_{18}H_{11}N_2$: 255.0917; found: 255.0915.

Dibenzo[*f*,*h*]quinoline-3-carbonitrile (23')

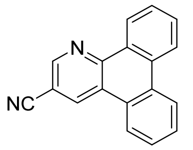

23'

In an Ar-filled glove box, a screw-cap 1-dram vial was charged with cyclic diaryliodonium salt **2a** (0.20 mmol, 1.0 equiv.), KO*t*-Bu (0.60 mmol, 3.0 equiv.) and nicotinonitrile (8.0 mmol, 40 equiv.). After the reaction mixture was stirred at 130 °C for 1 h, it was allowed to cool to RT and diluted with DCM (2 mL). After sonicating the sample for 1 h at RT, the reaction mixture was directly purified by column chromatography to afford compound **23'**.

White solid (11 mg, 21%); TLC R_f = 0.5 (*n*-hexane:EtOAc = 9:1); **^{1}H NMR** (400 MHz, $CDCl_3$) δ 9.31 (dd, *J* = 8.0, 1.6 Hz, 1H), 9.14 (s, *J* = 0.9 Hz, 2H), 8.73 – 8.67 (m, 1H), 8.64 (d, *J* = 8.2 Hz, 1H), 8.53 (dd, *J* = 8.1, 1.3 Hz, 1H), 7.85 (ddt, *J* = 8.2, 7.0, 1.3 Hz, 1H), 7.83 – 7.71 (m, 3H); **^{13}C NMR** (100 MHz, $CDCl_3$) δ 149.79, 148.78, 135.01, 132.55, 130.73, 130.34, 129.79, 129.28, 128.17, 128.14, 127.15, 126.46, 123.83, 123.77, 123.51, 122.89, 117.65, 107.58; **ESI HRMS**: m/z $[M+H]^+$ calcd for $C_{18}H_{11}N_2$: 255.0917; found: 255.0914.

Dibenzo[*a*,*c*]phenazine (24)

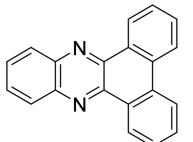

24

In an Ar-filled glove box, a screw-cap 1-dram vial was charged with cyclic diaryliodonium salt **2a** (0.20 mmol, 1.0 equiv.), KO*t*-Bu (0.60 mmol, 3.0 equiv.) and quinoxaline (8.0 mmol, 40 equiv.). After the reaction mixture was stirred at 130 °C for 1 h, it was allowed to cool to RT and diluted with DCM (2 mL). After sonicating the sample for 1 h at RT, the reaction mixture was directly purified by column chromatography to afford compound **24**.

Yellow solid (30 mg, 53%); TLC R_f = 0.5 (*n*-hexane:Et_2O = 10:1); **^{1}H NMR** (400 MHz, $CDCl_3$) δ 9.43 – 9.33 (m, 2H), 8.60 – 8.47 (m, 2H), 8.32 (dd, *J* = 6.5, 3.4 Hz, 2H), 7.93 – 7.65 (m, 6H).

The NMR spectrum of compound **24** matched previously reported literature data[13].

1.6.2. Additional Reaction Scope: Compound 24 Derivatives

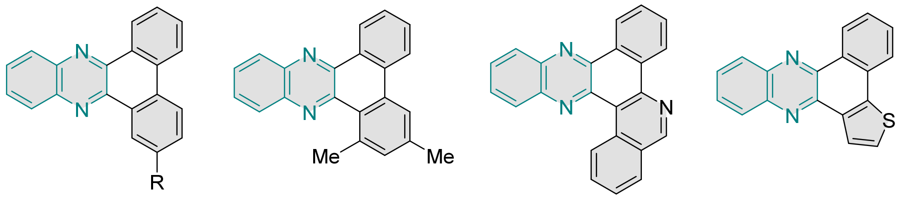

24a, R = Me, 25%
24b, R = Ph, 31%
24c, 34%
24d, 30%
24e, 23%

2-Methyldibenzo[*a,c*]phenazine (24a)

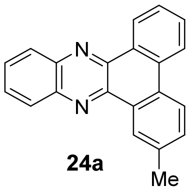

24a

In an Ar-filled glove box, a screw-cap 1-dram vial was charged with cyclic diaryliodonium salt **2e** (0.20 mmol, 1.0 equiv.), KO*t*-Bu (0.60 mmol, 3.0 equiv.) and quinoxaline (8.0 mmol, 40 equiv.). After the reaction mixture was stirred at 130 °C for 1 h, it was allowed to cool to RT and diluted with DCM (2 mL). After sonicating the sample for 1 h at RT, the reaction mixture was directly purified by column chromatography to afford compound **24a**.

Yellow solid (15 mg, 25%); TLC R_f = 0.5 (*n*-hexane:DCM = 3:2); **^{1}H NMR** (400 MHz, $CDCl_3$) δ 9.35 (dd, *J* = 7.8, 1.6 Hz, 1H), 9.16 – 9.12 (s, 1H), 8.48 (d, *J* = 7.8 Hz, 1H), 8.39 (d, *J* = 8.3 Hz, 1H), 8.36 – 8.26 (m, 2H), 7.89 – 7.79 (m, 2H), 7.72 (m, 2H), 7.57 (dd, *J* = 8.3, 1.9 Hz, 1H), 2.64 (s, 3H); **^{13}C NMR** (100 MHz, $CDCl_3$) δ 142.73, 142.57, 142.23, 142.22, 138.04, 132.30, 131.76, 130.36, 130.27, 130.03, 129.81, 129.78, 129.75, 129.58, 129.50, 127.59, 126.33, 126.20, 123.00, 122.79, 21.76; **ESI HRMS**: m/z $[M+H]^+$ calcd for $C_{21}H_{15}N_2$: 295.1230; found: 295.1233.

2-Phenyldibenzo[*a,c*]phenazine (24b)

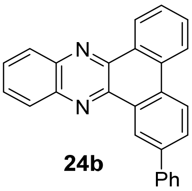

24b

In an Ar-filled glove box, a screw-cap 1-dram vial was charged with cyclic diaryliodonium salt **2g** (0.20 mmol, 1.0 equiv.), KO*t*-Bu (0.60 mmol, 3.0 equiv.) and quinoxaline (8.0 mmol, 40 equiv.). After the reaction mixture was stirred at 130 °C for 1 h, it was allowed to cool to RT and diluted with DCM (2 mL). After sonicating the sample for 1 h at RT, the reaction mixture was directly purified by column

chromatography to afford compound **24b**.

Yellow solid (22 mg, 31%); TLC R_f = 0.5 (*n*-hexane:DCM = 3:2); **^{1}H NMR** (400 MHz, $CDCl_3$) δ 9.67 (d, *J* = 2.1 Hz, 1H), 9.43 (dd, *J* = 7.9, 1.6 Hz, 1H), 8.64 (d, *J* = 8.5 Hz, 1H), 8.61 – 8.55 (m, 1H), 8.41 – 8.29 (m, 2H), 8.05 (dd, *J* = 8.4, 2.1 Hz, 1H), 7.93 – 7.90 (m, 2H), 7.87 (dt, *J* = 6.5, 3.4 Hz, 2H), 7.84 – 7.80 (m, 1H), 7.76 (ddd, *J* = 8.2, 7.1, 1.3 Hz, 1H), 7.57 (dd, *J* = 8.4, 7.0 Hz, 2H), 7.48 – 7.42 (m, 1H); **^{13}C NMR** (100 MHz, $CDCl_3$) δ 142.88, 142.74, 142.50, 142.46, 140.85, 140.81, 132.16, 131.33, 130.94, 130.63, 130.56, 130.08, 130.04, 129.77, 129.72, 129.38, 129.19, 128.18, 127.97, 127.67, 126.57, 124.75, 123.82, 123.22; **ESI HRMS**: m/z $[M+H]^+$ calcd for $C_{26}H_{17}N_2$: 357.1386; found: 357.1391.

1,3-Dimethyldibenzo[*a,c*]phenazine (24c)

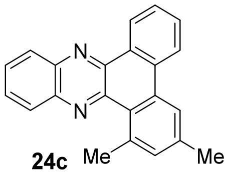

24c

In an Ar-filled glove box, a screw-cap 1-dram vial was charged with cyclic diaryliodonium salt **2h** (0.20 mmol, 1.0 equiv.), KO*t*-Bu (0.60 mmol, 3.0 equiv.) and quinoxaline (8.0 mmol, 40 equiv.). After the reaction mixture was stirred at 130 °C for 1 h, it was allowed to cool to RT and diluted with DCM (2 mL). After sonicating the sample for 1 h at RT, the reaction mixture was directly purified by column chromatography to afford compound **24c**.

Yellow solid (21 mg, 34%); TLC R_f = 0.5 (*n*-hexane:DCM = 3:2); **^{1}H NMR** (400 MHz, $CDCl_3$) δ 9.37 – 9.32 (m, 1H), 8.50 (dd, *J* = 8.1, 1.4 Hz, 1H), 8.32 – 8.21 (m, 3H), 7.86 – 7.77 (m, 2H), 7.75 – 7.63 (m, 2H), 7.37 – 7.30 (m, 1H), 3.32 (s, 3H), 2.57 (s, 3H); **^{13}C NMR** (100 MHz, $CDCl_3$) δ 145.32, 142.78, 141.30, 140.93, 140.33, 139.19, 134.01, 133.47, 132.76, 130.64, 130.28, 129.52, 129.51, 129.27, 129.24, 127.75, 126.27, 126.21, 123.41, 121.82, 27.63, 21.95; **ESI HRMS**: m/z $[M+H]^+$ calcd for $C_{22}H_{17}N_2$: 309.1386; found: 309.1394.

Benzo[*a*]isoquinolino[3,4-*c*]phenazine (24d)

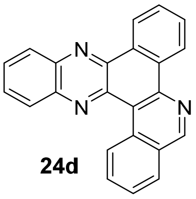

24d

In an Ar-filled glove box, a screw-cap 1-dram vial was charged with cyclic diaryliodonium salt **2k** (0.20 mmol, 1.0 equiv.), KO*t*-Bu (0.60 mmol, 3.0 equiv.) and quinoxaline (8.0 mmol, 40 equiv.). After the reaction mixture was stirred at 130 °C for 1 h, it was allowed to cool to RT and diluted with DCM (2 mL). After sonicating the sample for 1 h at RT, the reaction mixture was directly purified by column chromatography to afford compound **24d**.

Yellow solid (20 mg, 30%); TLC R_f = 0.2 (*n*-hexane:DCM = 1:2); **^{1}H NMR** (400 MHz, $CDCl_3$) δ 10.99 (d, *J* = 8.9 Hz, 1H), 9.51 (s, 1H), 9.37 (ddd, *J* = 10.9, 7.8, 1.5 Hz, 2H), 8.46 – 8.38 (m, 1H), 8.37 – 8.32 (m, 1H), 8.14 (d, *J* = 8.0 Hz, 1H), 8.00 (ddd, *J* = 8.7, 6.9, 1.5 Hz, 1H), 7.94 – 7.80 (m, 4H), 7.75 (ddd, *J* = 8.0, 6.8, 1.1 Hz, 1H); **^{13}C NMR** (100 MHz, $CDCl_3$) δ 155.18, 145.75, 144.38, 142.53, 141.43, 140.84, 134.55, 133.86, 132.30, 131.27, 130.50, 130.31, 129.94, 129.54, 129.51, 129.26, 129.08, 128.88, 128.57, 127.40, 125.99, 125.39, 118.09; **ESI HRMS**: m/z $[M+H]^+$ calcd for $C_{23}H_{14}N_2$: 332.1182; found: 332.1185.

Benzo[*a*]thieno[2,3-*c*]phenazine (24e)

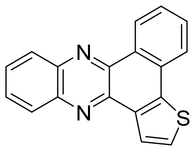

24e

In an Ar-filled glove box, a screw-cap 1-dram vial was charged with cyclic diaryliodonium salt **2m** (0.20 mmol, 1.0 equiv.), KO*t*-Bu (0.60 mmol, 3.0 equiv.) and quinoxaline (8.0 mmol, 40 equiv.). After the reaction mixture was stirred at 130 °C for 1 h, it was allowed to cool to RT and diluted with DCM (2 mL). After sonicating the sample for 1 h at RT, the reaction mixture was directly purified by column chromatography to afford compound **24e**.

Yellow solid (13 mg, 23%); TLC R_f = 0.4 (*n*-hexane:DCM = 1:1); **^{1}H NMR** (400 MHz, $CDCl_3$) δ 9.42 – 9.37 (m, 1H), 8.41 (d, *J* = 5.3 Hz, 1H), 8.37 – 8.29 (m, 2H), 8.13 – 8.07 (m, 1H), 7.87 (m, 2H), 7.80 – 7.69 (m, 2H), 7.60 (d, *J* = 5.3 Hz, 1H); **^{13}C NMR** (100 MHz, $CDCl_3$) δ 142.43, 142.36, 141.67, 141.37, 141.11, 135.65, 130.46, 130.20, 130.10, 129.84, 129.54, 129.36, 129.32, 127.45, 126.59, 125.55, 125.51, 123.95; **ESI HRMS**: m/z $[M+H]^+$ calcd for $C_{18}H_{11}N_2S$: 287.0637; found: 287.0641.

1.7. Synthesis of Triphenylene and Its Analogues

Triphenylene (25)

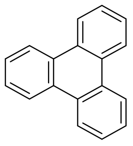

25

In an Ar-filled glove box, a screw-cap 1-dram vial was charged with cyclic diaryliodonium salt **2a** (0.20 mmol, 1.0 equiv.), KO*t*-Bu (1.0 mmol, 5.0 equiv.), pyrazine (0.40 mmol, 2.0 equiv.) and benzene (2 mL). After the reaction mixture was stirred at 110 °C for 3 h, it was allowed to cool to RT and diluted with DCM (2 mL). After sonicating the sample for 1 h at RT, the reaction mixture was directly purified by column chromatography to afford compound **25**.

White solid (22 mg, 48%); TLC R_f = 0.4 (*n*-hexane:Et_2O = 50:1); **^{1}H NMR** (400 MHz, $CDCl_3$) δ 8.67 (dd, *J* = 6.2, 3.4 Hz, 6H), 7.67 (dd, *J* = 6.3, 3.3 Hz, 6H).

The NMR spectrum of compound **25** matched previously reported literature data[3].

1,3-Dimethyltriphenylene (26)

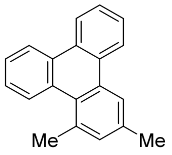

26

In an Ar-filled glove box, a screw-cap 1-dram vial was charged with cyclic diaryliodonium salt **2h** (0.20 mmol, 1.0 equiv.), KO*t*-Bu (1.0 mmol, 5.0 equiv.), pyrazine (0.40 mmol, 2.0 equiv.) and benzene (2 mL). After the reaction mixture was stirred at 110 °C for 3 h, it was allowed to cool to RT and diluted with DCM (2 mL). After sonicating the sample for 1 h at RT, the reaction mixture was directly purified by column chromatography to afford compound **26**.

White solid (17 mg, 34%); TLC R_f = 0.4 (*n*-hexane:Et_2O = 50:1); **^{1}H NMR** (400 MHz, $CDCl_3$) δ 8.64 – 8.57 (m, 4H), 8.33 (s, 1H), 7.63 – 7.54 (m, 4H), 7.35 (s, 1H), 3.03 (s, 3H), 2.57 (s, 3H).

The NMR spectrum of compound **26** matched previously reported literature data[3].

2-Phenyltriphenylene (27)

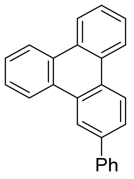

27

In an Ar-filled glove box, a screw-cap 1-dram vial was charged with cyclic diaryliodonium salt **2g** (0.20 mmol, 1.0 equiv.), KO*t*-Bu (1.0 mmol, 5.0 equiv.), pyrazine (0.40 mmol, 2.0 equiv.) and benzene (2 mL). After the reaction mixture was stirred at 110 °C for 3 h, it was allowed to cool to RT and diluted with DCM (2 mL). After sonicating the sample for 1 h at RT, the reaction mixture was directly purified by column chromatography to afford compound **27**.

White solid (20 mg, 36%); TLC R_f = 0.4 (*n*-hexane:Et_2O = 50:1); **^{1}H NMR** (400 MHz, $CDCl_3$) δ 8.87 (d, *J* = 1.9 Hz, 1H), 8.79 – 8.63 (m, 5H), 7.91 (dd, *J* = 8.5, 1.9 Hz, 1H), 7.83 – 7.78 (m, 2H), 7.73 – 7.65 (m, 4H), 7.54 (td, *J* = 8.5, 7.9, 1.9 Hz, 2H), 7.47 – 7.40 (m, 1H).

The NMR spectrum of compound **27** matched previously reported literature data[3].

Benzo[*g*]chrysene (28)

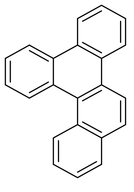

28

In an Ar-filled glove box, a screw-cap 1-dram vial was charged with cyclic diaryliodonium salt **2i** (0.20 mmol, 1.0 equiv.), KO*t*-Bu (1.0 mmol, 5.0 equiv.), pyrazine (0.40 mmol, 2.0 equiv.) and benzene (2 mL). After the reaction mixture was stirred at 110 °C for 3 h, it was allowed to cool to RT and diluted with DCM (2 mL). After sonicating the sample for 1 h at RT, the reaction mixture was directly purified by column chromatography to afford compound **28**.

White solid (14 mg, 25%); TLC R_f = 0.4 (*n*-hexane:Et_2O =50:1); **^{1}H NMR** (400 MHz, $CDCl_3$) δ 8.98 – 8.89 (m, 2H), 8.78 – 8.71 (m, 2H), 8.70 – 8.59 (m, 2H), 8.07 – 7.98 (m, 2H), 7.78 – 7.54 (m, 7H).

The NMR spectrum of compound **28** matched previously reported literature data[3].

Dibenzo[*f*,*h*]quinoline (29)

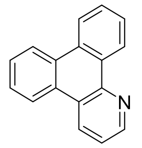

29

In an Ar-filled glove box, a screw-cap 1-dram vial was charged with cyclic diaryliodonium salt **2j** (0.20 mmol, 1.0 equiv.), KO*t*-Bu (1.0 mmol, 5.0 equiv.), pyrazine (0.40 mmol, 2.0 equiv.) and benzene (2 mL). After the reaction mixture was stirred at 110 °C for 3 h, it was allowed to cool to RT and diluted with DCM (2 mL). After sonicating the sample for 1 h at RT, the reaction mixture was directly purified by column chromatography to afford compound **29**.

White solid (24 mg, 52%); TLC R_f = 0.3 (*n*-hexane:Et_2O = 9:1); **^{1}H NMR** (400 MHz, $CDCl_3$) δ 9.38 – 9.29 (m, 1H), 8.98 (dd, *J* = 4.4, 1.7 Hz, 1H), 8.89 (dd, *J* = 8.3, 1.7 Hz, 1H), 8.72 – 8.54 (m, 3H), 7.80 – 7.65 (m, 4H), 7.59 (dd, *J* = 8.3, 4.4 Hz, 1H).

The NMR spectrum of compound **29** matched previously reported literature data[12].

Dibenzo[*i*,*k*]phenanthridine (30)

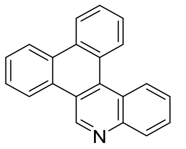

30

In an Ar-filled glove box, a screw-cap 1-dram vial was charged with cyclic diaryliodonium salt **2l** (0.20 mmol, 1.0 equiv.), KO*t*-Bu (1.0 mmol, 5.0 equiv.), pyrazine (0.40 mmol, 2.0 equiv.) and benzene (2 mL). After the reaction mixture was stirred at 110 °C for 3 h, it was allowed to cool to RT and diluted with DCM (2 mL). After sonicating the sample for 1 h at RT, the reaction mixture was directly purified by column chromatography to afford compound **30**.

White solid (16 mg, 43%); TLC R_f = 0.4 (*n*-hexane:Et_2O = 9:1); **^{1}H NMR** (400 MHz, $CDCl_3$) δ 9.46 (d, *J* = 0.8 Hz, 1H), 9.40 – 9.33 (m, 1H), 9.02 – 8.90 (m, 2H), 8.79 (dd, *J* = 8.1, 1.6 Hz, 1H), 8.72 – 8.65 (m, 1H), 8.19 (dd, *J* = 8.0, 1.4 Hz, 1H), 7.87 (ddd, *J* = 8.5, 6.9, 1.4 Hz, 1H), 7.82 – 7.66 (m, 5H); **^{13}C NMR** (100 MHz, $CDCl_3$) δ 152.17, 142.00, 133.16, 131.52, 131.44, 131.20, 131.06, 129.35, 129.25, 129.20, 128.98, 128.71, 128.66, 128.01, 127.62, 127.22, 127.09, 126.90, 125.92, 124.10, 122.79, 119.98; **ESI HRMS**: m/z $[M+H]^+$ calcd for $C_{21}H_{14}N$: 280.1121; found: 280.1120.

Dibenzo[*a*,*c*]phenanthridine (31)

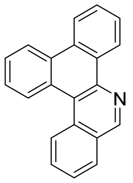

31

In an Ar-filled glove box, a screw-cap 1-dram vial was charged with cyclic diaryliodonium salt **2k** (0.20 mmol, 1.0 equiv.), KO*t*-Bu (1.0 mmol, 5.0 equiv.), pyrazine (0.40 mmol, 2.0 equiv.) and benzene (2 mL). After the reaction mixture was stirred at 110 °C for 3 h, it was allowed to cool to RT and diluted with DCM (2 mL). After sonicating the sample for 1 h at RT, the reaction mixture was directly purified by column chromatography to afford compound **31**.

White solid (24 mg, 29%); TLC R_f = 0.4 (*n*-hexane:Et_2O = 1:2); **^{1}H NMR** (400 MHz, $CDCl_3$) δ 10.17 (s, 1H), 9.00 (dd, *J* = 8.2, 1.3 Hz, 1H), 8.94 (dd, *J* = 8.5, 1.3 Hz, 1H), 8.88 – 8.70 (m, 3H), 8.38 (dd, *J* = 8.3, 1.4 Hz, 1H), 7.89 – 7.68 (m, 6H); **^{13}C NMR** (100 MHz, $CDCl_3$) δ 147.52, 147.38, 132.87, 132.54, 130.54, 130.24, 130.08, 129.48, 129.05, 128.87, 128.73, 128.44, 128.34, 128.28, 128.13, 127.94, 127.13, 124.53, 124.11, 123.72, 123.30, 122.35; **ESI HRMS**: m/z $[M+H]^+$ calcd for $C_{21}H_{14}N$: 280.1121; found: 280.1119.

Phenanthro[9,10-*b*]thiophene (32)

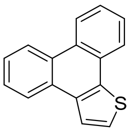

32

In an Ar-filled glove box, a screw-cap 1-dram vial was charged with cyclic diaryliodonium salt **2m** (0.20 mmol, 1.0 equiv.), KO*t*-Bu (1.0 mmol, 5.0 equiv.), pyrazine (0.40 mmol, 2.0 equiv.) and benzene (2 mL). After the reaction mixture was stirred at 110 °C for 3 h, it was allowed to cool to RT and diluted with DCM (2 mL). After sonicating the sample for 1 h at RT, the reaction mixture was directly purified by column chromatography to afford compound **32**.

White solid (25 mg, 53%); TLC R_f = 0.3 (*n*-hexane:Et_2O = 50:1); **^{1}H NMR** (400 MHz, $CDCl_3$) δ 8.74 – 8.63 (m, 2H), 8.36 – 8.30 (m, 1H), 8.20 – 8.12 (m, 1H), 7.98 (d, *J* = 5.3 Hz, 1H), 7.70 – 7.59 (m, 4H), 7.57 (d, *J* = 5.3 Hz, 1H).

The NMR spectrum of compound **32** matched previously reported literature data[14].

1.8. Access to an N-Doped Heptacyclic Nanographene Fragment (33)

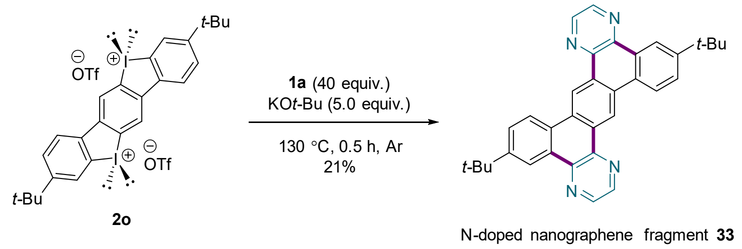

Supplementary Figure 3 Synthesis of compound **33**

In an Ar-filled glove box, a screw-cap 1-dram vial was charged with fused cyclic diaryliodonium salt **2o** (0.20 mmol, 1.0 equiv.), KO*t*-Bu (1.0 mmol, 5.0 equiv.) and pyrazine (8.0 mmol, 40 equiv.). The reaction mixture was stirred at 130 °C for 30 min, after the state of pyrazine changed from solid to liquid (few seconds). The reaction mixture was allowed to cool to RT and diluted with DCM (2 mL). After the sample was sonicated for 1 h at RT, it was directly purified by column chromatography to afford compound **33**.

Yellow solid (21 mg, 21%); TLC $R_f = 0.3$ (*n*-hexane:Et_2O = 4:1); **^{1}H NMR** (600 MHz, $C_2D_2Cl_4$) δ 10.51 (s, 2H), 9.36 (d, *J* = 2.2 Hz, 2H), 9.03 – 8.97 (m, 4H), 7.99 (dd, *J* = 8.5, 2.2 Hz, 2H), 6.98 (s, 2H), 1.62 (s, 18H). **^{13}C NMR** (150 MHz, $C_2D_2Cl_4$) δ 151.40, 143.72, 143.20, 142.66, 141.79, 130.38, 130.14, 129.79, 129.25, 127.61, 125.24, 123.39, 121.58, 119.70, 36.97, 31.34. **ESI HRMS**: m/z $[M+H]^+$ calcd for $C_{34}H_{31}N_4$: 495.2543; found: 495.2541.

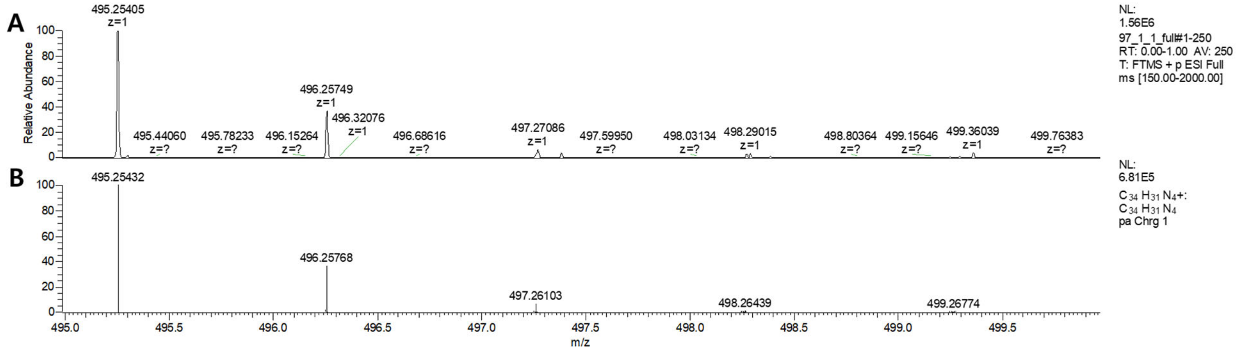

Supplementary Figure 4 Comparison of HRMS isotope distributions of (**A**) **33** and (**B**) calculated data.

1.9. Formation of 2,2′-Bipyrazine (34)

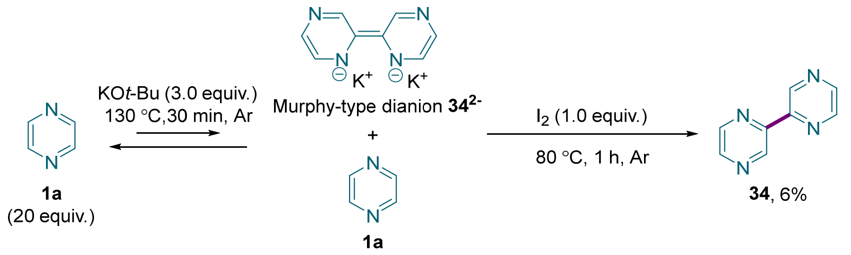

Supplementary Figure 5 Formation of compound **34**

To gain insight into the unidentified electron donor that can directly transfer an electron to cyclic diphenyliodonium salt **2a**, the reaction of **1a** with potassium *tert*-butoxide was conducted in the absence of **2a**. Following workup with I_2 as an external oxidant, 2,2′-bipyrazine (**34**) was isolated. This result is consistent with the formation of dianion $\mathbf{34}^{2-}$ prior to oxidation. Similar biazine scaffolds have been reported as super electron donors to initiate radical chain reactions[15].

Detailed reaction procedure (2,2′-bipyrazine, compound 34)

In an Ar-filled glove box, a screw-cap 1-dram vial was charged with KO*t*-Bu (3.0 mmol, 3.0 equiv.) and pyrazine (20 mmol, 20 equiv.). After the reaction mixture was stirred at 130 °C for 30 min, it was allowed to cool to 70 °C. The vial was brought back into an Ar-filled glove box and I_2 (1.0 mmol, 1.0 equiv.) was added. After the mixture was stirred for 1 h at 80 °C, it was allowed to cool to RT and diluted/quenched with DCM (5 mL). (**CAUTION!** If exposed to ambient atmosphere, the reaction mixture of the second step is potentially pyrophoric and a violent reaction may occur.) After the sample was sonicated for 1 h at RT, it was directly purified by column chromatography to afford the title compound.

White solid (10 mg, 6%); TLC R_f = 0.3 (*n*-hexane:Et_2O = 1:1); **^{1}H NMR** (400 MHz, $CDCl_3$) δ 9.61 (s, 2H), 8.67 (s, 4H).

The NMR spectrum of **34** matched previously reported literature data[16].

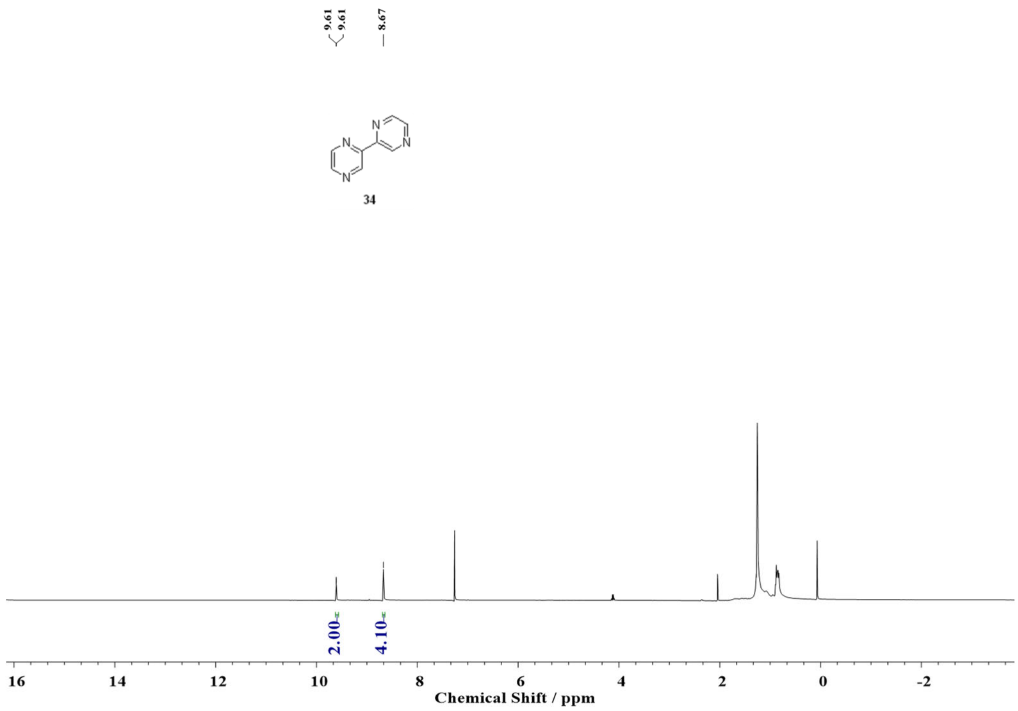

Supplementary Figure 6 ^{1}H NMR (400 MHz, $CDCl_3$) of compound **34**

1.10. Two-Step Annulation Experiments

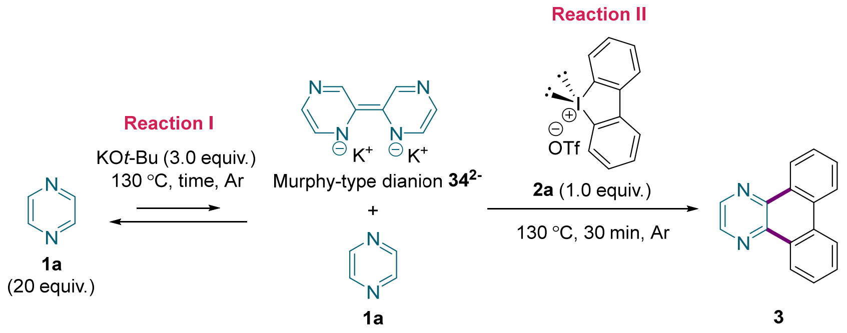

Entry	time (min)	yield (%)
1	2	43
2	5	36
3	15	22
4	30	2

Supplementary Figure 7 Synthesis of compound **3**; **1a**/KOt-Bu followed by the addition of **2a**

When the annulation was conducted in two steps, the yield of **3** dropped significantly and decreased with increasing reaction time of the first step (from 2 to 30 min). These results indicate that potassium *tert*-butoxide is consumed in the first step (formation of OEDs) and also needed in the second step.

Detailed reaction procedure (Reaction II)

In an Ar-filled glove box, a screw-cap 1-dram vial was charged with KO*t*-Bu (0.60 mmol, 3.0 equiv.) and pyrazine (4.0 mmol, 20 equiv.). After the reaction mixture was stirred at 130 °C for the specified time, it was allowed to cool to 70 °C. The vial was brought back into an Ar-filled glove box and cyclic diphenyliodonium salt **2a** (0.20 mmol, 1.0 equiv.) was added. After the mixture was stirred at 130 °C for 30 min, it was allowed to cool to RT and diluted with DCM (2 mL). After the sample was sonicated for 1 h at RT, it was directly purified by column chromatography to afford compound **3**.

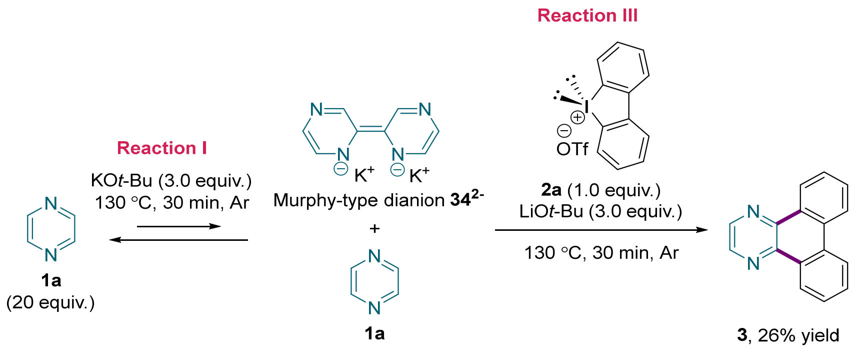

Supplementary Figure 8 Synthesis of compound **3**; **1a**/KO*t*-Bu followed by LiO*t*-Bu/**2a**

When lithium *tert*-butoxide was added along with **2a** in the second step of the two-step annulation experiment, the yield of **3** increased from 2 to 26 %. This result confirms that a base is required in the second step[15,17].

Detailed reaction procedure (Reaction III)

In an Ar-filled glove box, a screw-cap 1-dram vial was charged with KO*t*-Bu (0.60 mmol, 3.0 equiv.) and pyrazine (4.0 mmol, 20 equiv.). After the reaction mixture was stirred at 130 °C for 30 min, it was allowed to cool to 70 °C. The vial was brought back into an Ar-filled glove box and cyclic diphenyliodonium salt **2a** (0.20 mmol, 1.0 equiv.) and LiO*t*-Bu (0.60 mmol, 3.0 equiv.) were added. After the mixture was stirred at 130 °C for 30 min, it was allowed to cool to RT and diluted with DCM (2 mL). After the sample was sonicated for 1 h at RT, it was directly purified by column chromatography to afford compound **3** (12 mg, 26%).

1.11. EPR Spectroscopic Analysis

Sample Preparation. The reaction mixtures were prepared in an Ar atmosphere as described in Sections 1.6 and 1.10 and heated at 130 °C for 30 min, unless otherwise noted. In each case, the reaction mixture was allowed to cool to RT and the resulting green–black (reaction I) or brown–black (annulation) glassy solid was crushed and suspended in 1 mL of degassed and purified toluene (MBraun solvent purification system). The suspension was then transferred into an EPR tube in air, unless otherwise specified in the figure captions.

Measurements. Electron paramagnetic resonance (EPR) spectra were obtained on a Bruker EMXplus 9.5/12 spectrometer, equipped with an Oxford Instruments ESR900 cryostat, at 295 K and under nonsaturating conditions. Typical measurement parameters were an X-band microwave frequency of 9.38 GHz, a modulation frequency of 100 kHz, a modulation amplitude of 1 G, and a microwave power of 0.633 or $6.33 \cdot 10^{-3}$ mW. The spectra shown in the figures were acquired with 10 scans.

Description of the Spectra Shown in Supplementary Figures 9–17. The spectra are shown in first-derivative form, with the exception of those in Supplementary Figures 13 and 15, which are shown as absorption spectra. Intensities were compared based on absorption peak areas obtained by double-integration of the first-derivative spectra.

*Pyrazine-Derived Radical Anions (**1a**$^{\bullet-}$/**34**$^{\bullet-}$).* The intensity of the signal of the pyrazine-derived radical anions (**1a**$^{\bullet-}$/**34**$^{\bullet-}$) increased over a reaction time of 30 min at 130 °C as well as with temperature in the range of 80–130 °C (Supplementary Figures 9 and 10). Spectra of independent samples, with sample transfer conducted in either air or Ar, show only minor changes in signal intensity (Supplementary Figure 11). The spectra of samples that were heated at 130 °C for 30 min either once or twice are nearly identical (Supplementary Figure 12), indicating that the second heating phase is not detrimental to the radical species present. The reaction of the radical anions with **2a** resulted in a decrease of the signal intensity by about two-thirds (Fig. 4b and Supplementary Figure 13), consistent with the consumption of 2 equiv. of **1a**$^{\bullet-}$/**34**$^{\bullet-}$, one each for reaction with **2a** and for subsequent termination of other radical species formed. For the reaction of the radical anions with **2a** and LiO*t*-Bu, the decrease of intensity is smaller, presumably, because the base facilitates product formation and thereby lowers the lifetime of radical intermediates that could consume the radical anions **1a**$^{\bullet-}$/**34**$^{\bullet-}$.

Annulation. The spectra from Fig. 4c are shown with normalised derivative peak heights in Supplementary Figure 14 and as absorption spectra in Supplementary Figure 15. Exposure of the reaction mixture to air during sample transfer caused significant changes in the intensity and shape of the signal (Supplementary Figure 16). Spectra of samples with different reaction times revealed that the signal persists throughout the reaction (Supplementary Figure 17).

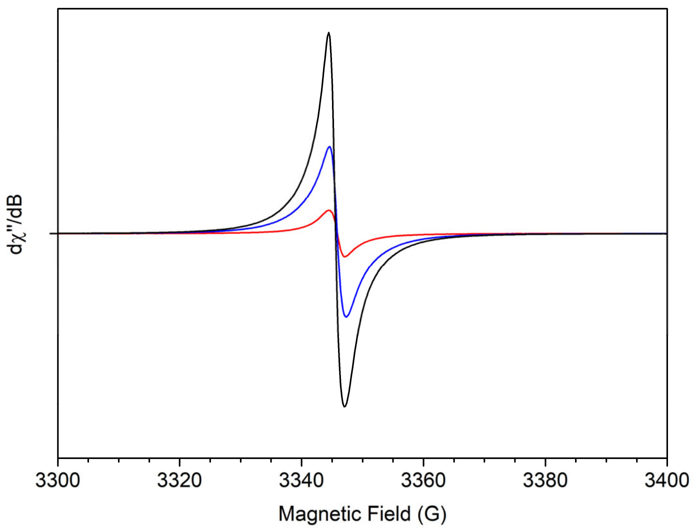

Supplementary Figure 9 EPR spectra (X-band, 295 K) of samples from the reaction of 4.0 mmol of pyrazine with 0.60 mmol of KO*t*-Bu at 130 °C with a reaction time of 5 min (solid red line), 15 min (solid blue line) or 30 min (solid black line).

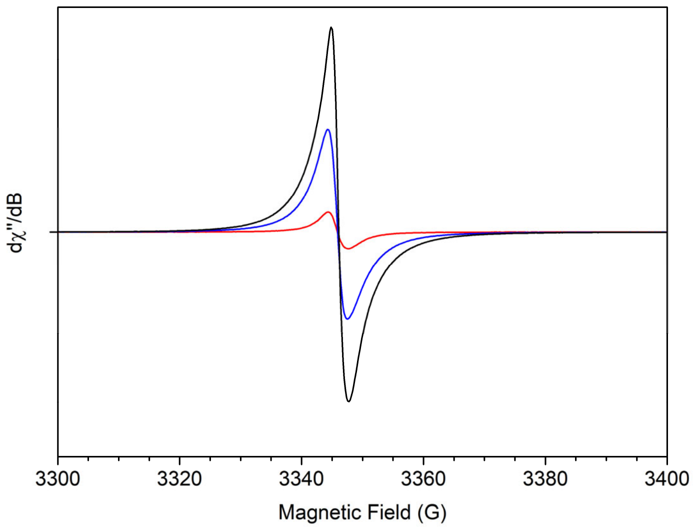

Supplementary Figure 10 EPR spectra (X-band, 295 K) of samples from the reaction of 4.0 mmol of pyrazine with 0.60 mmol of KO*t*-Bu at 80 °C (solid red line), 100 °C (solid blue line) or 130 °C (solid black line) with a reaction time of 30 min.

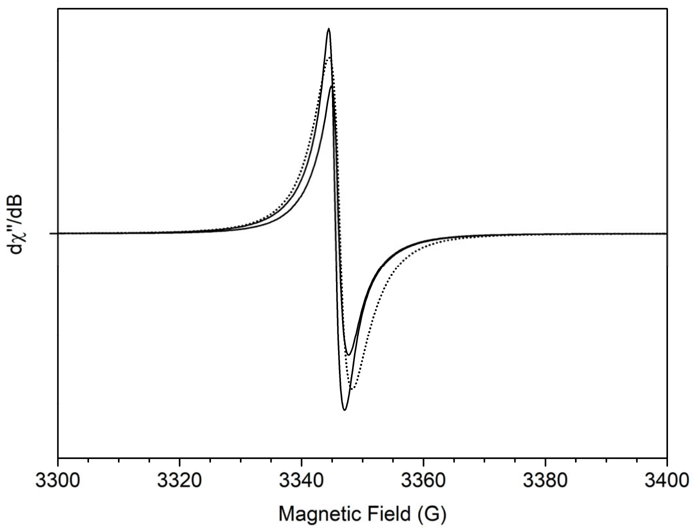

Supplementary Figure 11 EPR spectra (X-band, 295 K) of samples from the reaction of 4.0 mmol of pyrazine with 0.60 mmol of KO*t*-Bu, with sample transfer conducted in air (two independent samples, solid black lines) or in an Ar atmosphere (dotted black line).

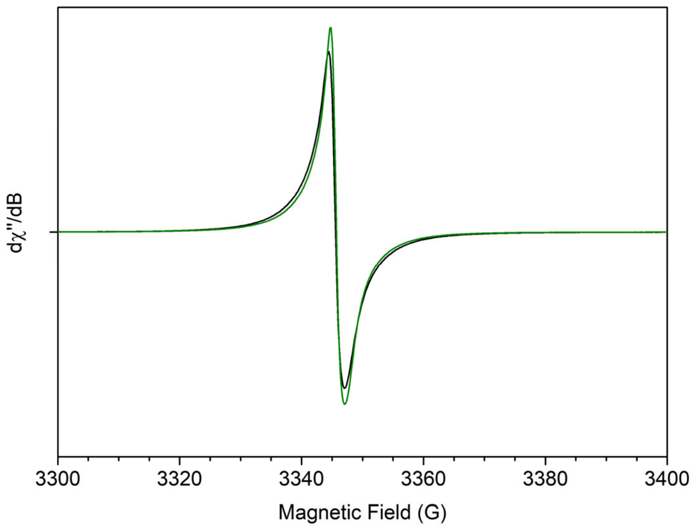

Supplementary Figure 12 EPR spectra (X-band, 295 K) of samples from the reaction of 4.0 mmol of pyrazine with 0.60 mmol of KO*t*-Bu at 130 °C with a reaction time of 30 min (solid black line) or two heating phases of 30 min each (solid green line).

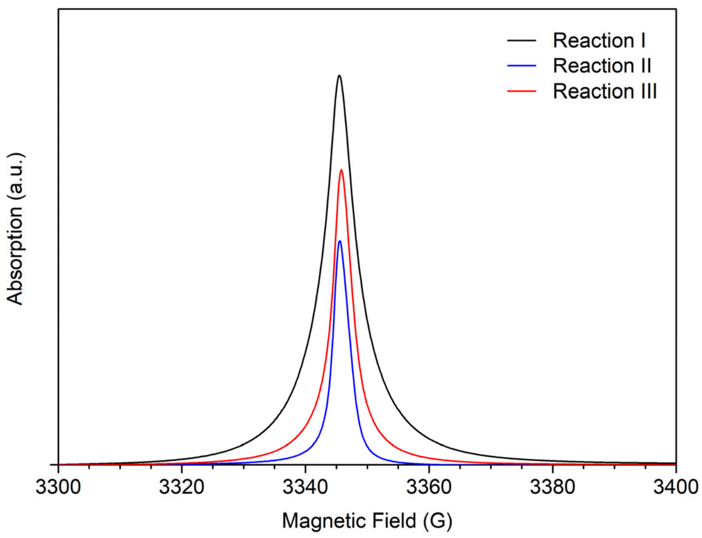

Supplementary Figure 13 EPR spectra from Fig. 4b shown as absorption spectra. EPR spectra (X-band, 295 K) of samples from the reaction of 4.0 mmol of pyrazine with 0.60 mmol of KO*t*-Bu (reaction I; solid black line), from the two-step reaction of pyrazine with KO*t*-Bu and then with 0.20 mmol of **2a** (reaction II; solid blue line), and from the two-step reaction of pyrazine with KO*t*-Bu and then with 0.20 mmol of **2a** and 0.60 mmol of LiO*t*-Bu (reaction III; solid red line).

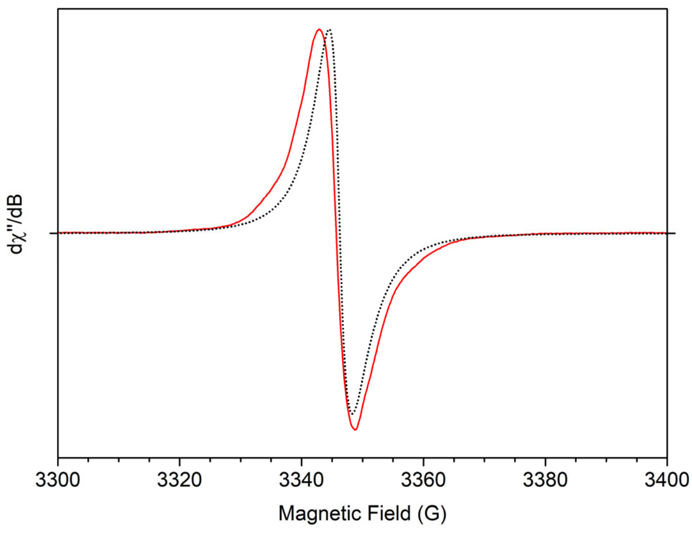

Supplementary Figure 14 EPR spectra from Fig. 4c shown with normalised derivative peak heights. EPR spectra (X-band, 295 K) of samples from the reaction of 4.0 mmol of pyrazine with 0.60 mmol of KO*t*-Bu (reaction I; dotted black line) and from the annulation of 4.0 mmol of pyrazine with 0.60 mmol of KO*t*-Bu and 0.20 mmol of **2a** (solid red line). The sample transfer was conducted in an Ar atmosphere in both cases. The spectrum of the annulation was acquired using a microwave power one hundred times that used for the spectrum of the pyrazine–KO*t*-Bu reaction.

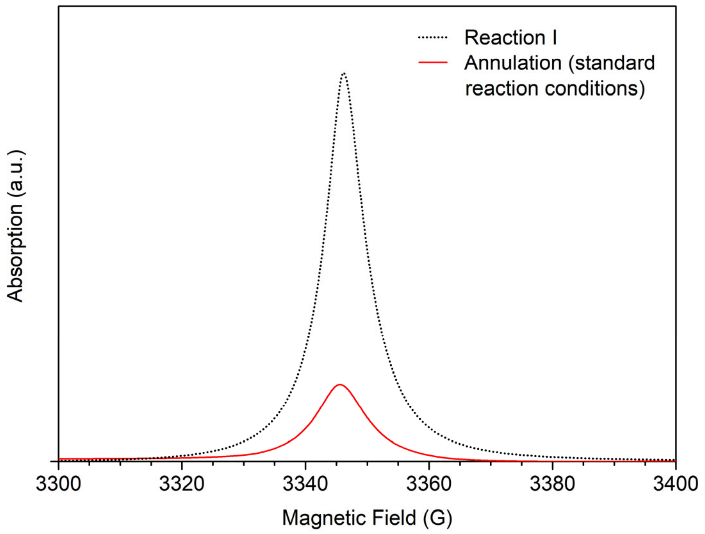

Supplementary Figure 15 EPR spectra from Fig. 4c shown as absorption spectra.

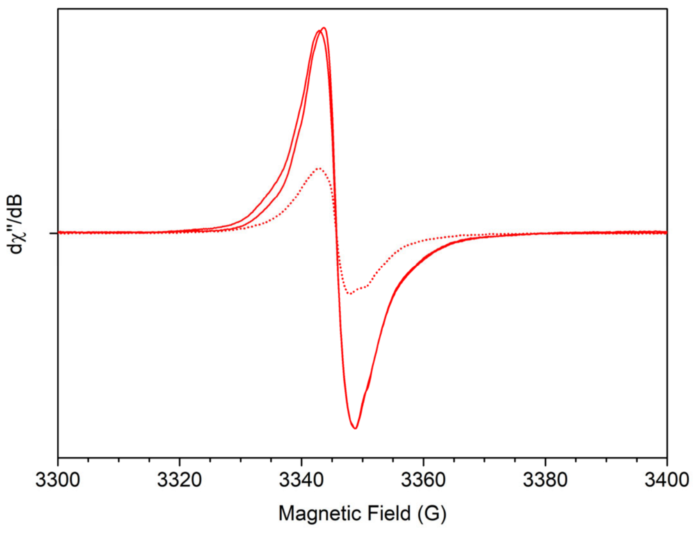

Supplementary Figure 16 EPR spectra (X-band, 295 K) of samples from the annulation of 4.0 mmol of pyrazine with 0.60 mmol of KO*t*-Bu and 0.20 mmol of **2a**, with sample transfer conducted in an Ar atmosphere (two independent samples, solid red lines) or in air (dotted red line).

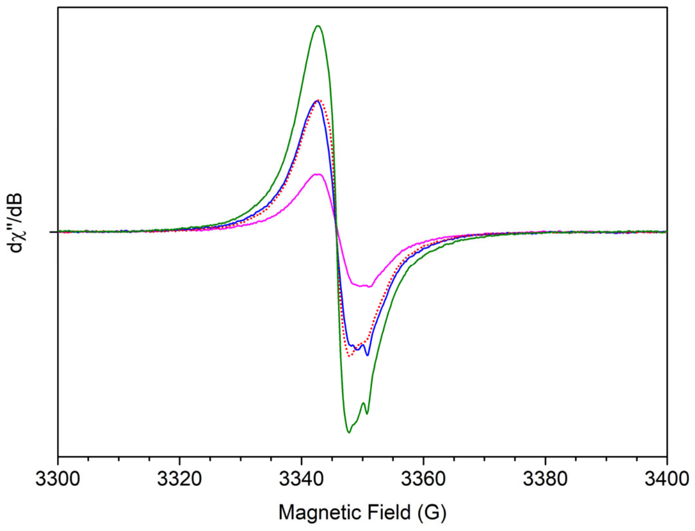

Supplementary Figure 17 EPR spectra (X-band, 295 K) of samples from the annulation of 4.0 mmol of pyrazine with 0.60 mmol of KO*t*-Bu and 0.20 mmol of **2a** at 130 °C with a reaction time of 5 min (solid magenta line), 10 min (solid blue line), 30 min (dotted red line), or 60 min (solid green line).

1.12. Chemical Inhibition Experiment with TEMPO

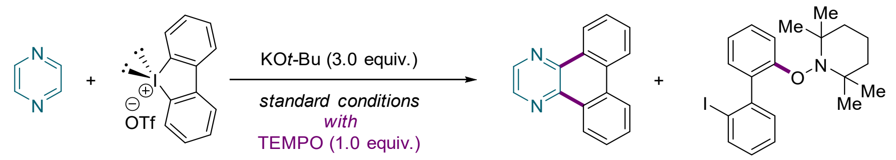

1a (20 equiv.) **2a** (1.0 equiv.) **3** trace **35** detected by high-resolution ESI MS

Supplementary Figure 18 Trapping with TEMPO

When the annulation was performed in the presence of 2,2,6,6-tetramethyl-1-piperidinyloxy (TEMPO) as a radical trapping agent, the formation of **3** was inhibited, indicating that this reaction proceeds through a radical-based mechanism. In addition, a species consistent with the TEMPO adduct of an iodobiphenyl radical (**35**) was detected by high-resolution ESI MS, suggesting that an iodanyl radical is involved in the reaction pathway.

Detailed reaction procedure

In an Ar-filled glove box, a screw-cap 1-dram vial was charged with cyclic diphenyliodonium salt **2a** (0.20 mmol, 1.0 equiv.), KO*t*-Bu (0.60 mmol, 3.0 equiv.), 2,2,6,6-tetramethyl-1-piperidinyloxy (0.20 mmol, 1.0 equiv.) and pyrazine (4.0 mmol, 20 equiv.). After the reaction mixture was stirred at 130 °C for 30 min, it was allowed to cool to RT and diluted with DCM (2 mL). After the sample was sonicated for 1 h at RT, it was analyzed by TLC and high-resolution ESI MS.

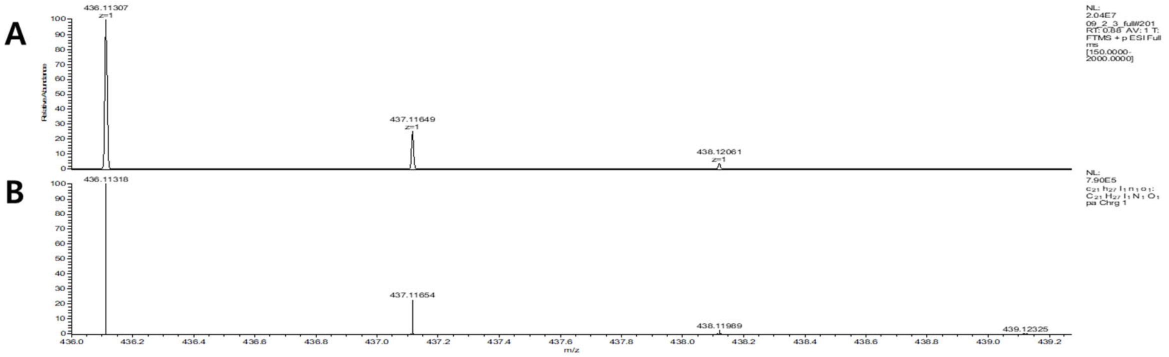

Supplementary Figure 19 (**A**) measured HRMS isotope distribution of compound **35** and (**B**) its calculated HRMS isotope distribution.

1.13. Electrochemical Reduction

1.13.1. Chronopotentiometry experiment

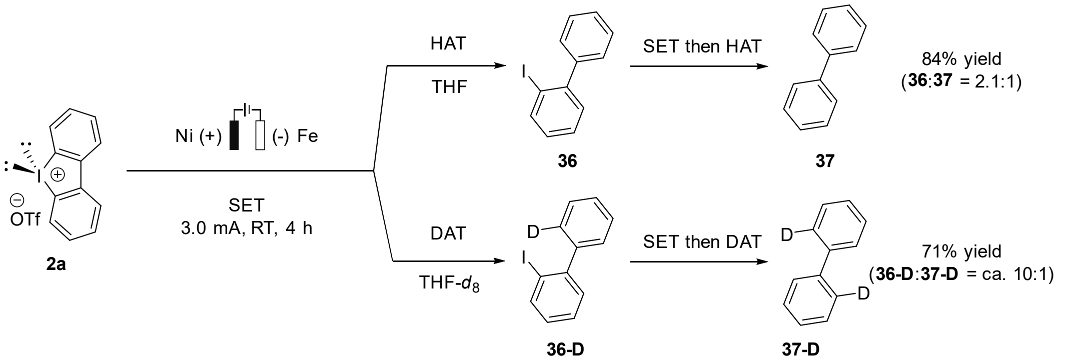

Supplementary Figure 20 Electrochemical reactions

The electrochemical reduction to demonstrate the SET process on iodonium salt **2a** afforded **36** and **37** through hydrogen atom transfer (HAT). A deuterium-labelling experiment confirmed THF as the H atom source.

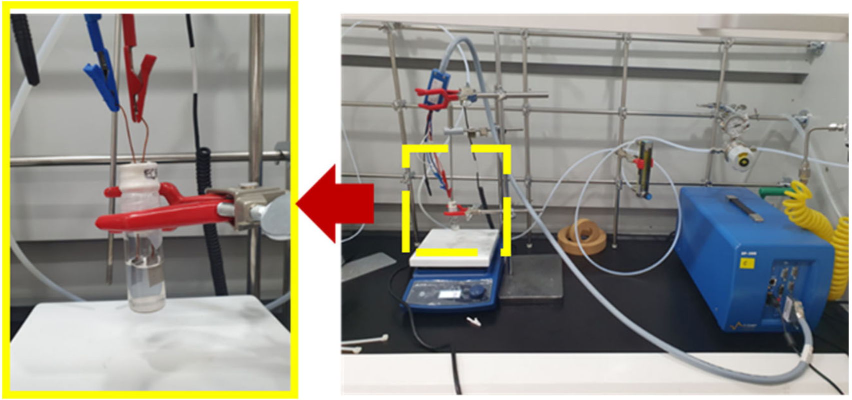

Supplementary Figure 21 Electrochemical setup

Detailed reaction procedure I (HAT)

In an Ar-filled glove box, a solution of cyclic diphenyliodonium salt **2a** (0.40 mmol, 1.0 equiv.) in *n*-Bu_4NBF_4/THF (0.30 M, 5 mL) was added to a 2.5-dram vial, which was capped with a rubber septum equipped with an electrode system. The undivided cell equipped with anode (Fe metal) and cathode (Ni foam) was electrolysed at a constant current of 3.0 mA for 4 h. The rubber septum was removed, and the electrodes were sonicated for 5 min. The reaction mixture was directly purified by column chromatography to afford a mixture of **36** and **37** (2.1:1 ratio based on ^{1}H NMR analysis; see below).

Yellow oil (80 mg, 84%); TLC R_f = 0.5 (*n*-hexane); **^{1}H NMR** (400 MHz, $CDCl_3$) δ 7.98 (dd, *J* = 7.9,

1.3 Hz), 7.62 (dd, J = 8.3, 1.3 Hz), 7.49 – 7.30 (m), 7.08 – 7.02 (m).

The NMR spectrum of the mixture of **36** and **37** is consistent with previously reported literature data[18,19].

Calculation of the ratio of compound **36** and compound **37:** A product ratio of 2.1:1 was obtained from ^{1}H NMR analysis, based on the peak at δ 7.98 (1H) for **36** and the peak at δ 7.62 (4H) for **37**.

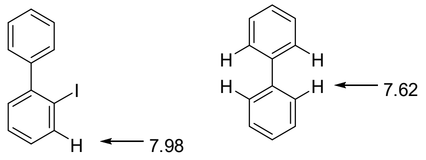

compound **36** compound **37**

(δ 7.98) integral of 1H$_{(\text{compound } \mathbf{36})}$ = 1.00

(δ 7.62) integral of 4H$_{(\text{compound } \mathbf{37})}$ = 1.90

⇨ compound **36**: compound **37** ≈ 2.1:1

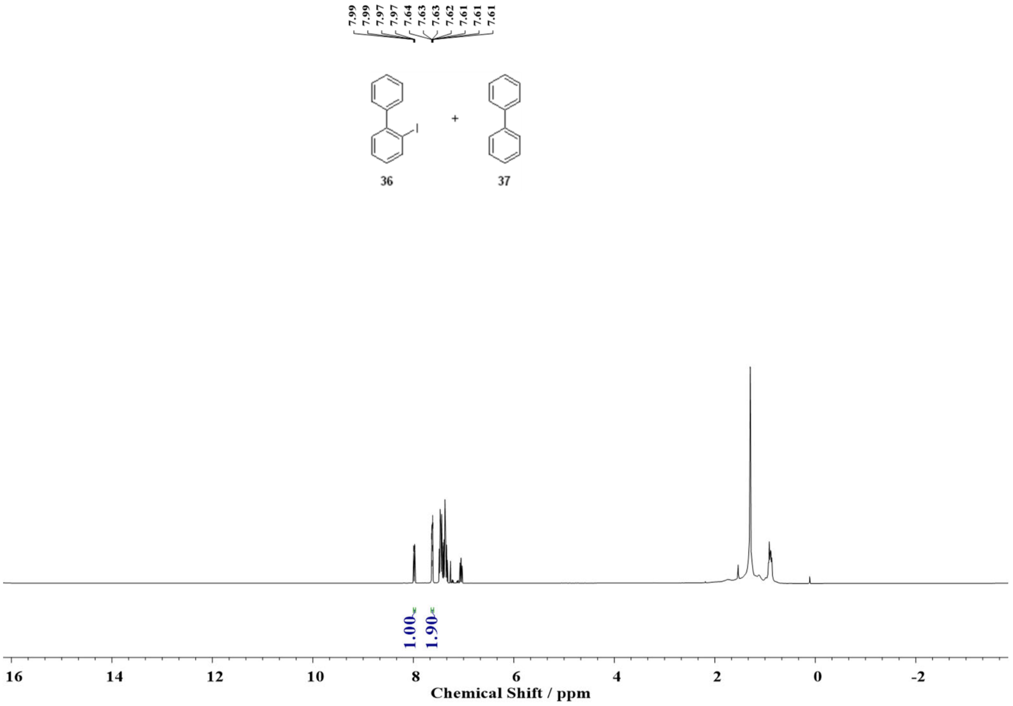

Supplementary Figure 22 ^{1}H NMR (400 MHz, $CDCl_3$) of the mixture of compounds **36** and **37**

Detailed reaction procedure II (DAT)

In an Ar-filled glove box, a solution of cyclic diphenyliodonium salt **2a** (0.40 mmol, 1.0 equiv.) in *n*-Bu_4NBF_4/THF-d_8 (0.30 M, 5 mL) was added to a 2.5-dram vial, which was capped with a rubber septum

equipped with an electrode system. The undivided cell equipped with anode (Fe metal) and cathode (Ni foam) was electrolysed at a constant current of 3.0 mA for 4 h. The rubber septum was removed, and the electrodes were sonicated for 5 min. The reaction mixture was directly purified by column chromatography to afford a mixture of **36-D** and **37-D** (ca. 10:1 ratio based on ^{1}H NMR analysis; see below).

Yellow oil (78 mg, 72%); TLC R_f = 0.5 (*n*-hexane); **^{1}H NMR** (400 MHz, $CDCl_3$) δ 7.98 (dd, *J* = 7.9, 1.3 Hz), 7.62 (dd, *J* = 8.3, 1.3 Hz), 7.49 – 7.30 (m), 7.08 – 7.02 (m).

A product ratio of ca. 10:1 was obtained from ^{1}H NMR analysis, based on the peak at δ 7.98 (1H) for **36-D** and the peak at δ 7.62 (2H) for **37-D**.

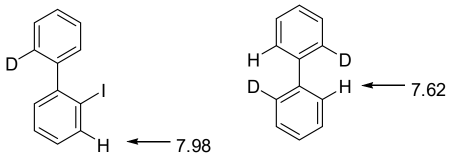

compound **36-D** compound **37-D**

(δ 7.98) integral of 1H$_{(\text{compound } \mathbf{36\text{-}D})}$ = 1.00

(δ 7.62) integral of 2H$_{(\text{compound } \mathbf{37\text{-}D})}$ = 0.20

⇨ compound **36-D**: compound **37-D** ≈ 10:1

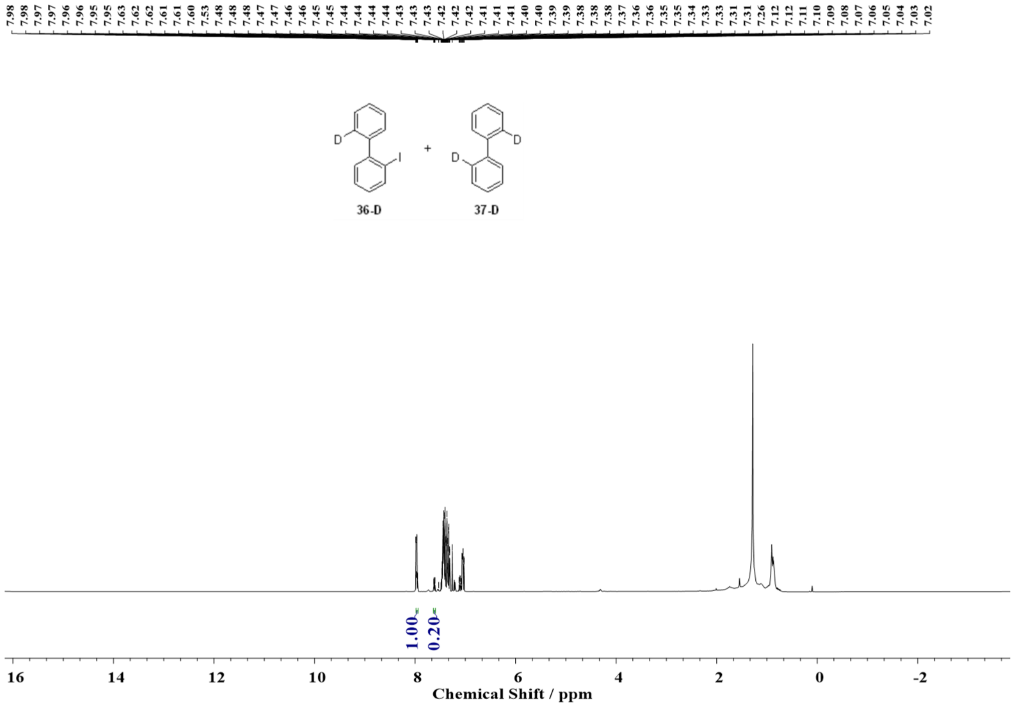

Supplementary Figure 23 ^{1}H NMR (400 MHz, $CDCl_3$) of the mixture of compounds **36-D** and **37-D**

1.13.2. Chronoamperometry experiment

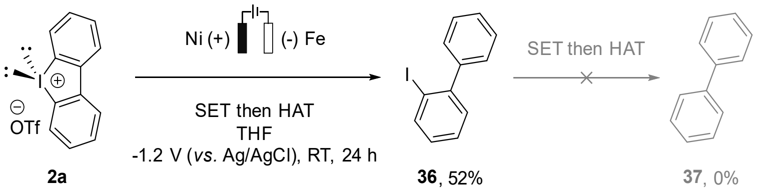

Supplementary Figure 24 Electrochemical experiment to afford compound **36**

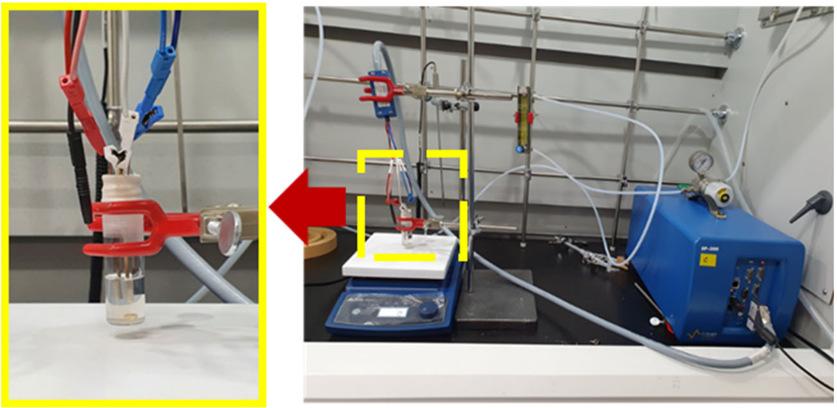

Supplementary Figure 25 Electrochemical set up

Detailed reaction procedure

In an Ar-filled glove box, a solution of cyclic diphenyliodonium salt **2a** (0.40 mmol, 1.0 equiv.) in *n*-Bu_4NBF_4/THF (0.30 M, 5 mL) was added to a 2.5-dram vial, which was capped with a rubber septum equipped with an electrode system. The undivided three-electrode cell equipped with anode (Fe metal), cathode (Ni foam), and reference electrode (Ag/AgCl) was electrolysed at a constant potential of -1.2 V (*vs.* Ag/AgCl) for 24 h. The rubber septum was removed, and the electrodes were sonicated for 5 min. The reaction mixture was directly purified by column chromatography to afford **36**.

Yellow oil (58 mg, 52%); TLC R_f = 0.5 (*n*-hexane); **^{1}H NMR** (400 MHz, $CDCl_3$) δ 7.98 (dd, *J* = 8.0, 1.3 Hz, 1H), 7.48 – 7.30 (m, 7H), 7.07 – 7.01 (m, 1H).

The NMR spectrum of **36** matched previously reported literature data[18].

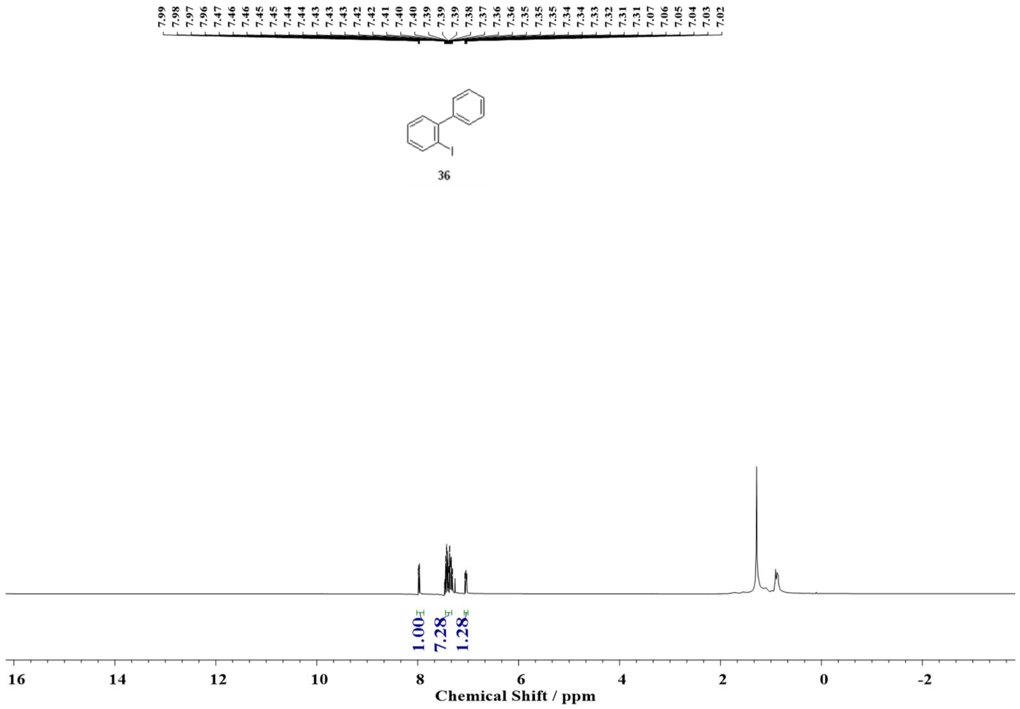

Supplementary Figure 26 ^{1}H NMR (400 MHz, $CDCl_3$) of compound **36**

1.14. Competition Experiment Between 2a and PhI

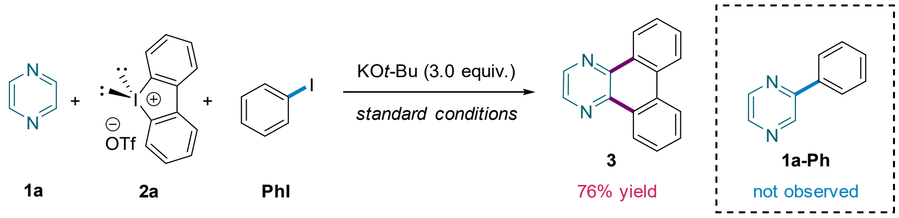

A competition experiment between cyclic diphenyliodonium salt **2a** and iodobenzene revealed that the hypervalent iodine(III) compound is inherently more reactive, which is in accordance with the reduction potentials of cyclic diphenyliodonium salt **2a** and iodobenzene (Section 1.15).

Detailed reaction procedure

In an Ar-filled glove box, a screw-cap 1-dram vial was charged with cyclic diaryliodonium salt **2a** (0.20 mmol, 1.0 equiv.), iodobenzene (0.20 mmol, 1.0 equiv.), KO*t*-Bu (0.60 mmol, 3.0 equiv.) and pyrazine (4.0 mmol, 20 equiv.). After the reaction mixture was stirred at 130 °C for 30 min, it was allowed to cool to RT and diluted with DCM (2 mL). After the sample was sonicated for 1 h at RT, it was analyzed by TLC and directly purified by column chromatography to afford compound **3**.

1.15. Cyclic Voltammetry Studies

1.15.1. General information

Ferrocene, PhI, 2-iodobiphenyl, pyrazine, *n*-Bu_4NBF_4, and anhydrous DMF were purchased and used as supplied. Cyclic diphenyliodonium salt **2a** and bipyrazine **34** were prepared as described in Sections 1.3 and 1.9.

1.15.2. Cyclic voltammograms of ferrocene and cyclic diphenyliodonium salt (2a)

The three-electrode beaker cell was made of a platinum plate working electrode (area = 1.2 cm^2), a saturated calomel electrode (SCE) as reference electrode, and a platinum wire counter electrode. The solution was prepared at 4.0 mM concentration in 0.30 M *n*-Bu_4NBF_4/DMF using ferrocene as an external standard. Cyclic voltammetry of ferrocene and **2a** was performed at a fixed scan rate of 0.05 V s^{-1}.

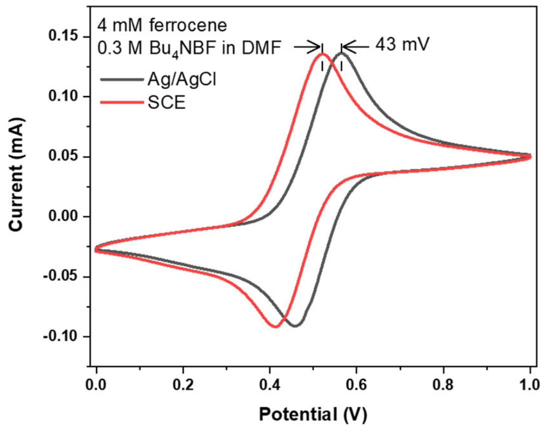

Supplementary Figure 27 Cyclic voltammogram (CV) of ferrocene using an SCE or Ag/AgCl reference electrode. E(SCE) = E(Ag/AgCl) – 0.043 V.

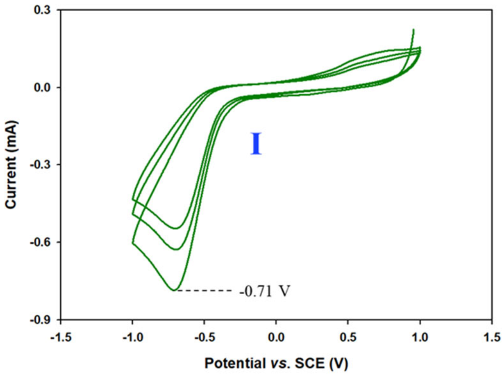

Supplementary Figure 28 CV of **2a**.

1.15.3. CV of PhI

The three-electrode beaker cell consisted of a glassy carbon working electrode (diameter = 6.0 mm), an Ag/AgCl (Sat'd KCl) or SCE reference electrode, and a platinum wire counter electrode. The solution was prepared at 4.0 mM concentration in 0.30 M *n*-Bu_4NBF_4/DMF using ferrocene as an external standard for the SCE-scale conversion. Cyclic voltammetry of PhI was performed at a fixed scan rate of 0.50 V s^{-1}.

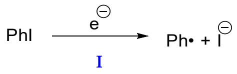

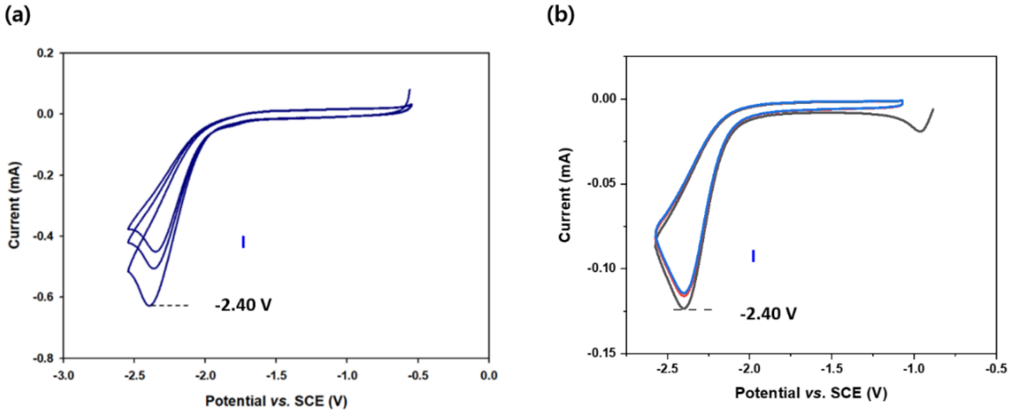

Supplementary Figure 29 CV of PhI. (a) Ag/AgCl reference electrode, potentials converted to the SCE scale using ferrocene as an external standard. (b) SCE reference electrode.

1.15.4. CV of 2-iodobiphenyl

The three-electrode beaker cell consisted of a glassy carbon working electrode (diameter = 6.0 mm), an Ag/AgCl (Sat'd KCl) or SCE reference electrode, and a platinum wire counter electrode. The solution was prepared at 4.0 mM concentration in 0.30 M *n*-Bu_4NBF_4/DMF using ferrocene as an external standard for the SCE-scale conversion. Cyclic voltammetry of 2-iodobiphenyl was performed at a fixed scan rate of 0.50 V s^{-1}.

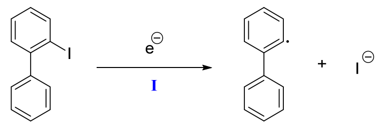

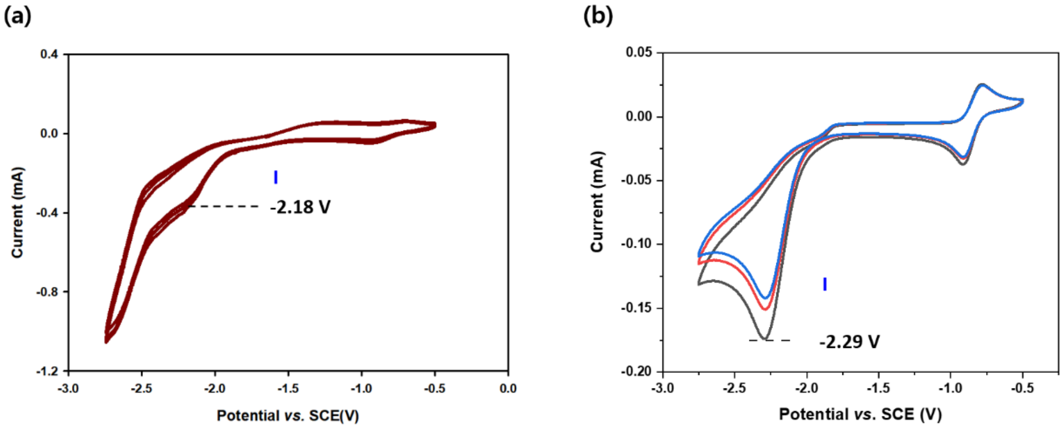

Supplementary Figure 30 CV of 2-iodobiphenyl. (a) Ag/AgCl reference electrode, potentials converted to the SCE scale using ferrocene as an external standard. (b) SCE reference electrode.

1.15.5. CVs of pyrazine (1a) and 2,2′-bipyrazine (34)

The three-electrode beaker cell consisted of a glassy carbon working electrode (diameter = 6.0 mm), an Hg/HgO (20% KOH) reference electrode, and a platinum wire counter electrode. The solution was prepared at 4.0 mM concentration in 0.30 M *n*-Bu_4NBF_4/DMF using ferrocene as an external standard for the conversion of potentials to the SCE scale. Cyclic voltammetry of **1a** and **34** was performed at a fixed scan rate of 0.05 V s^{-1}.

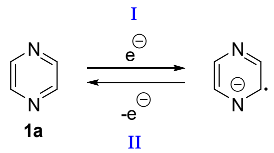

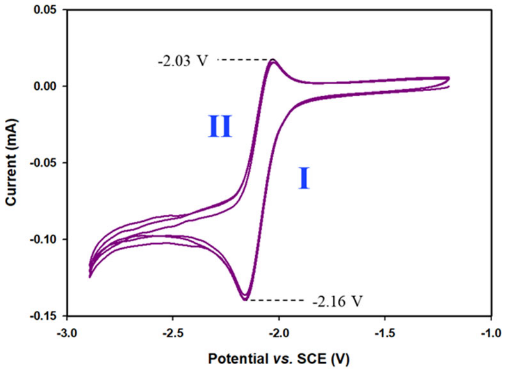

Supplementary Figure 31 CV of **1a**.

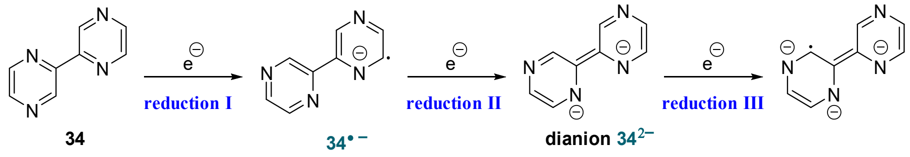

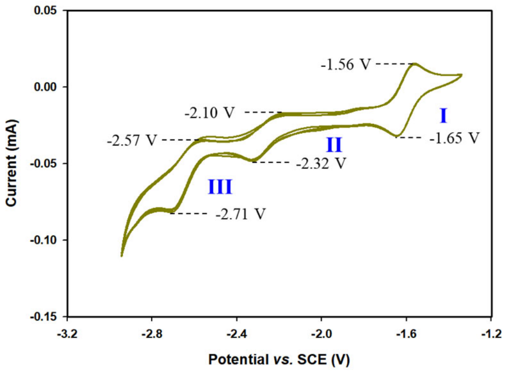

Supplementary Figure 32 CV of **34**.

1.16. Deuterium-Labelling Experiments

1.16.1. Annulative π-extension with deuterium-labelled pyrazine-d_4

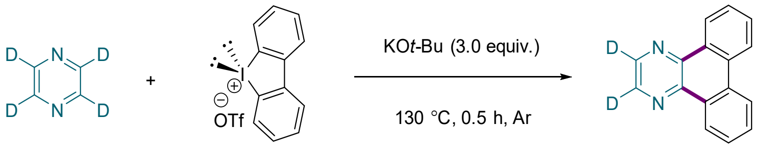

1a-D **2a** **3-D** 80%

Supplementary Figure 33 Synthesis of compound **3-D**

Annulative π-extension of pyrazine-d_4 (**1a-D**) under the standard reaction conditions yielded the desired APEX product **3-D** in 80% yield.

Detailed reaction procedure

In an Ar-filled glove box, a screw-cap 1-dram vial was charged with cyclic diaryliodonium salt **2a** (0.20 mmol, 1.0 equiv.), KO*t*-Bu (0.60 mmol, 3.0 equiv.) and pyrazine-d_4 (98 atom % D, 4.0 mmol, 20 equiv.). After the reaction mixture was stirred at 130 °C for 30 min, it was cooled to RT and diluted with DCM (2 mL). After sonicating the sample for 1 h at RT, the reaction mixture was directly purified by column chromatography to afford compound **3-D**.

Yellow solid (37 mg, 80%); TLC R_f = 0.5 (*n*-hexane:Et_2O = 20:1); **^{1}H NMR** (400 MHz, $CDCl_3$) δ 9.24 (dd, *J* = 8.0, 1.3 Hz, 2H), 8.63 (dd, *J* = 8.0, 1.3 Hz, 2H), 7.77 (m, 4H).

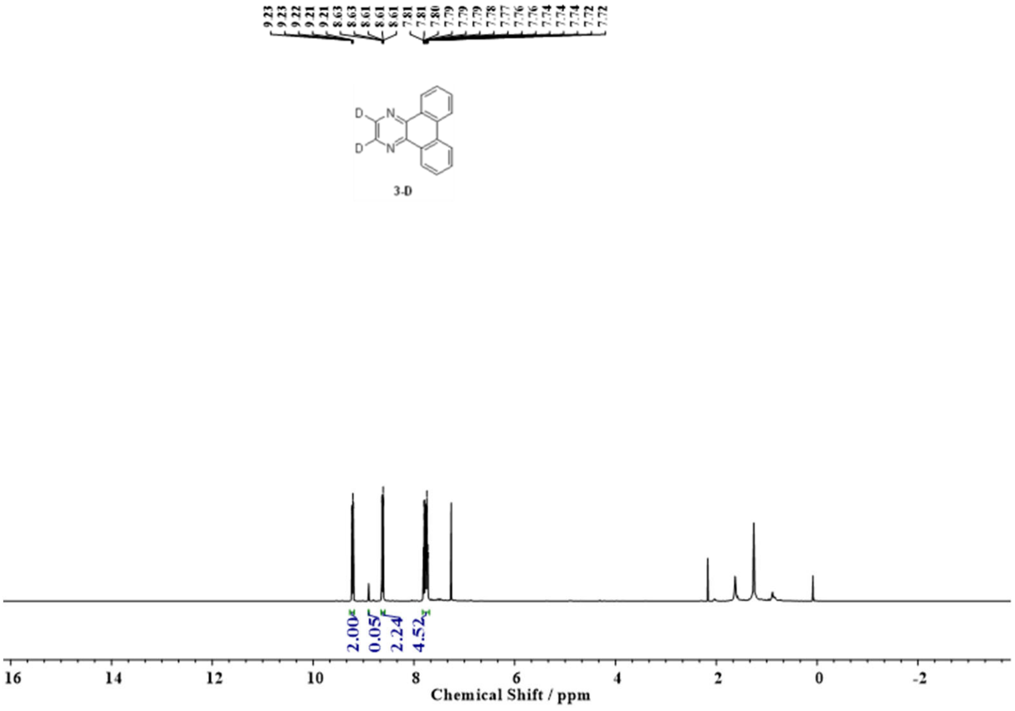

Supplementary Figure 34 ^{1}H NMR (400 MHz, $CDCl_3$) of compound **3-D**

1.16.2. Competition experiment between pyrazine and pyrazine-d_4

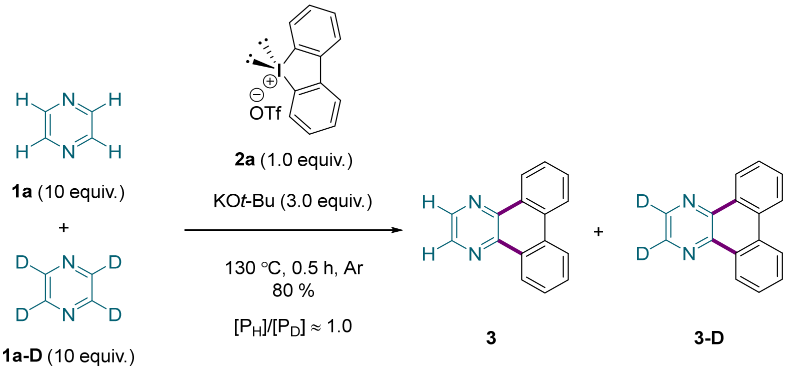

Supplementary Figure 35 Competition experiment to give **3** and **3-D**

A competition experiment between pyrazine (**1a**) and pyrazine-d_4 (**1a-D**) was investigated. The kinetic isotope effect (KIE) determined from this experiment reveals that C–H bond activation is not involved in the rate-determining-step (RDS).

Detailed reaction procedure

In an Ar-filled glove box, a screw-cap 1-dram vial was charged with cyclic diaryliodonium salt **2a** (0.20 mmol, 1.0 equiv.), KO*t*-Bu (0.60 mmol, 3.0 equiv.), pyrazine (2.0 mmol, 10 equiv.) and pyrazine-d_4 (98 atom % D, 2.0 mmol, 10 equiv.). After the reaction mixture was stirred at 130 °C for 30 min, it was cooled to RT and diluted with DCM (2 mL). After sonicating the sample for 1 h at RT, the reaction mixture was directly purified by column chromatography to afford a mixture of **3** and **3-D**.

The product ratio was obtained from ^{1}H NMR analysis based on the distinct peak at δ 8.90 (2H) for **3**.

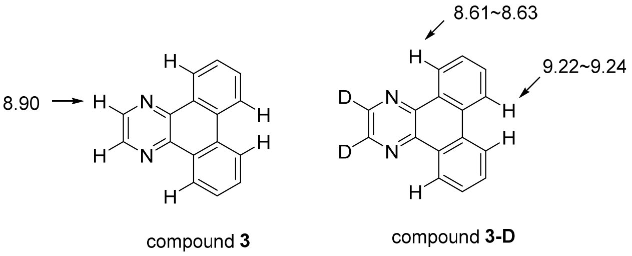

Calculation of the ratio of compound **3** and compound **3-D**:

(δ 8.90) integral of $2H_{(\text{compound }\mathbf{3})}$ = 1.02

(δ 8.61) integral of $2H_{(\text{compound }\mathbf{3})}$ + $2H_{(\text{compound }\mathbf{3\text{-}D})}$ = 2.12

(δ 9.22) integral of $2H_{(\text{compound }\mathbf{3})}$ + $2H_{(\text{compound }\mathbf{3\text{-}D})}$ = 1.93

⇨ compound **3** : compound **3-D** ≈ 1:1

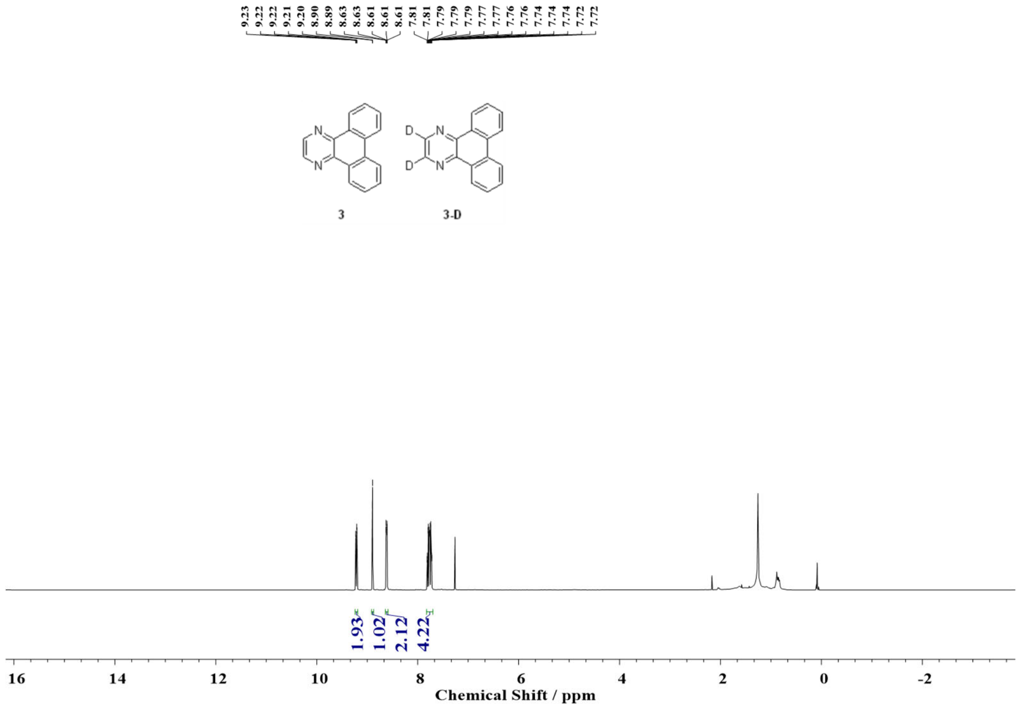

Supplementary Figure 36 ^{1}H NMR (400 MHz, $CDCl_3$) of the mixture of compounds **3** and **3-D**

1.17. Evidence of a Reaction Intermediate

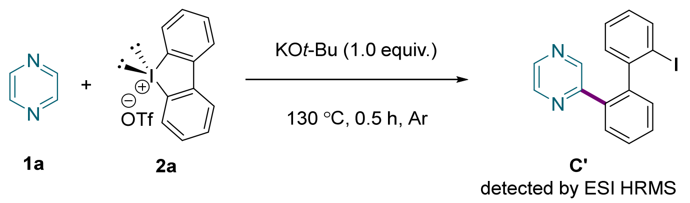

Supplementary Figure 37 Formation of byproduct **C'**

The monoarylated byproduct **C'** was detected by analysis of the reaction mixture by high-resolution ESI MS. This is the oxidized form of intermediate **C** in the proposed mechanism (Fig. 6).

Detailed reaction procedure

In an Ar-filled glove box, a screw-cap 1-dram vial was charged with cyclic diaryliodonium salt **2a** (0.20 mmol, 1.0 equiv.), KO*t*-Bu (0.20 mmol, 1.0 equiv.) and pyrazine (4.0 mmol, 20 equiv.). After the reaction mixture was stirred at 130 °C for 30 min, it was cooled to RT and diluted with DCM (2 mL). After sonicating for 1 h at RT, the reaction mixture was directly analyzed by high-resolution ESI MS.

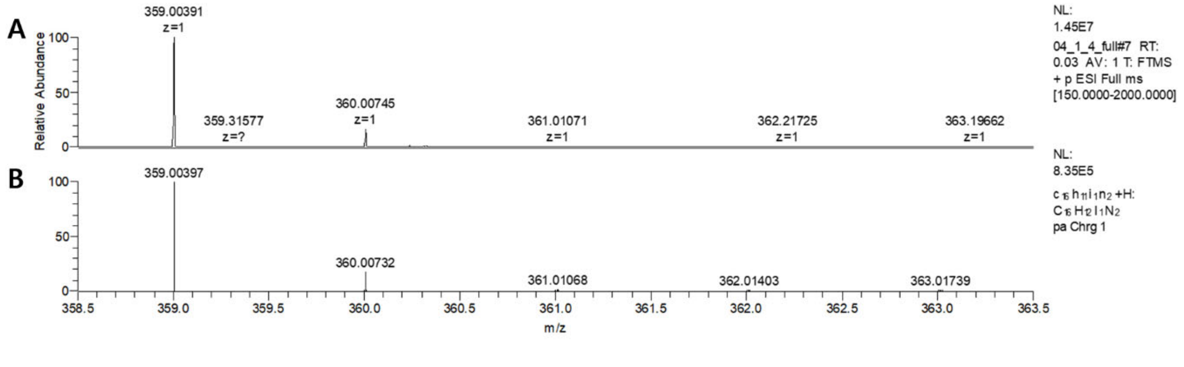

Supplementary Figure 38 (**A**) measured HRMS isotope distribution of byproduct **C'** and (**B**) its calculated HRMS isotope distribution.

1.18. X-Ray Crystallographic Data of 13, 14, and 23'

X-ray diffraction data of single crystal of compound **13** were obtained at 220 K with monochromator (λ = 0.65 Å) synchrotron radiation source in Pohang Accelerator Laboratory (PAL) 2D beamline, Korea. X-ray diffraction data of single crystals of compounds **14** and **23'** were obtained at 173 K with Mo Kα radiation source using a Rigaku R-Axis Rapid II. Crystal structures of compounds **13, 14, 23'** were solved by the direct method and refined by full-matrix least-squares calculations using SHELXTL program package[20]. Thermal ellipsoids were shown at 70% probability. The data can be obtained free of charge from the Cambridge Crystallographic Data Centre: CCDC 2047992 (compound **13**), 2047991 (compound **14**), and CCDC 2116428 (compound **23'**).

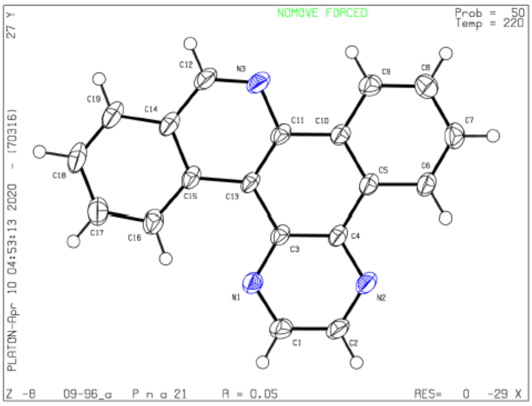

Supplementary Table 7 Crystal data and structure refinement for **13**

	**13**	
Molecular formula	$C_{19}H_{11}N_3$	
Temperature	220(2)K	
Crystal system	Orthorhombic	
Space group	*Pna*2*₁*	
Unit cell dimensions	a=11.516(2) Å	α=90°
	b=26.123(5) Å	β=90°
	c=4.3240(9) Å	γ=90°
V (Å^3)	1300.8(5) Å^3	
Z	4	
ρ_{calc} (g·cm^{-3})	1.436	
μ (mm^{-1})	0.087	
R_1, I > 2σ(I)	0.0507	
wR_2, $I > 2\sigma(I)$	0.1347	

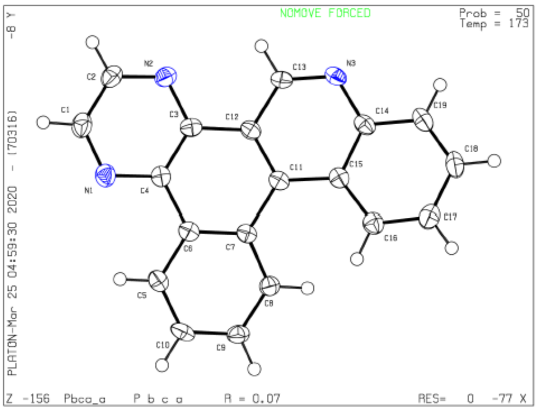

Supplementary Table 8 Crystal data and structure refinement for **14**

	**14**	
Molecular formula	$C_{19}H_{11}N_3$	
Temperature	173(2)K	
Crystal system	Orthorhombic	
Space group	*Pbca*	
Unit cell dimensions	a=17.776(4) Å	α=90°
	b=7.3553(15) Å	β=90°
	c=19.720(4) Å	γ=90°
V (Å^3)	2578.4(9) Å^3	
Z	8	
ρ_{calc} (g·cm^{-3})	1.449	
μ (mm^{-1})	0.088	
R_1, I > 2σ(I)	0.0739	
$wR_2, I > 2\sigma(I)$	0.1543	

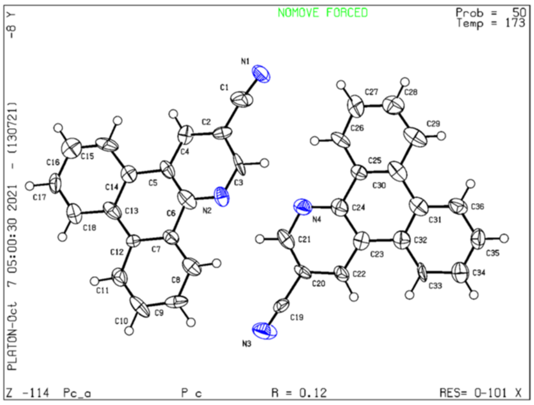

Supplementary Table 9 Crystal data and structure refinement for **23'**

	**23'**	
Molecular formula	$C_{18}H_{10}N_2$	
Temperature	173(2)K	
Crystal system	Monoclinic	
Space group	*Pc*	
Unit cell dimensions	a=10.373(2) Å	α=90°
	b=3.7935(8) Å	β=93.33(3)°
	c=30.434(6) Å	γ=90°
V (Å^3)	1195.5(4) Å^3	
Z	4	
ρ_{calc} (g·cm^{-3})	1.413	
μ (mm^{-1})	0.084	
R_1, I > 2σ(I)	0.1250	
wR_2, $I > 2\sigma(I)$	0.3406	

1.19. Summary of the Mechanistic Studies

Supplementary Table 10 Experimental evidences supporting BHAS mechanism

Experimental tools	Experimental findings
EPR spectroscopy<br>(Main text, Fig. 4)<br>(SI, Section 1.11)	The occurrence of organic radical species was unambiguously confirmed by EPR spectroscopy in combination with control experiments. In addition, compound **34** was isolated through oxidative workup of dianion $\mathbf{34}^{2-}$ with I_2.
Radical inhibition<br>(Main text, Fig. 5a)<br>(SI, Section 1.12)	Radical trapping experiment has been conducted; TEMPO completely shut down the reaction, and the TEMPO-adduct of an iodobiphenyl radical was observed.
Electrochemical reduction<br>(Main text, Fig. 5b)<br>(SI, Section 1.13)	Cathodic reduction of cyclic diphenyl iodonium salt (**2a**) provided the products **36** or **37** depending on chronopotentiometry or chronoamperometry modes through SET and successive HAT processes.
Competition experiments & CV measurements<br>(Main text, Fig. 5c)<br>(SI, Sections 1.14 and 1.15)	The competition reaction between **2a** and phenyl iodide gives rise to the selective formation of the annulated product **3** without the presence of mono-arylated product **1a-Ph**. The highly negative redox potential of dianion $\mathbf{34}^{2-}$ also clearly implicates that facile electron transfer to electrophilic **2a**.
KIE reaction<br>(Main text, Fig. 5d)<br>(SI, Section 1.16)	Our BHAS approach involving deprotonation event to give radical anion species reveals $[P_H]/[P_D] \approx 1$, indicating C−H activation is not the rate-determining step.
Overall summary	In this work, we have demonstrated the successful realization of BHAS-based APEX chemistry. The proposed mechanism was supported by the combined experimental evidences from the EPR studies, radical inhibition, electrochemical reduction, competition experiments, CV measurements, and KIE reaction.

1.20. NMR Spectra

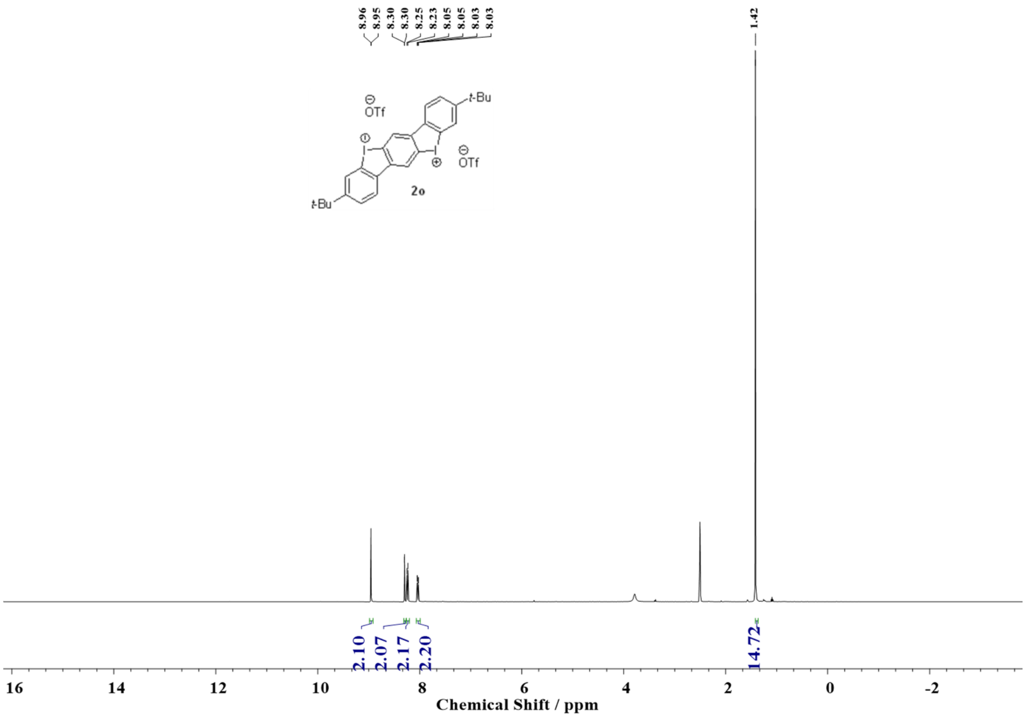

Supplementary Figure 39 ^{1}H NMR (400 MHz, DMSO-d_6) of compound **2o**

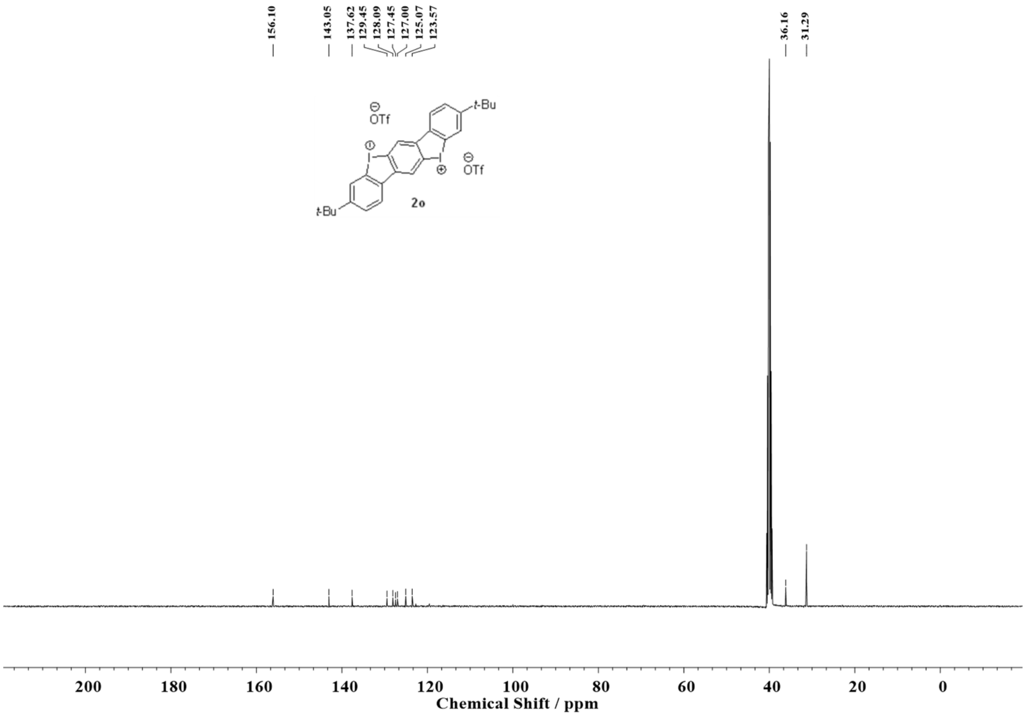

Supplementary Figure 40 ^{13}C NMR (100 MHz, DMSO-d_6) of compound **2o**

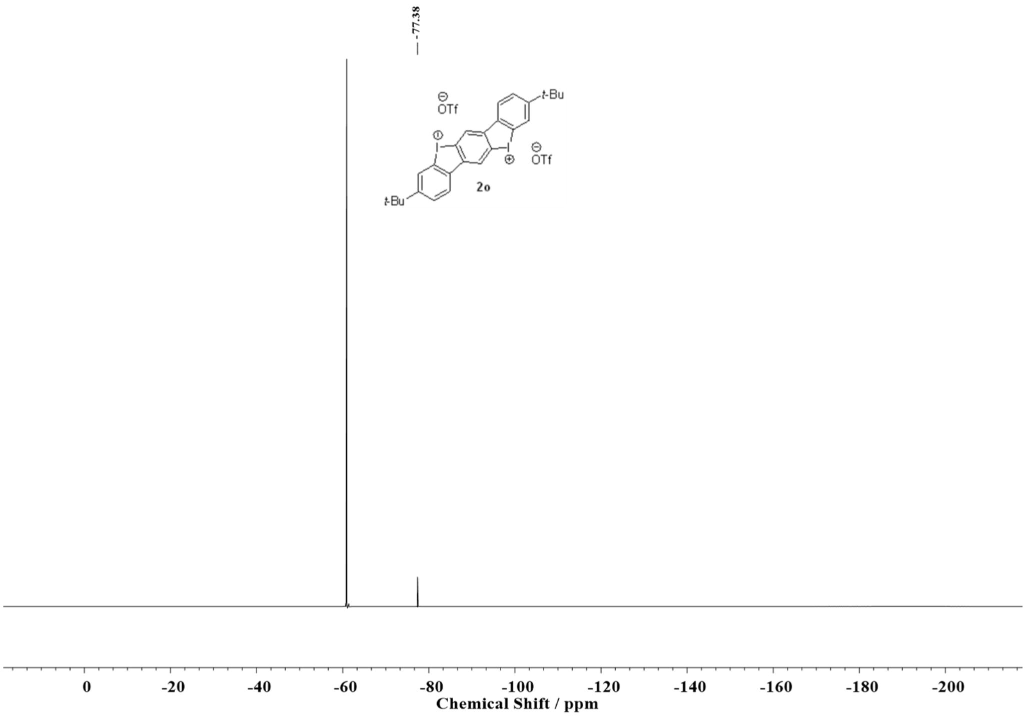

Supplementary Figure 41 ^{19}F NMR (376 MHz, DMSO-d_6) of compound **2o**

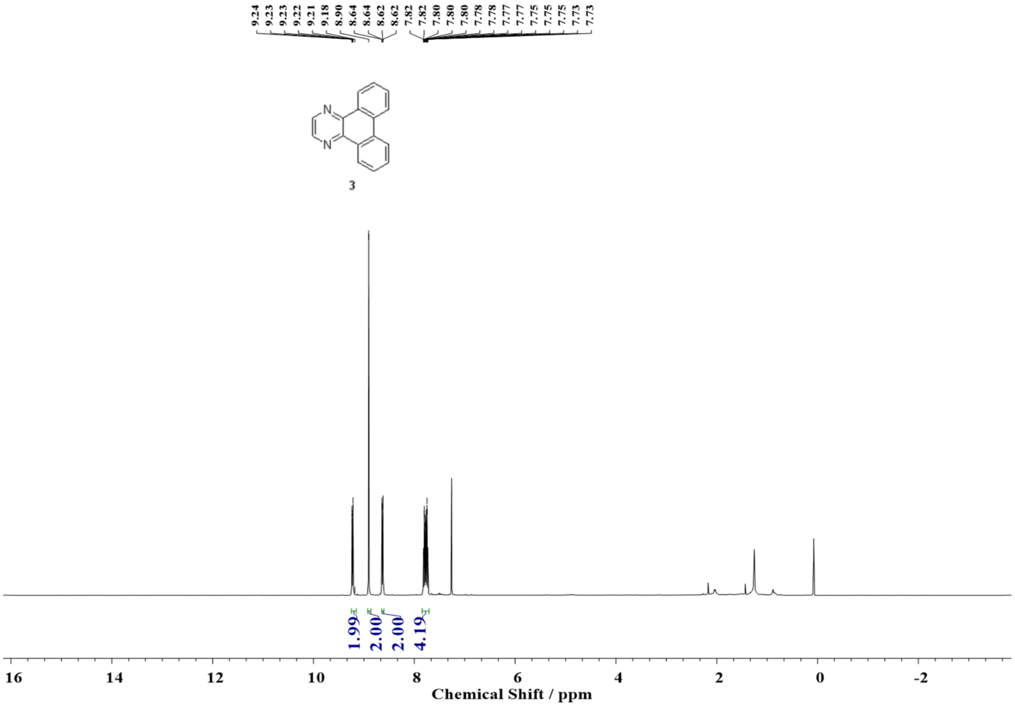

Supplementary Figure 42 ^{1}H NMR (400 MHz, $CDCl_3$) of compound **3**

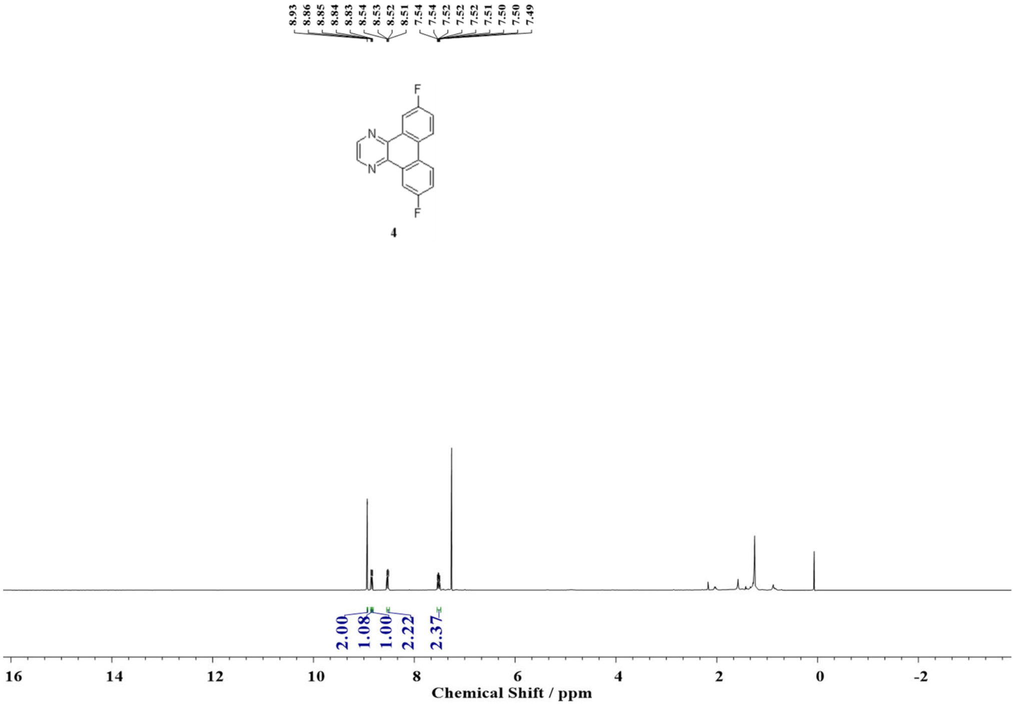

Supplementary Figure 43 ^{1}H NMR (400 MHz, $CDCl_3$) of compound **4**

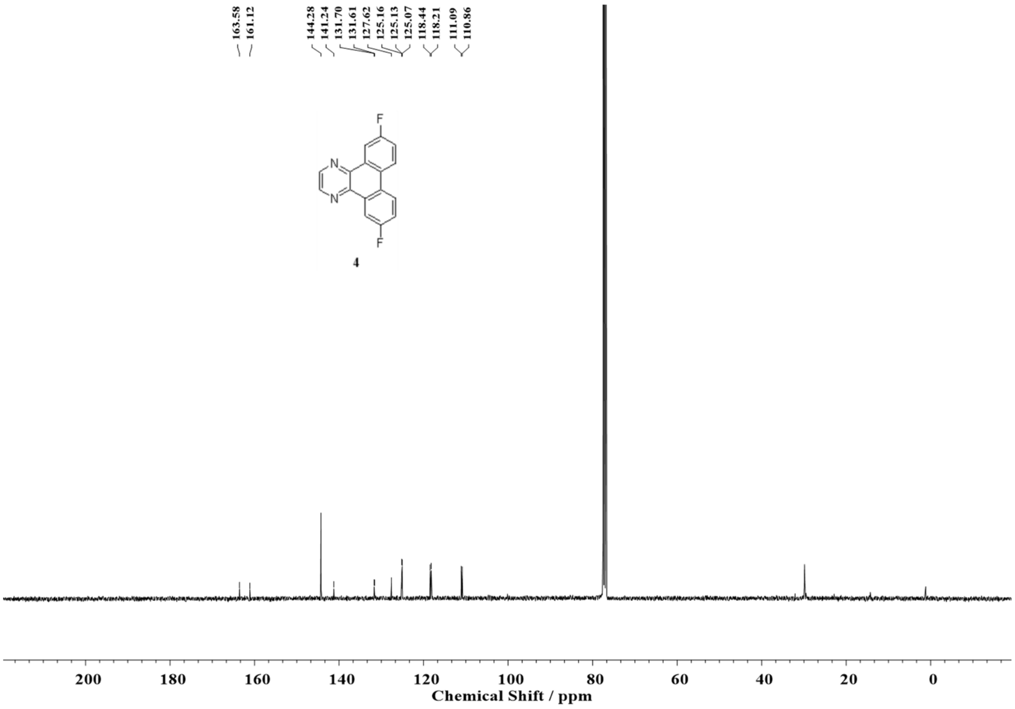

Supplementary Figure 44 ^{13}C NMR (100 MHz, $CDCl_3$) of compound **4**

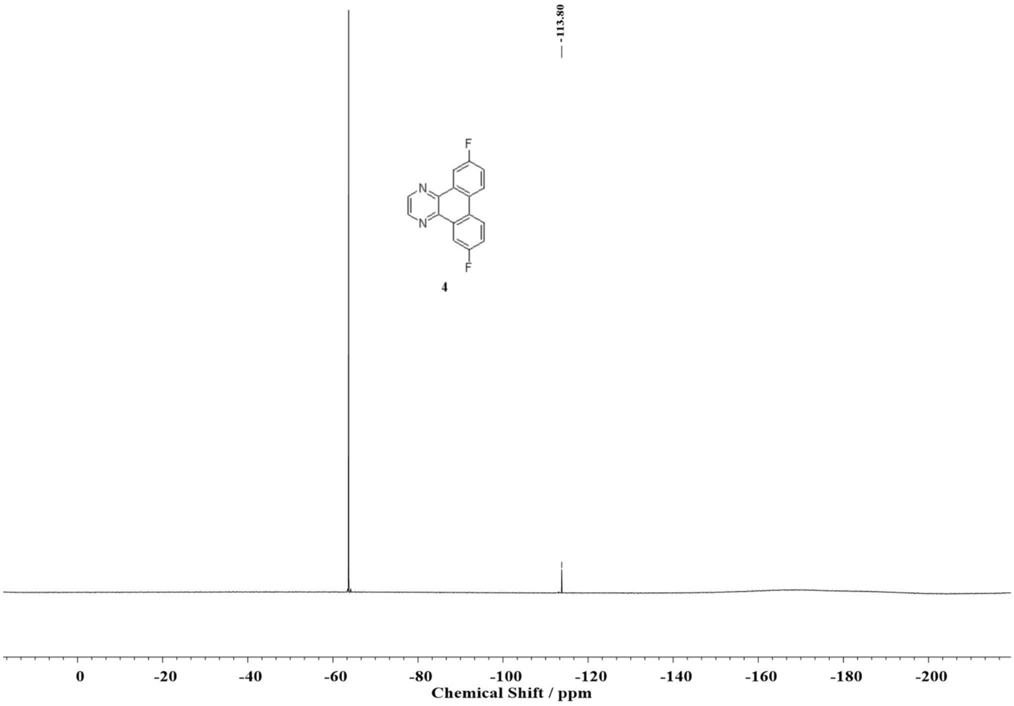

Supplementary Figure 45 ^{19}F NMR (376 MHz, $CDCl_3$) of compound **4**

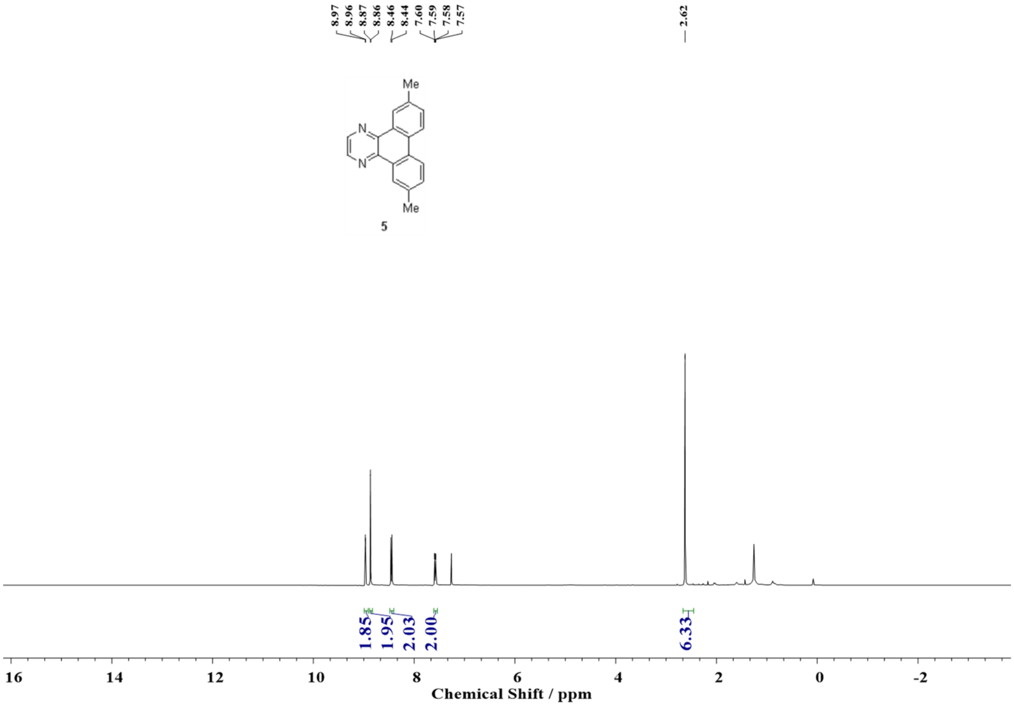

Supplementary Figure 46 ^{1}H NMR (400 MHz, $CDCl_3$) of compound **5**

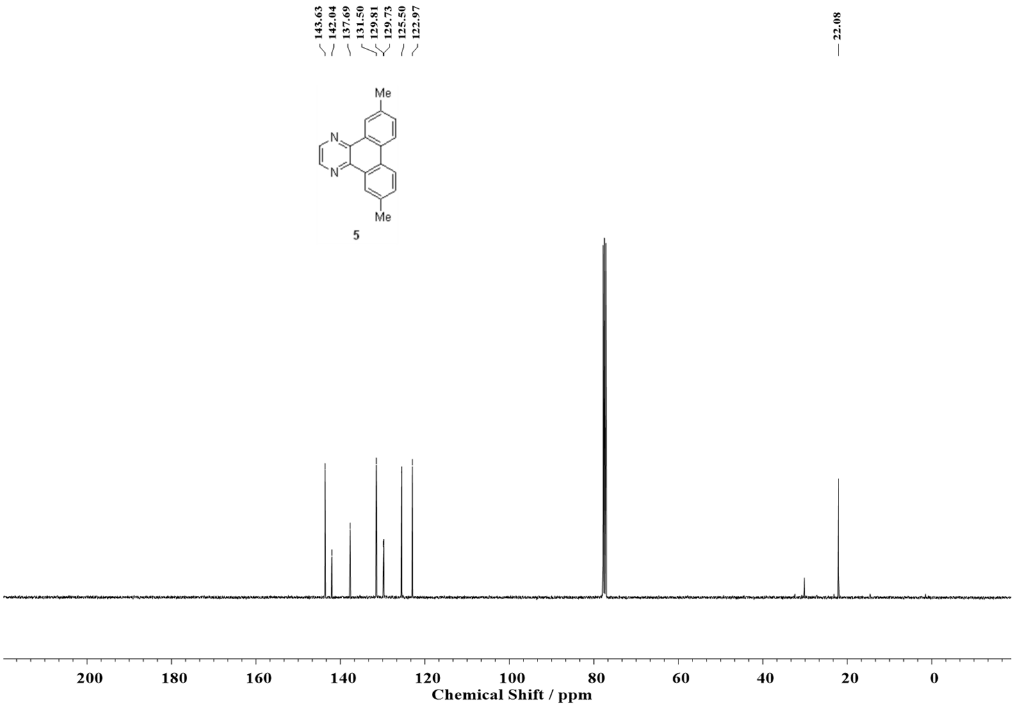

Supplementary Figure 47 ^{13}C NMR (100 MHz, $CDCl_3$) of compound **5**

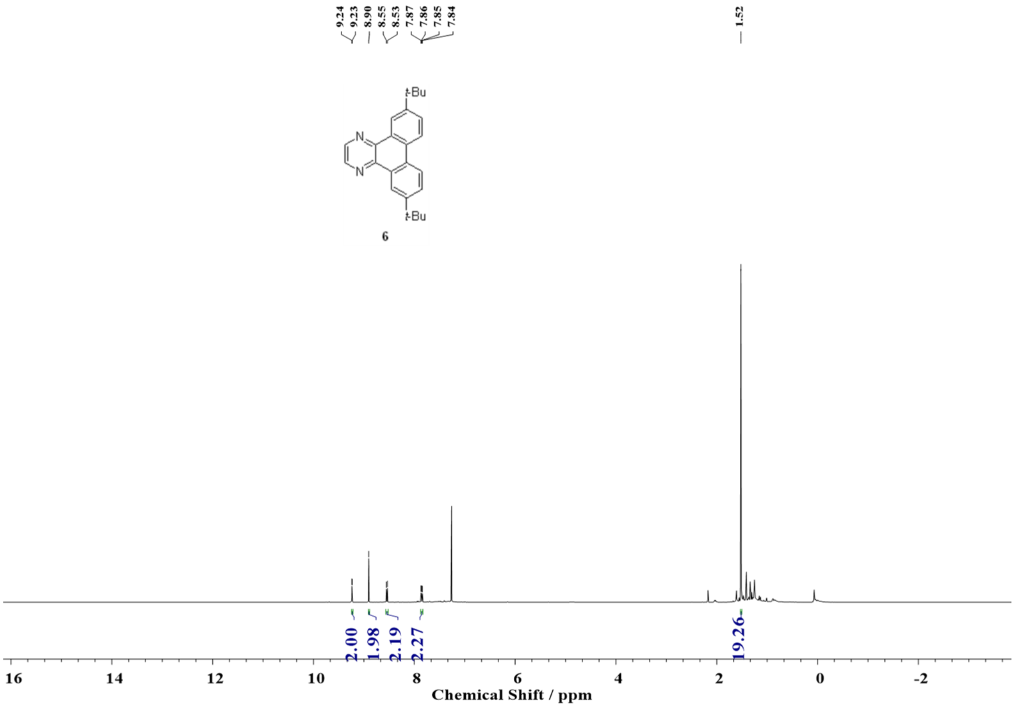

Supplementary Figure 48 ^{1}H NMR (400 MHz, $CDCl_3$) of compound **6**

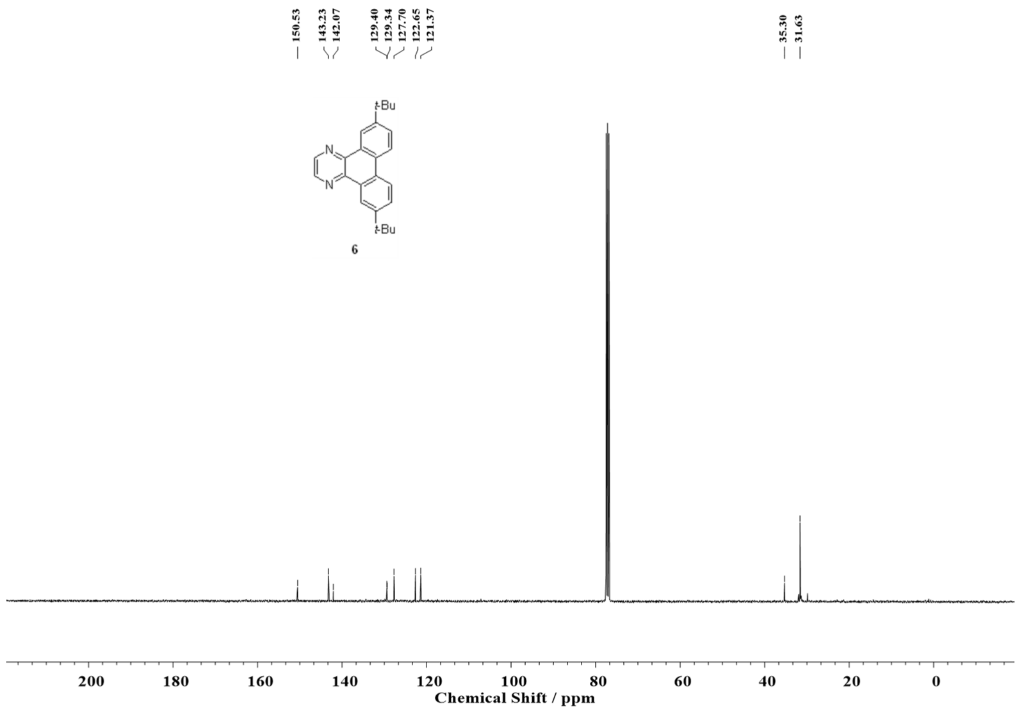

Supplementary Figure 49 ^{13}C NMR (100 MHz, $CDCl_3$) of compound **6**

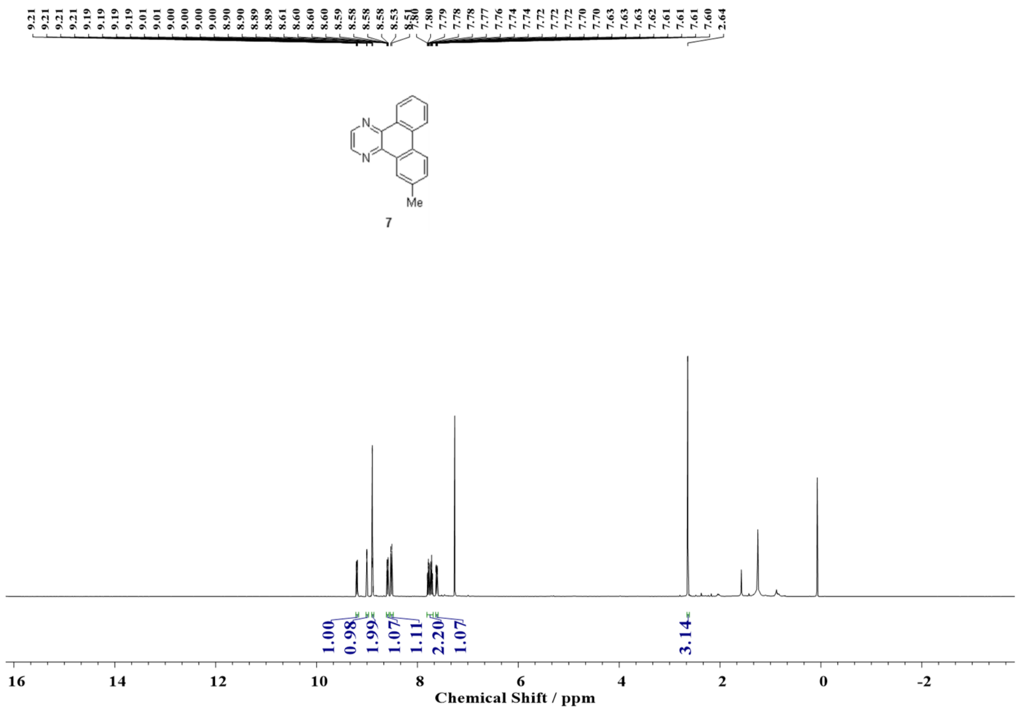

Supplementary Figure 50 ^{1}H NMR (400 MHz, $CDCl_3$) of compound **7**

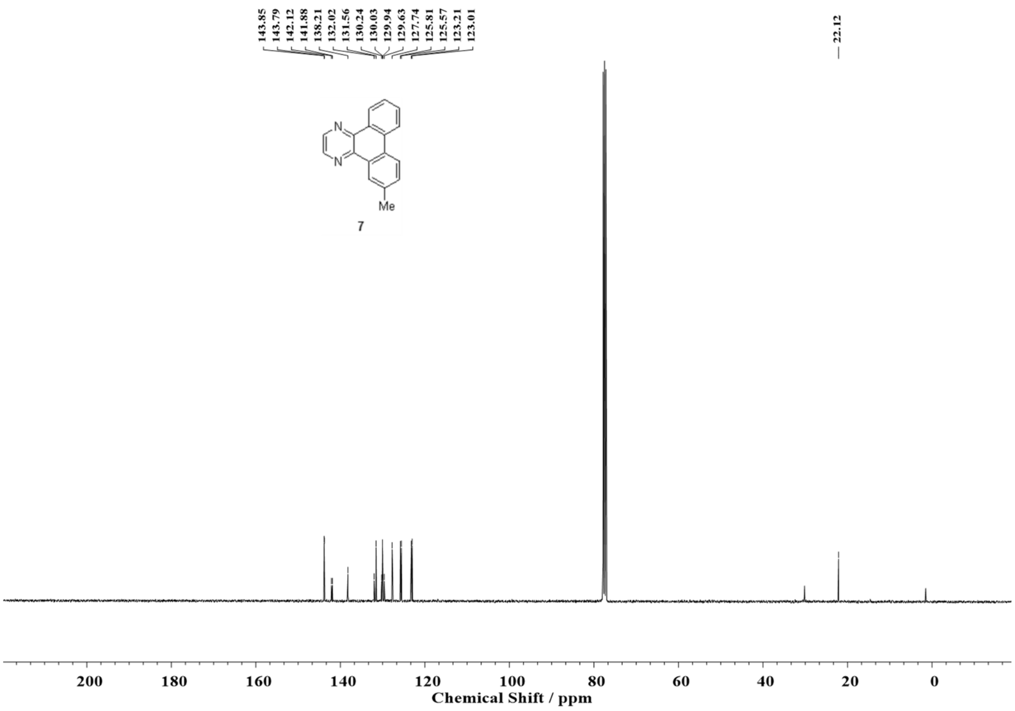

Supplementary Figure 51 ^{13}C NMR (100 MHz, $CDCl_3$) of compound **7**

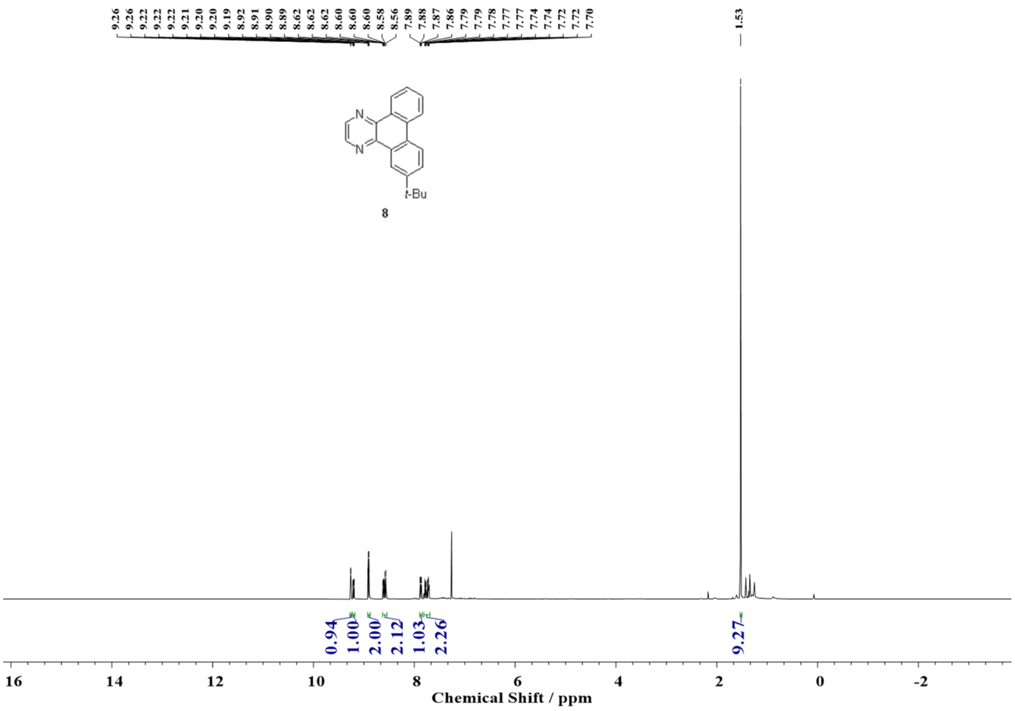

Supplementary Figure 52 ^{1}H NMR (400 MHz, $CDCl_3$) of compound **8**

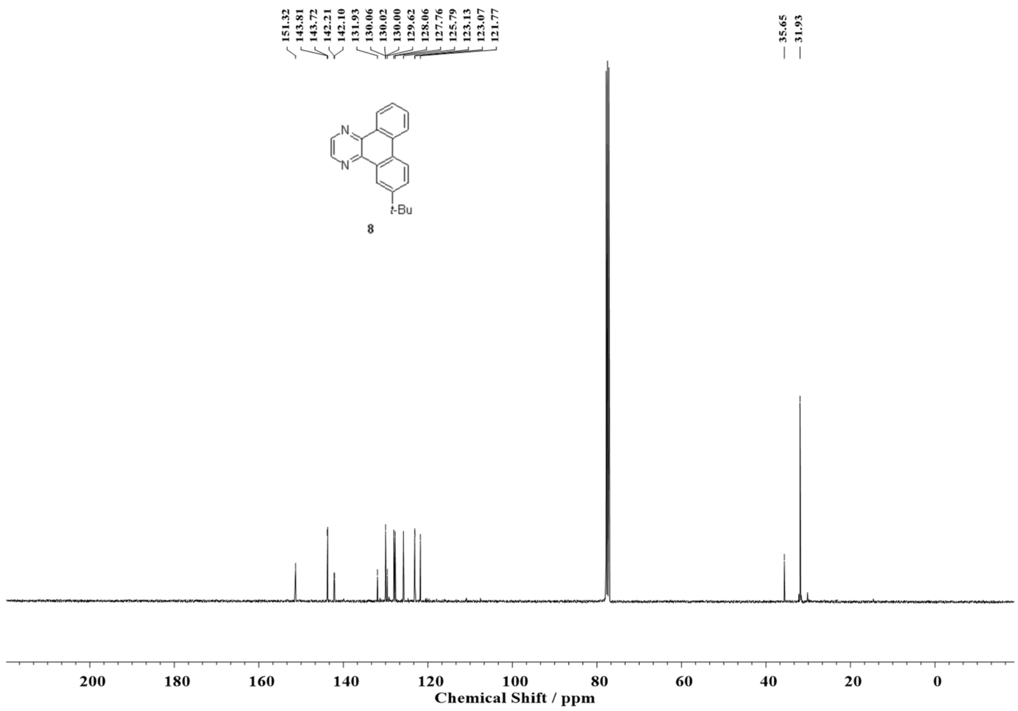

Supplementary Figure 53 ^{13}C NMR (100 MHz, $CDCl_3$) of compound **8**

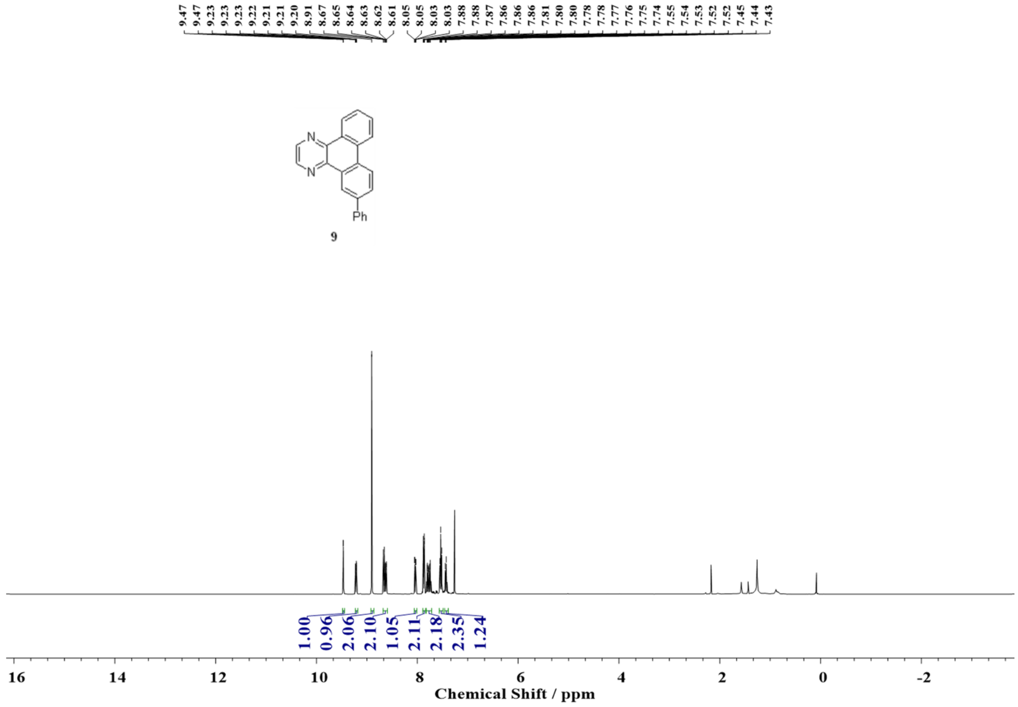

Supplementary Figure 54 ^{1}H NMR (400 MHz, $CDCl_3$) of compound **9**

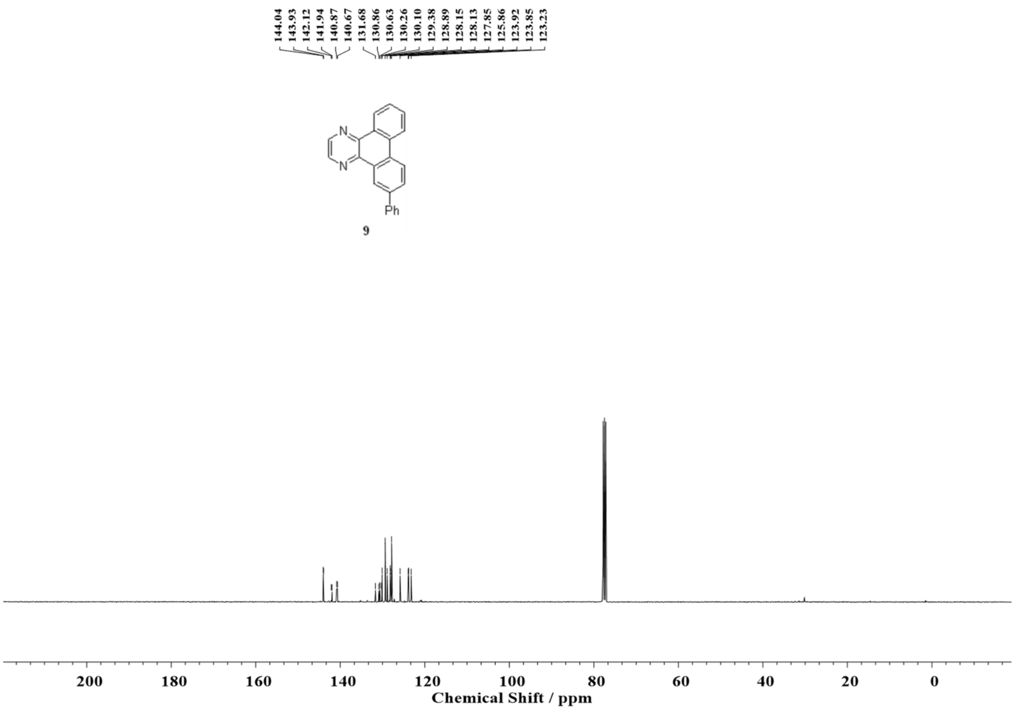

Supplementary Figure 55 ^{13}C NMR (100 MHz, $CDCl_3$) of compound **9**

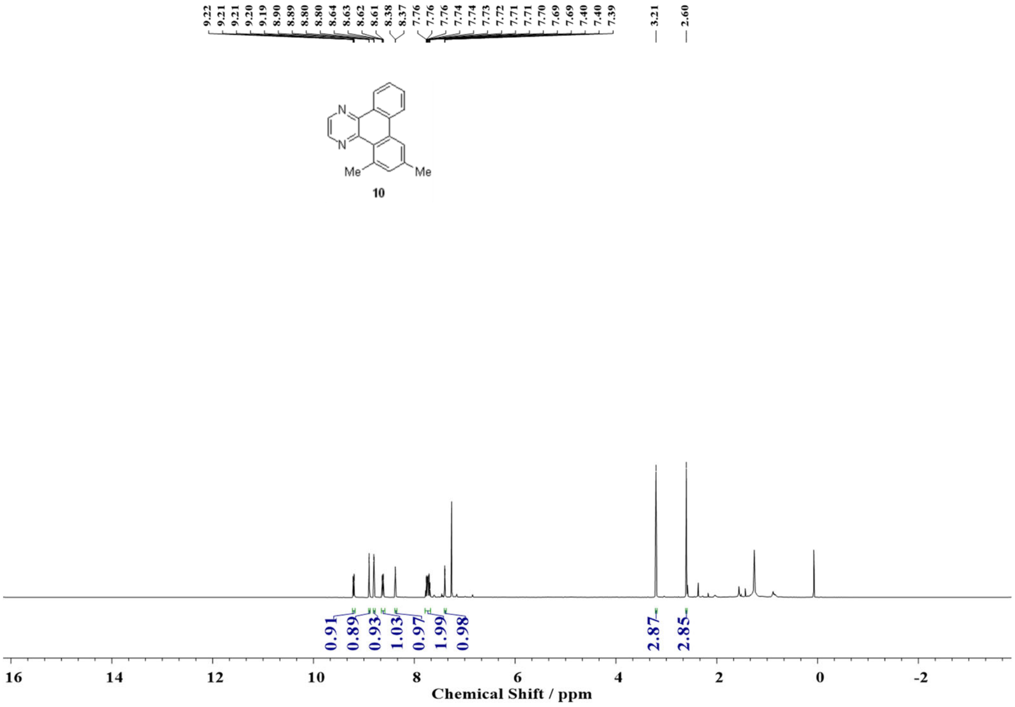

Supplementary Figure 56 ^{1}H NMR (400 MHz, $CDCl_3$) of compound **10**

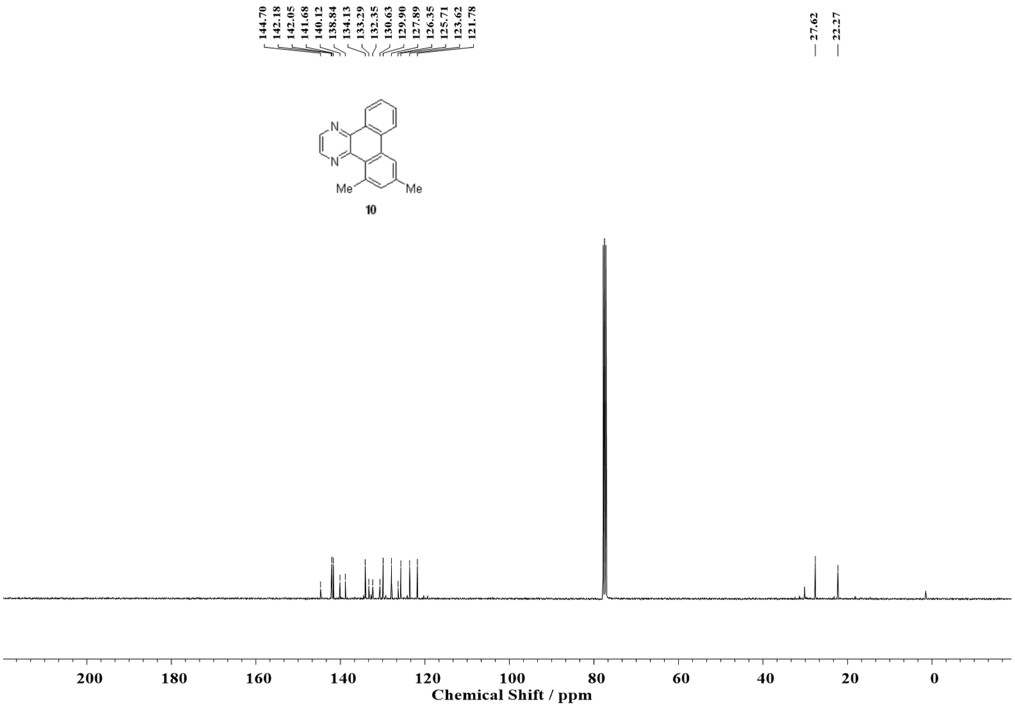

Supplementary Figure 57 ^{13}C NMR (100 MHz, $CDCl_3$) of compound **10**

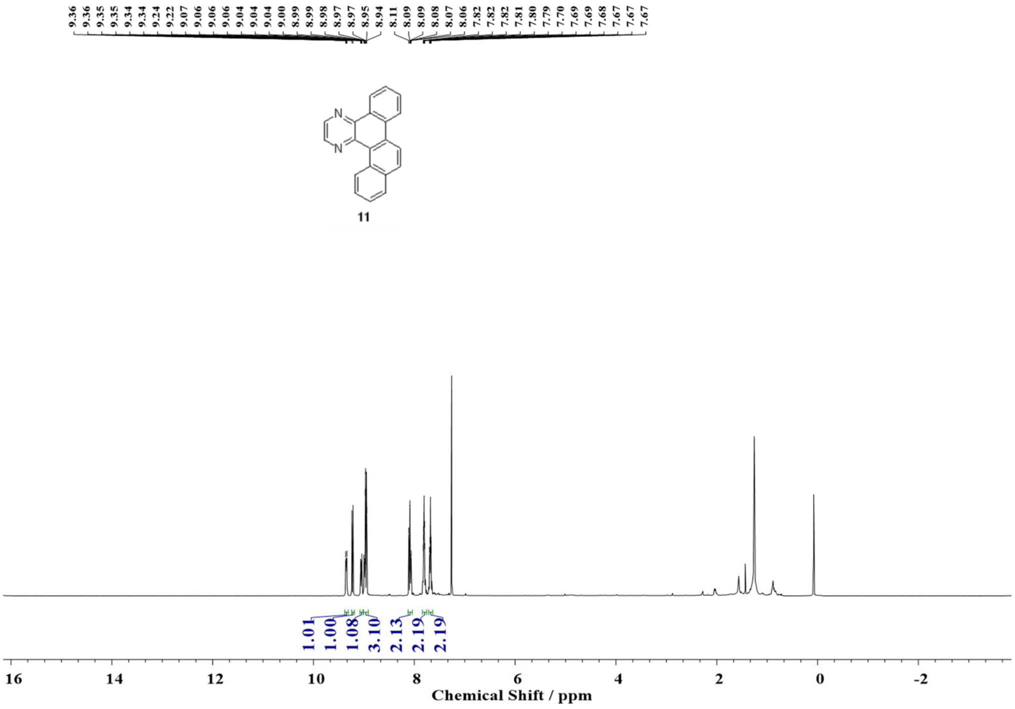

Supplementary Figure 58 ^{1}H NMR (400 MHz, $CDCl_3$) of compound **11**

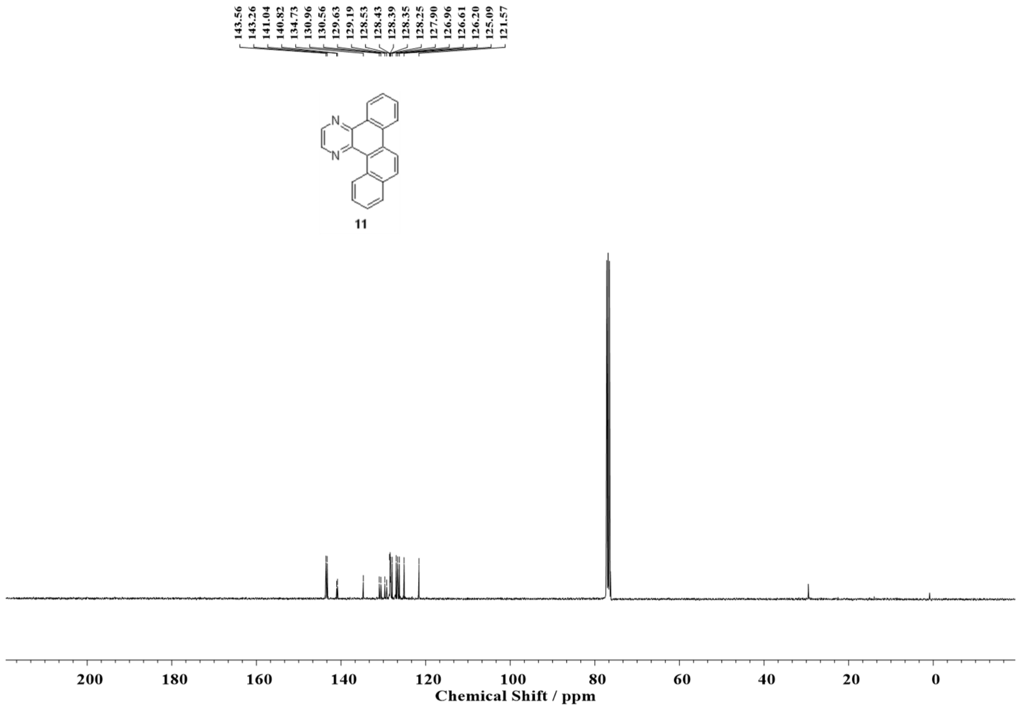

Supplementary Figure 59 ^{13}C NMR (100 MHz, $CDCl_3$) of compound **11**

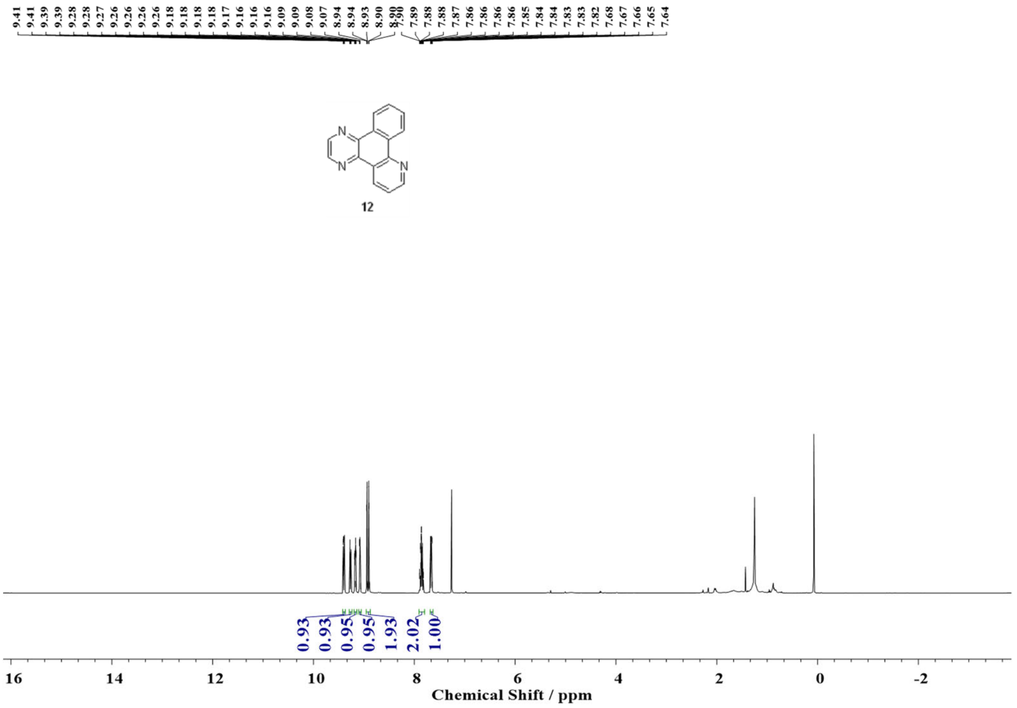

Supplementary Figure 60 ^{1}H NMR (400 MHz, $CDCl_3$) of compound **12**

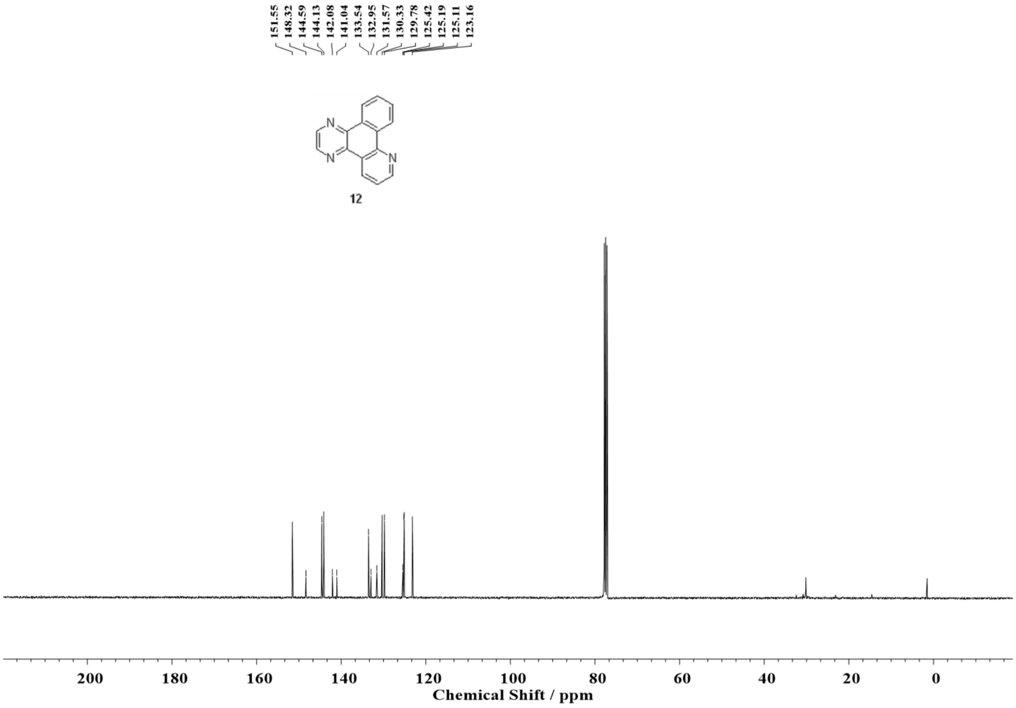

Supplementary Figure 61 ^{13}C NMR (100 MHz, $CDCl_3$) of compound **12**

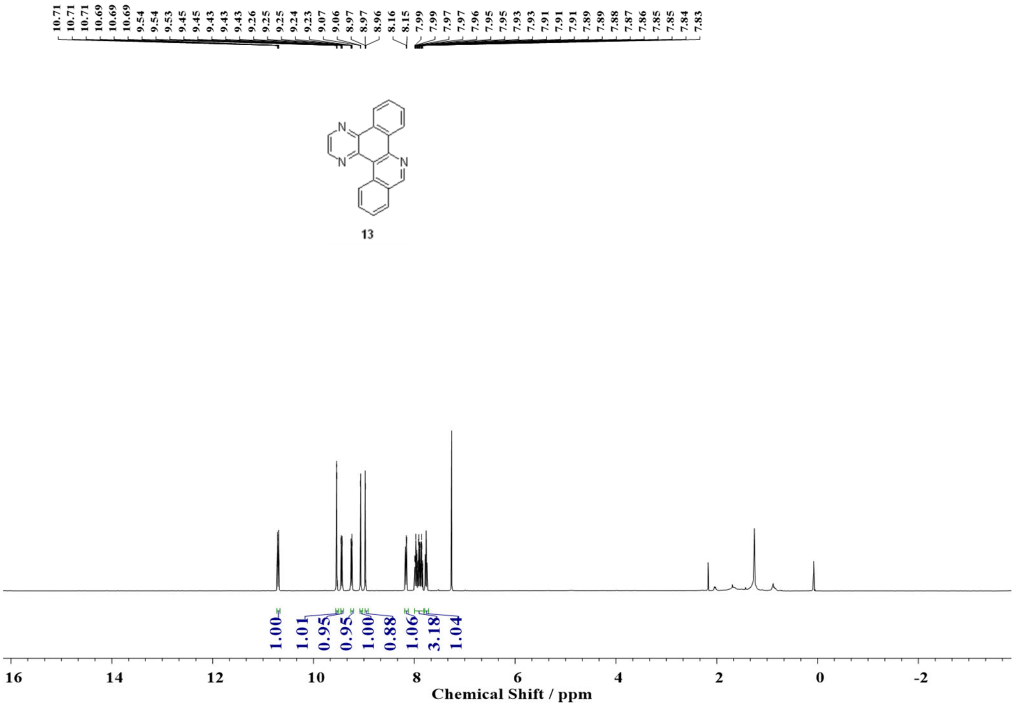

Supplementary Figure 62 ^{1}H NMR (400 MHz, $CDCl_3$) of compound **13**

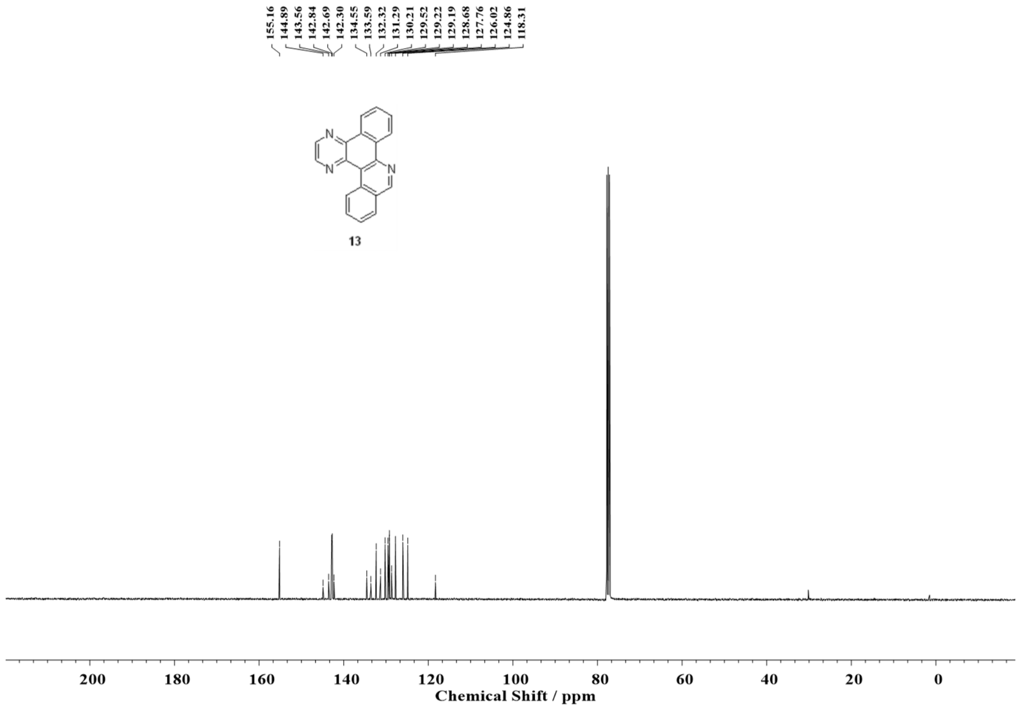

Supplementary Figure 63 ^{13}C NMR (100 MHz, $CDCl_3$) of compound **13**

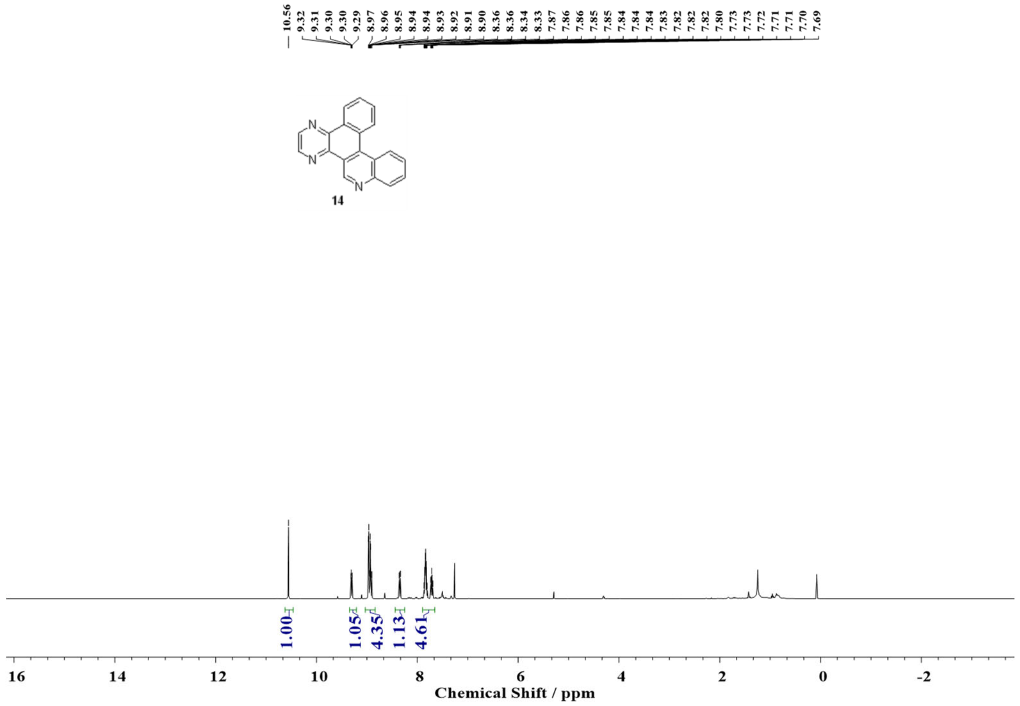

Supplementary Figure 64 ^{1}H NMR (400 MHz, $CDCl_3$) of compound **14**

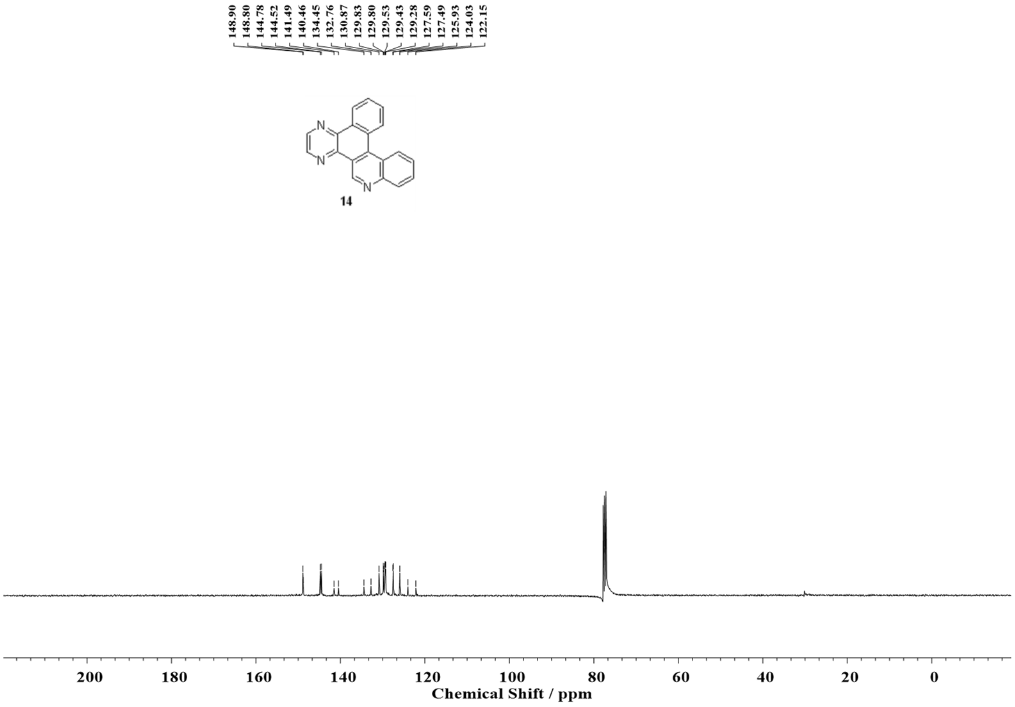

Supplementary Figure 65 ^{13}C NMR (100 MHz, $CDCl_3$) of compound **14**

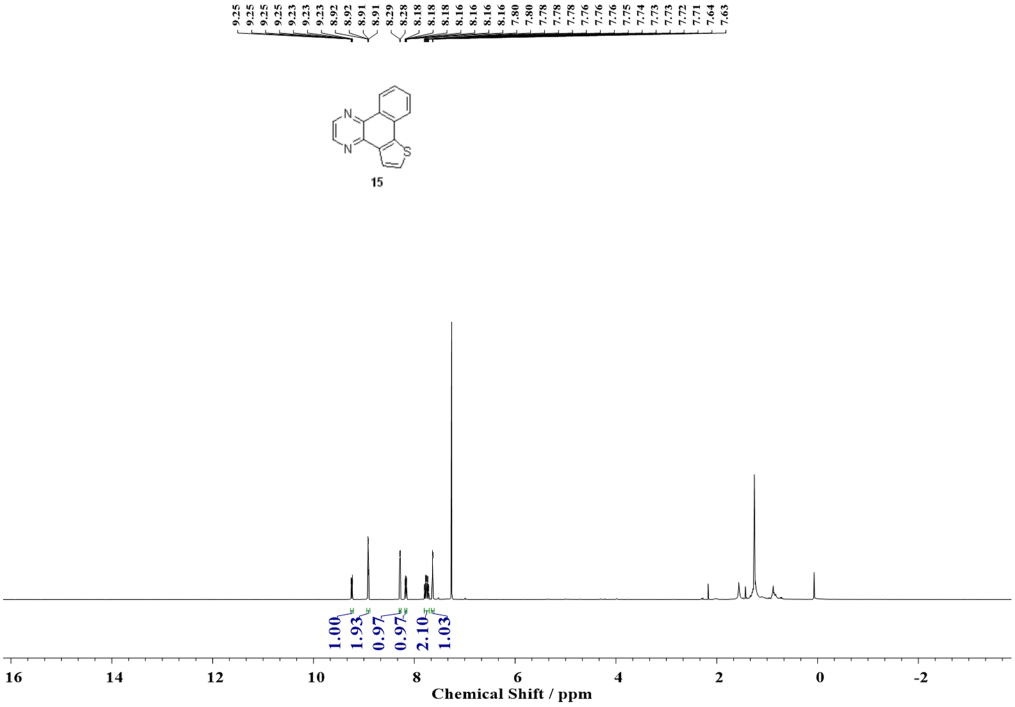

Supplementary Figure 66 ^{1}H NMR (400 MHz, $CDCl_3$) of compound **15**

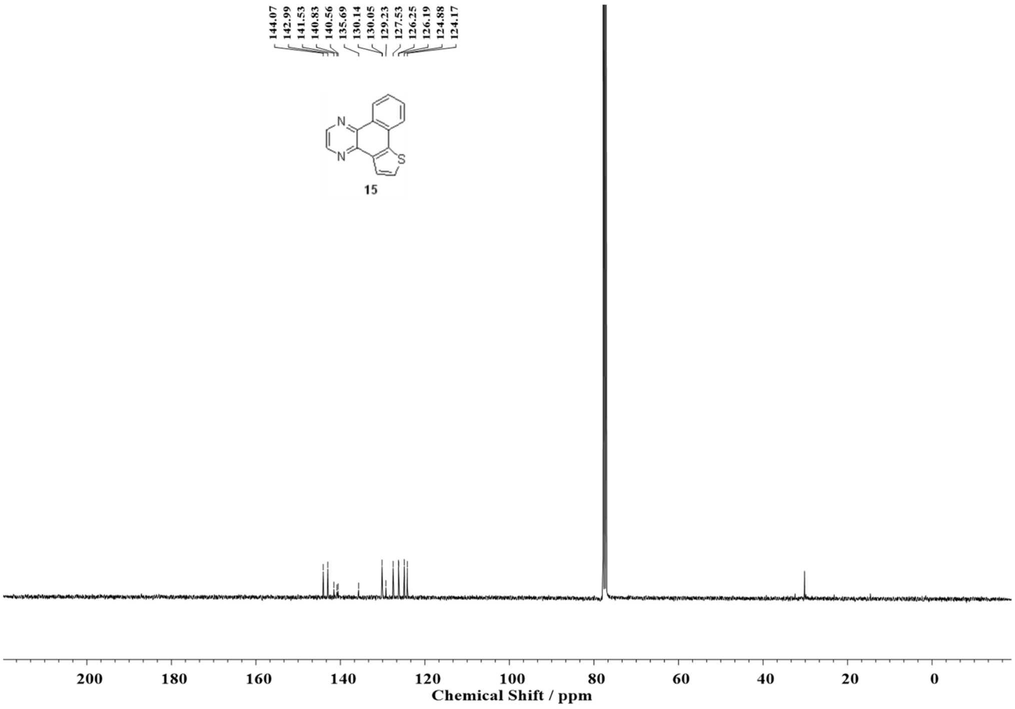

Supplementary Figure 67 ^{13}C NMR (100 MHz, $CDCl_3$) of compound **15**

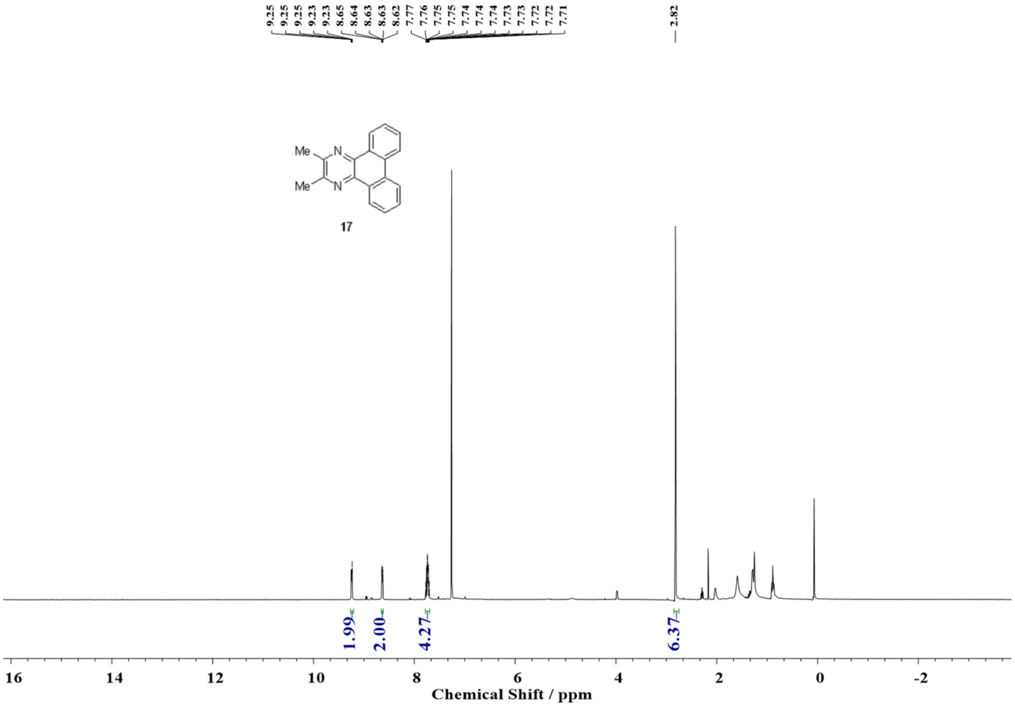

Supplementary Figure 68 ^{1}H NMR (400 MHz, $CDCl_3$) of compound **17**

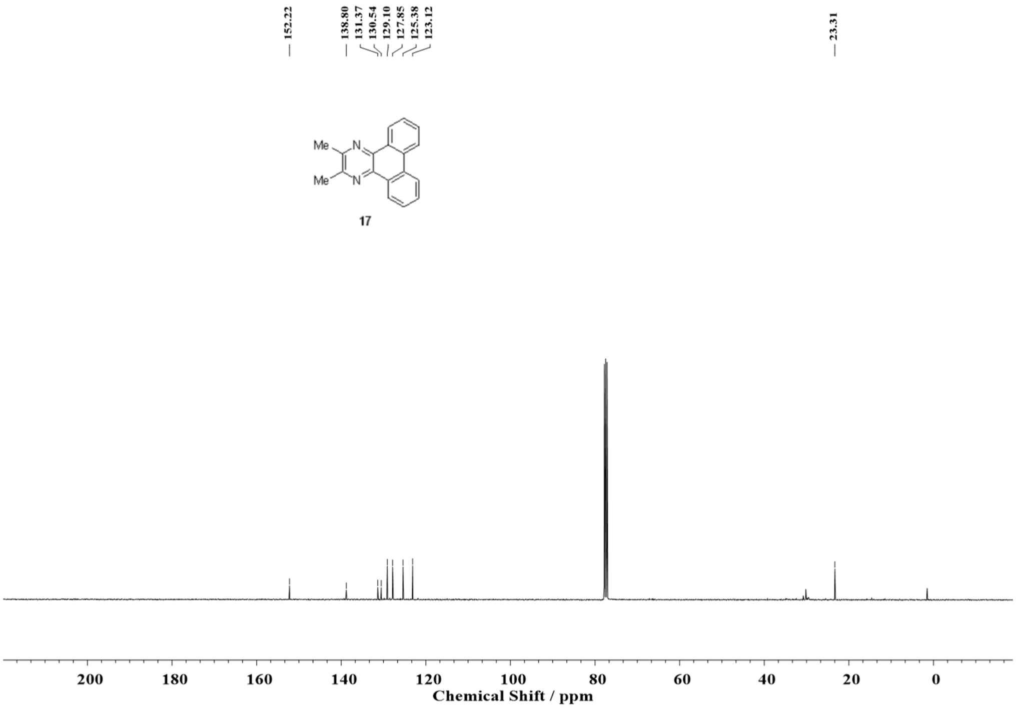

Supplementary Figure 69 ^{13}C NMR (100 MHz, $CDCl_3$) of compound **17**

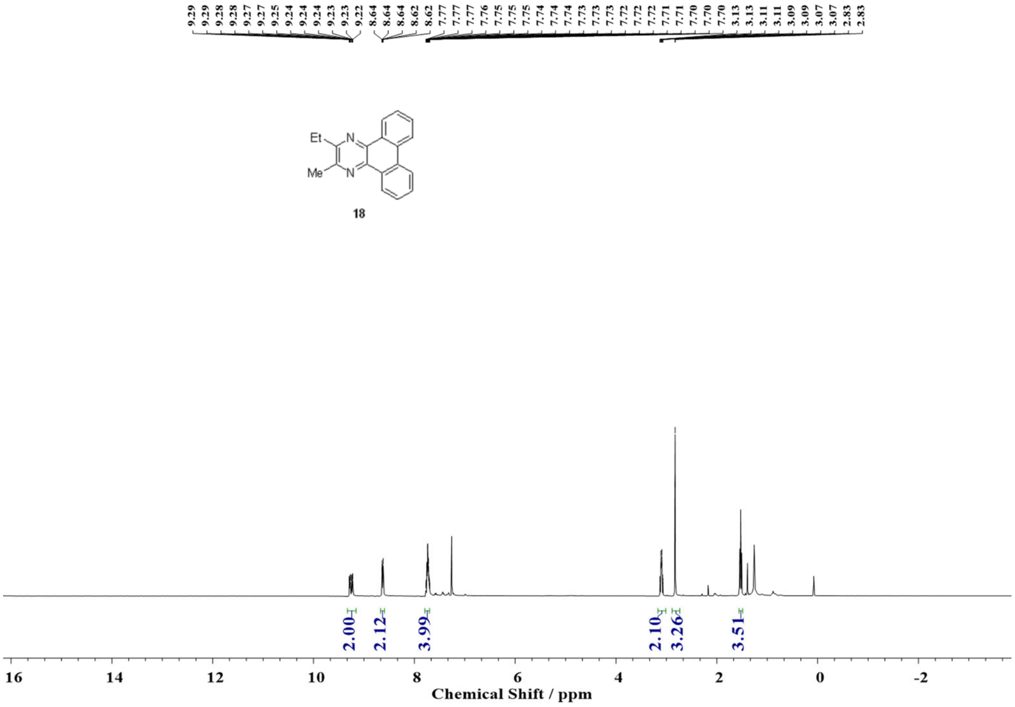

Supplementary Figure 70 ^{1}H NMR (400 MHz, $CDCl_3$) of compound **18**

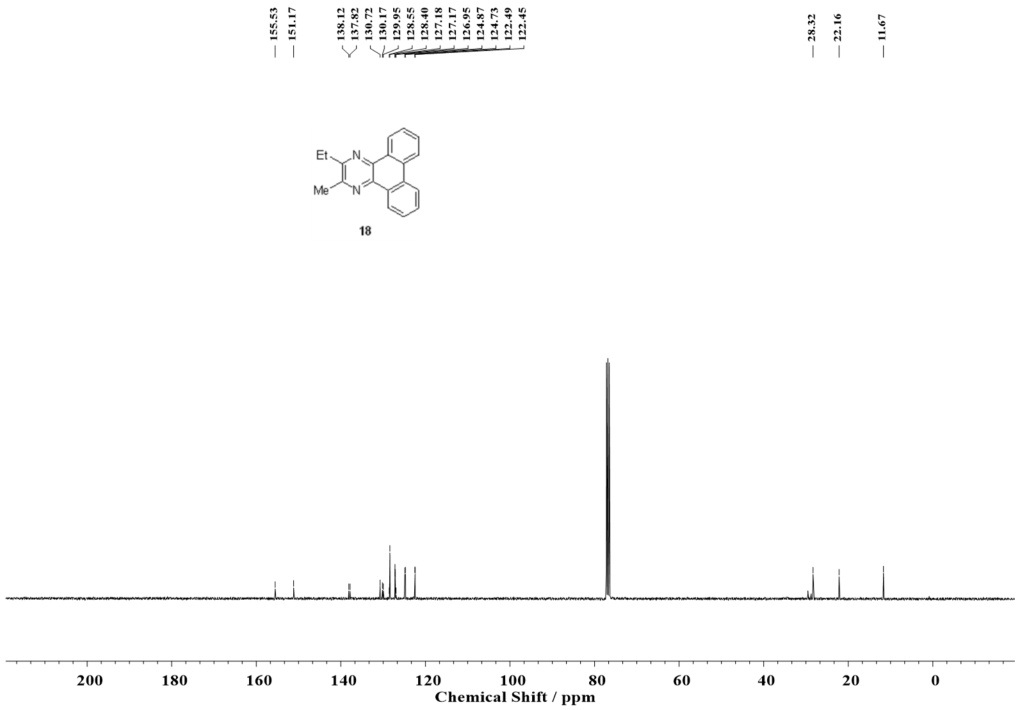

Supplementary Figure 71 ^{13}C NMR (100 MHz, $CDCl_3$) of compound **18**

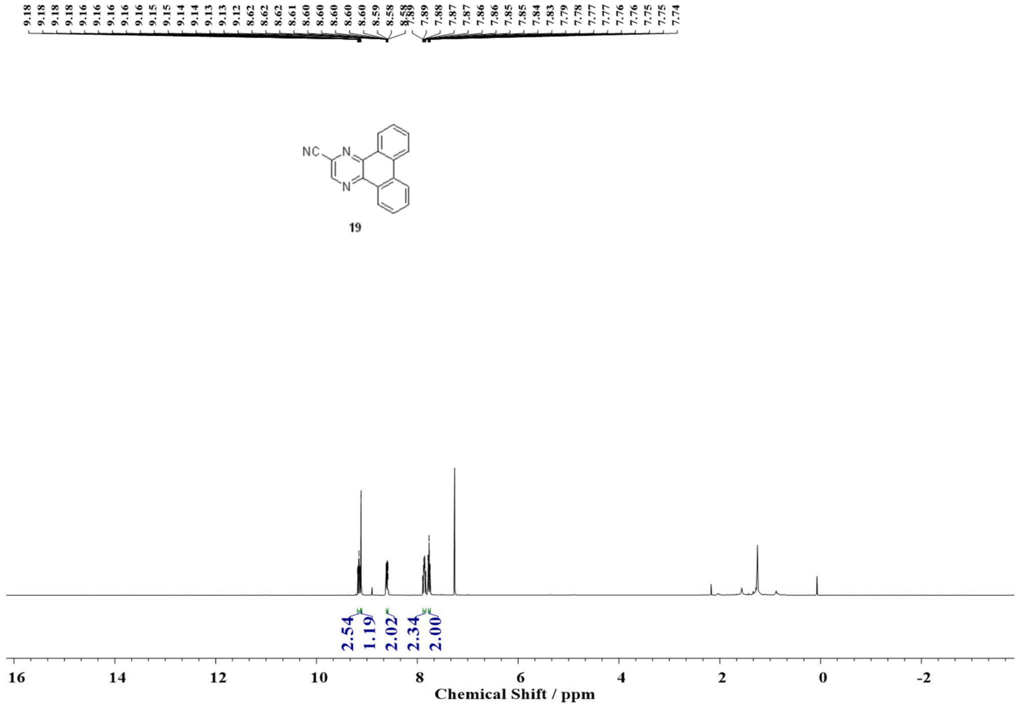

Supplementary Figure 72 ^{1}H NMR (400 MHz, $CDCl_3$) of compound **19**

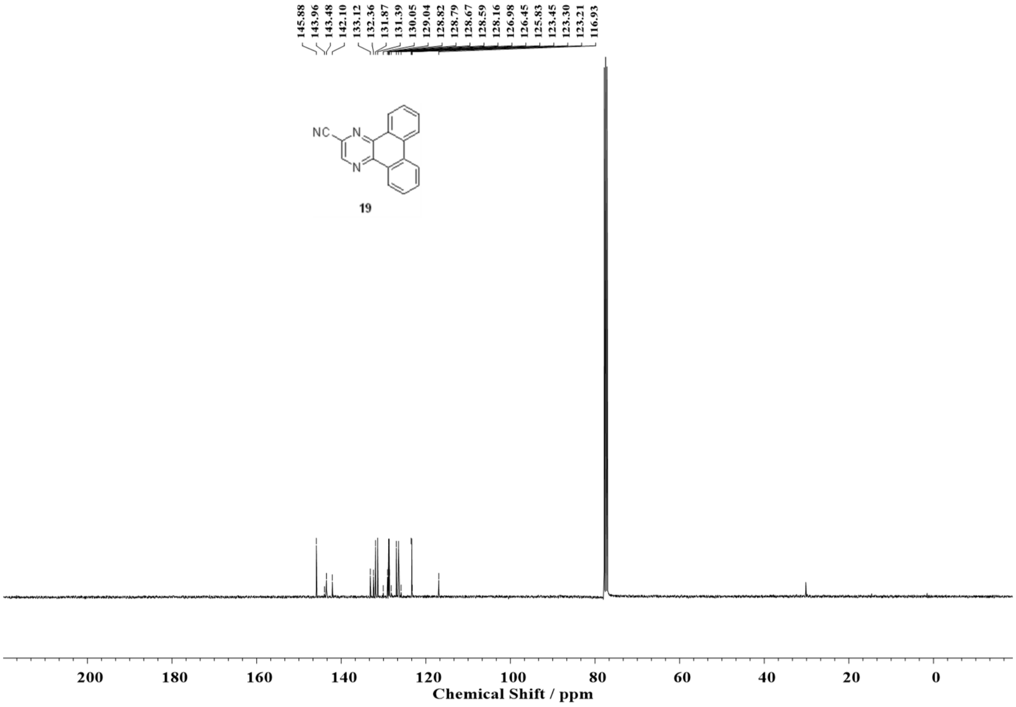

Supplementary Figure 73 ^{13}C NMR (100 MHz, $CDCl_3$) of compound **19**

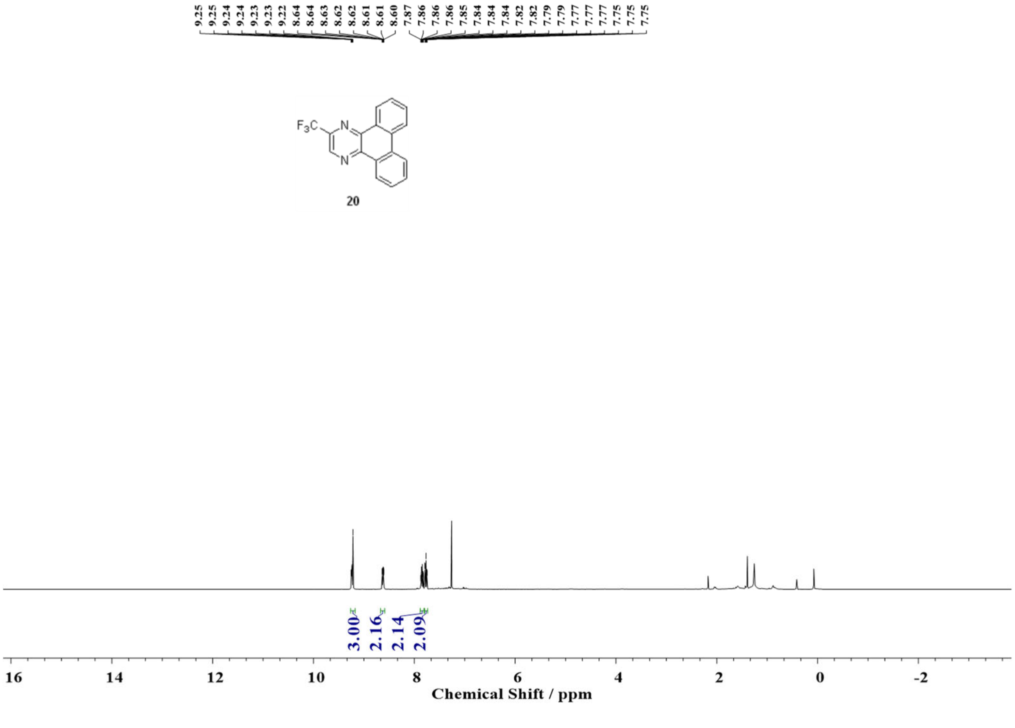

Supplementary Figure 74 ^{1}H NMR (400 MHz, $CDCl_3$) of compound **20**

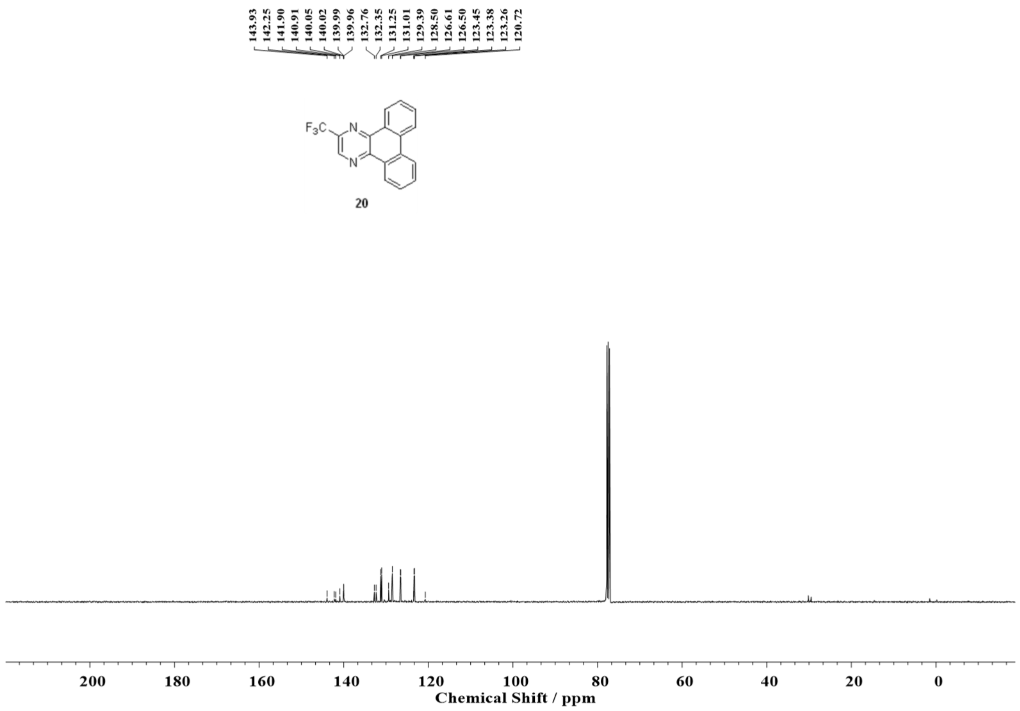

Supplementary Figure 75 ^{13}C NMR (100 MHz, $CDCl_3$) of compound **20**

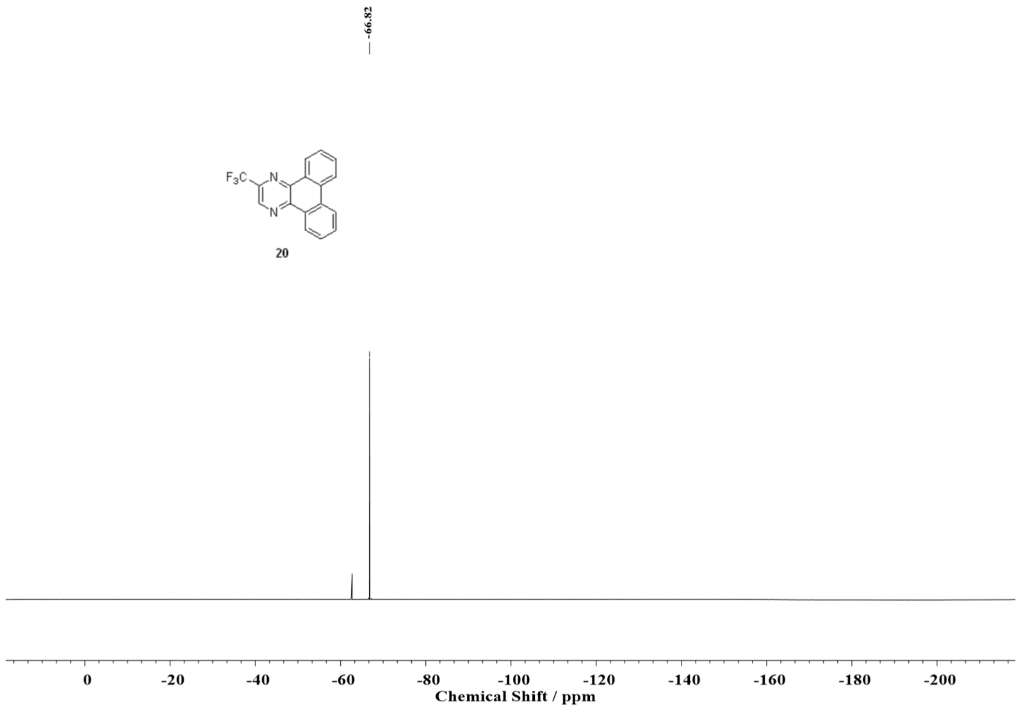

Supplementary Figure 76 ^{19}F NMR (376 MHz, $CDCl_3$) of compound **20**

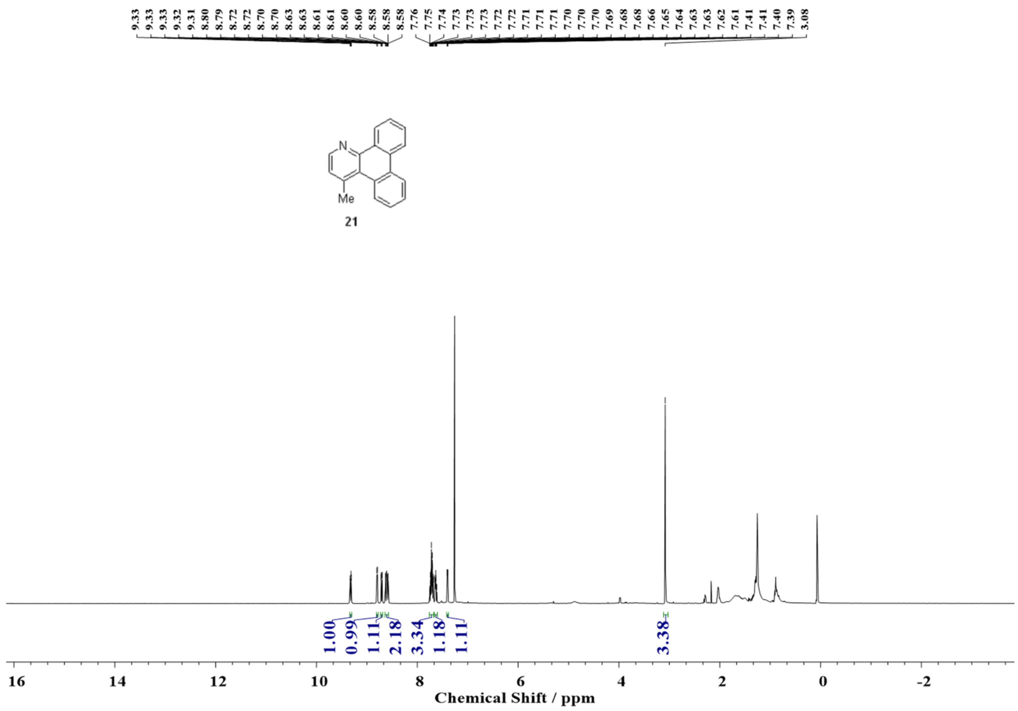

Supplementary Figure 77 ^{1}H NMR (400 MHz, $CDCl_3$) of compound **21**

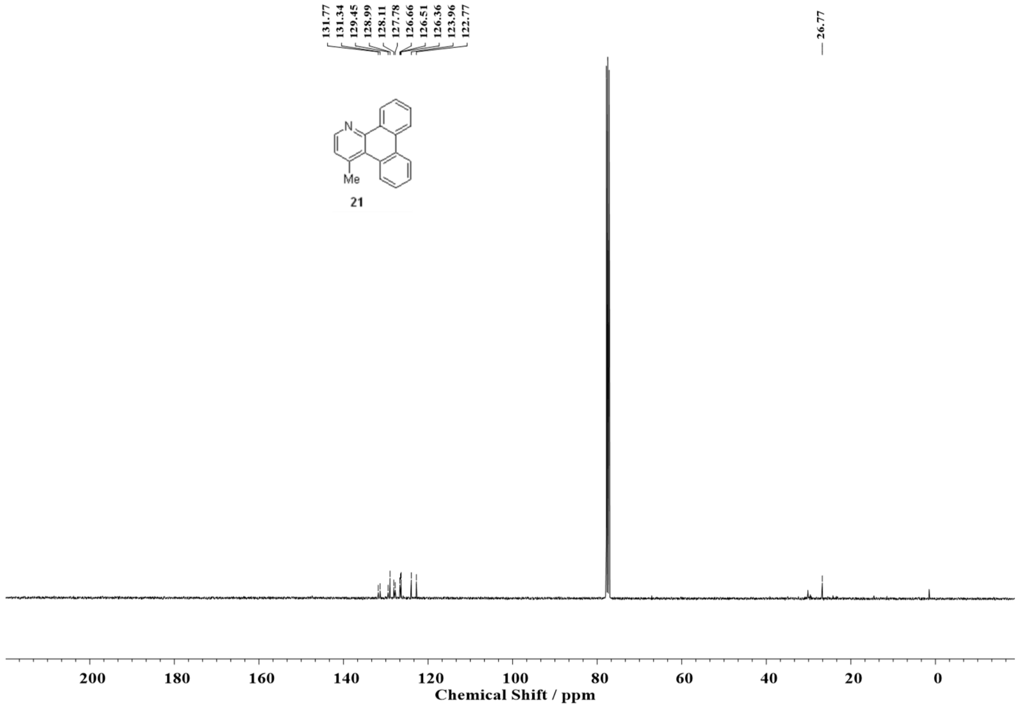

Supplementary Figure 78 ^{13}C NMR (100 MHz, $CDCl_3$) of compound **21**

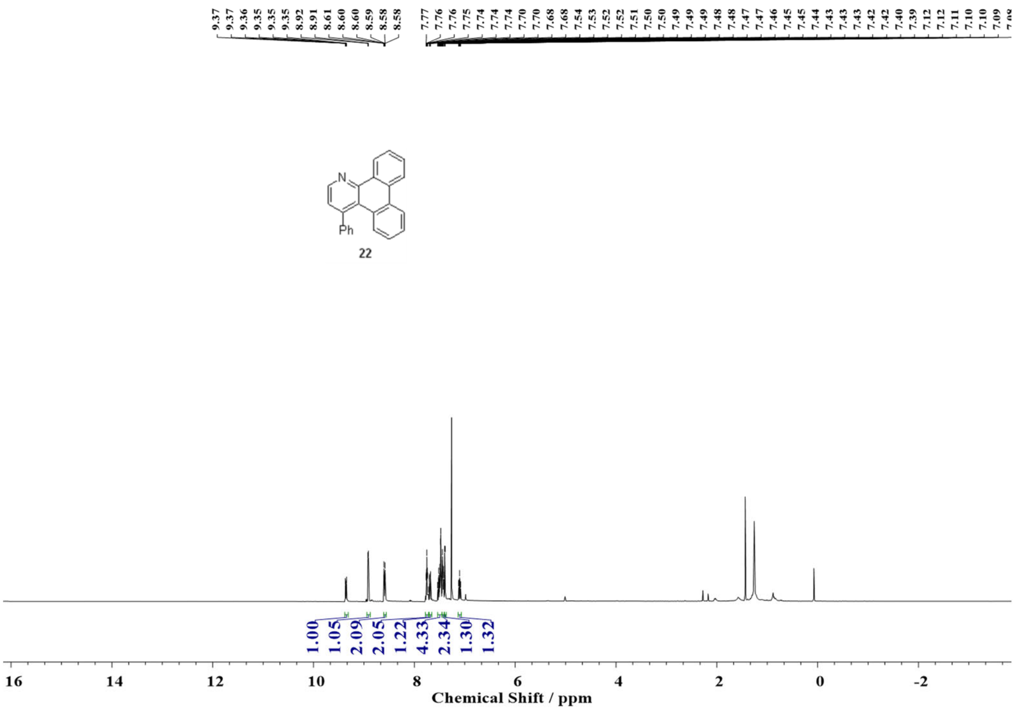

Supplementary Figure 79 ^{1}H NMR (400 MHz, $CDCl_3$) of compound **22**

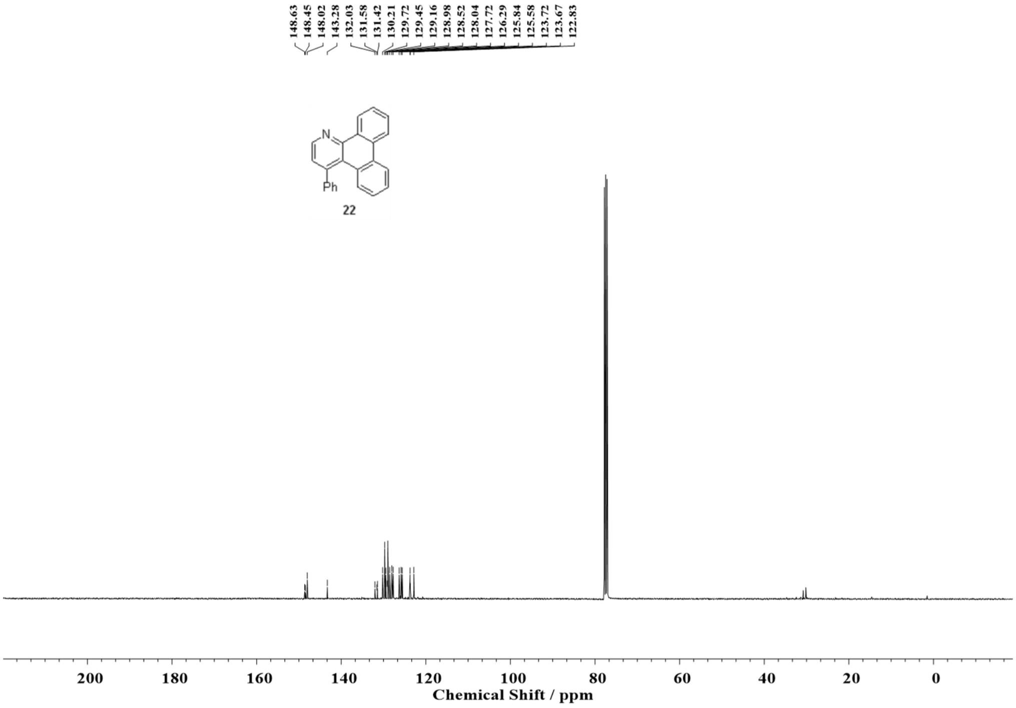

Supplementary Figure 80 ^{13}C NMR (100 MHz, $CDCl_3$) of compound **22**

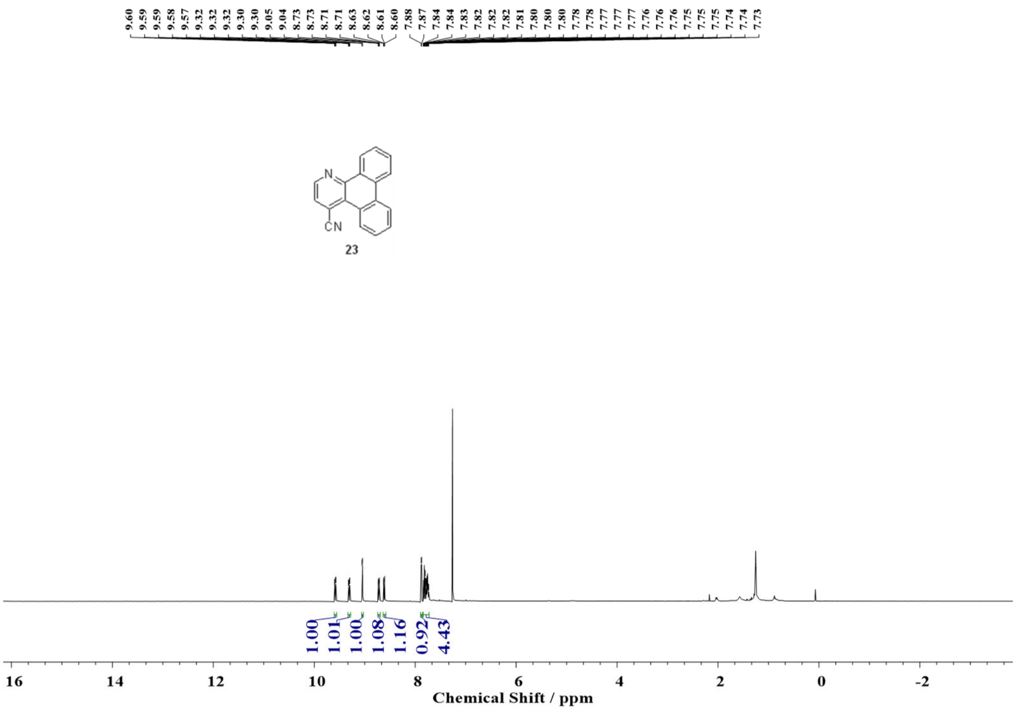

Supplementary Figure 81 ^{1}H NMR (400 MHz, $CDCl_3$) of compound **23**

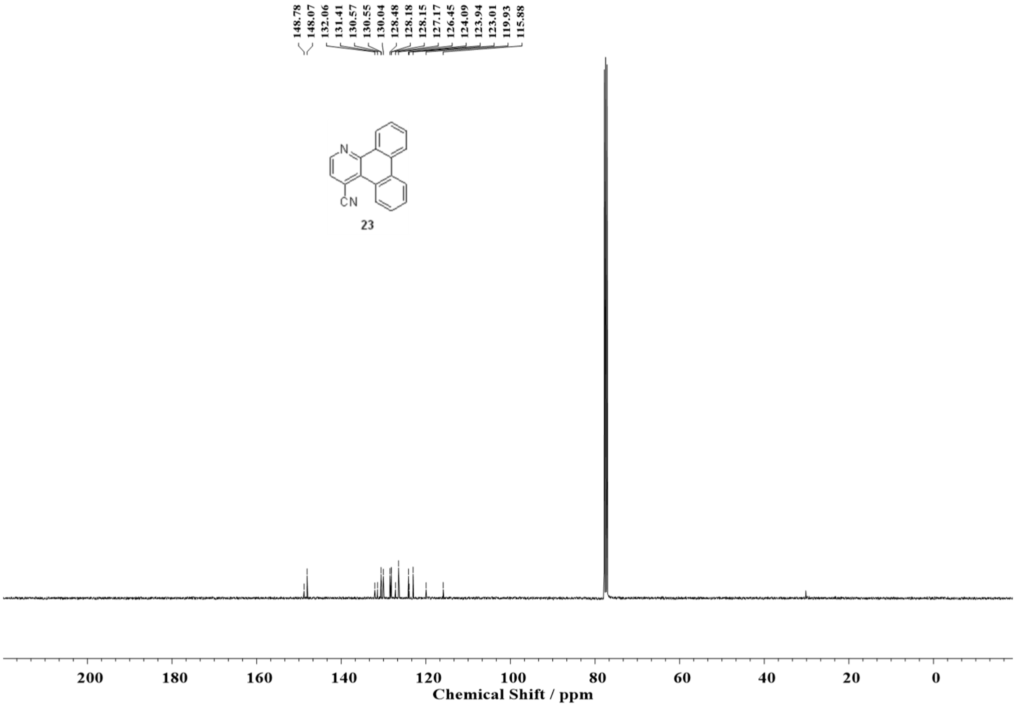

Supplementary Figure 82 ^{13}C NMR (100 MHz, $CDCl_3$) of compound **23**

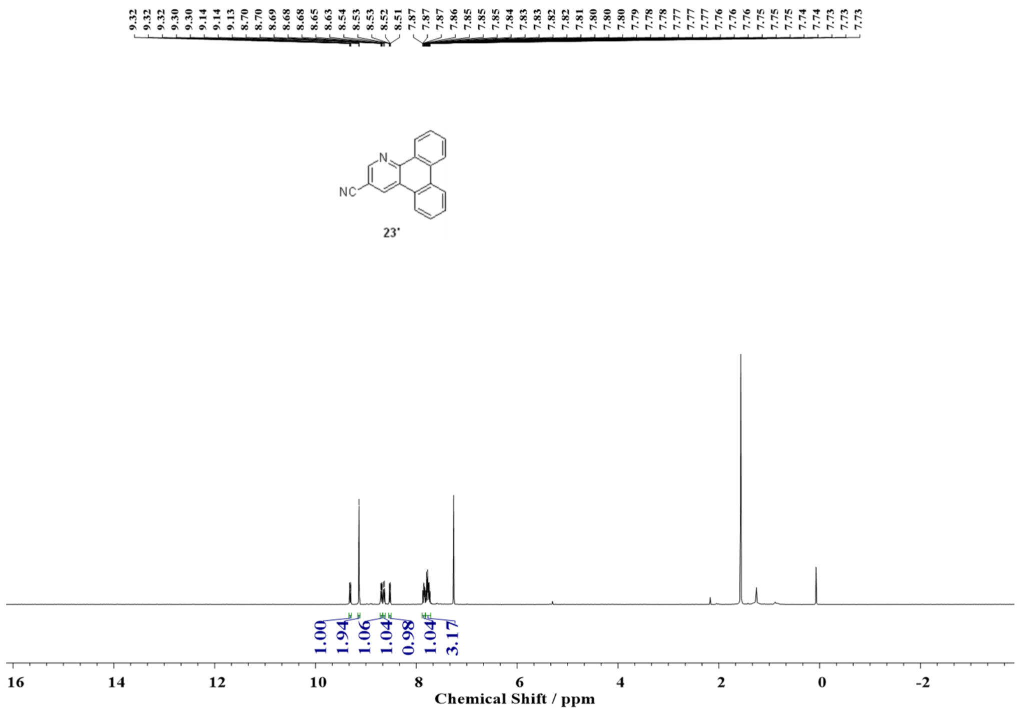

Supplementary Figure 83 ^{1}H NMR (400 MHz, $CDCl_3$) of compound **23'**

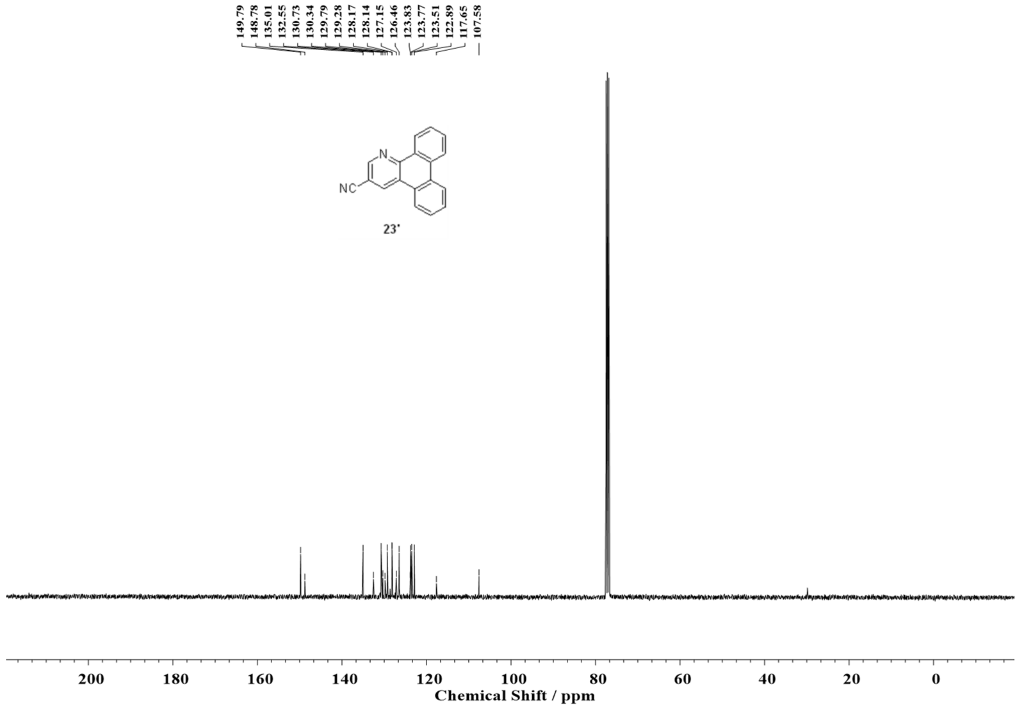

Supplementary Figure 84 ^{13}C NMR (100 MHz, $CDCl_3$) of compound **23'**

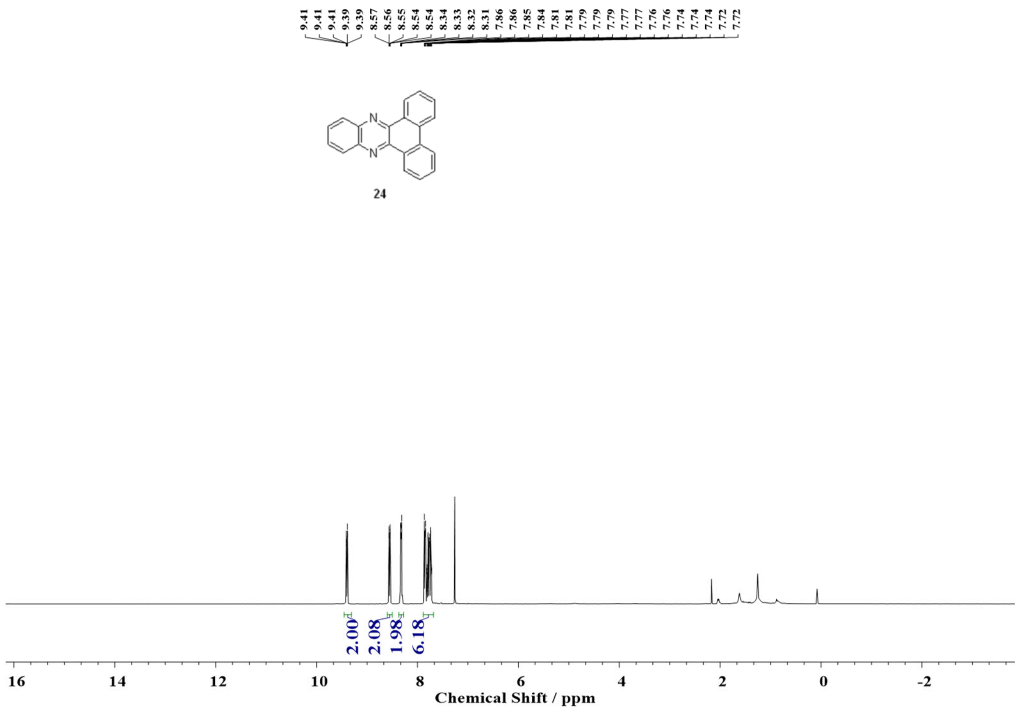

Supplementary Figure 85 ^{1}H NMR (400 MHz, $CDCl_3$) of compound **24**

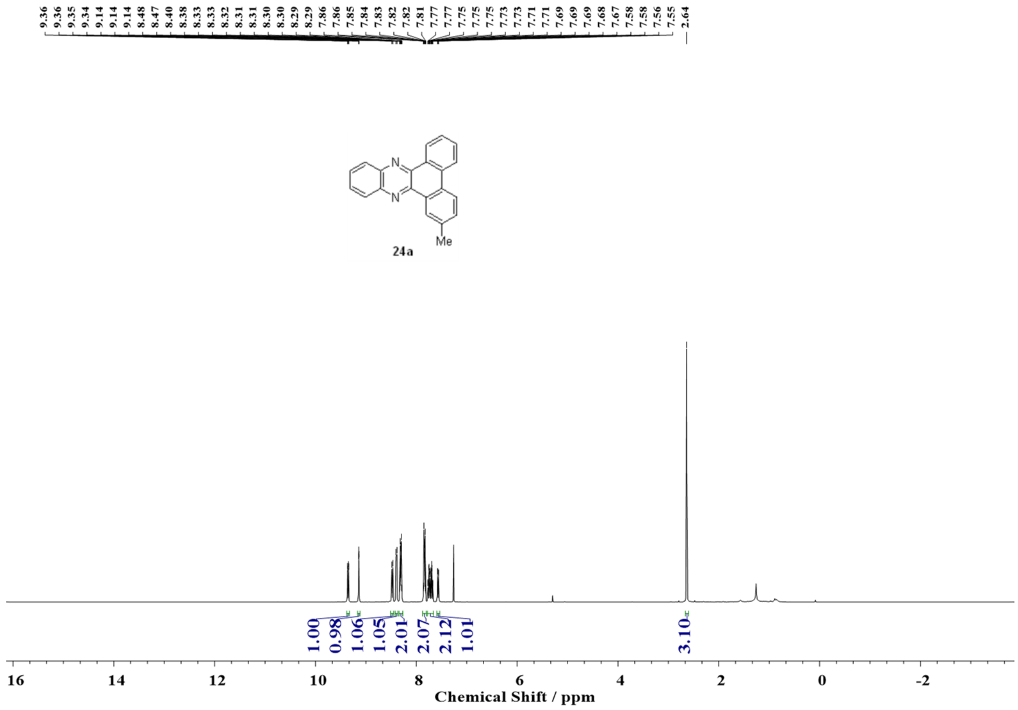

Supplementary Figure 86 ^{1}H NMR (400 MHz, $CDCl_3$) of compound **24a**

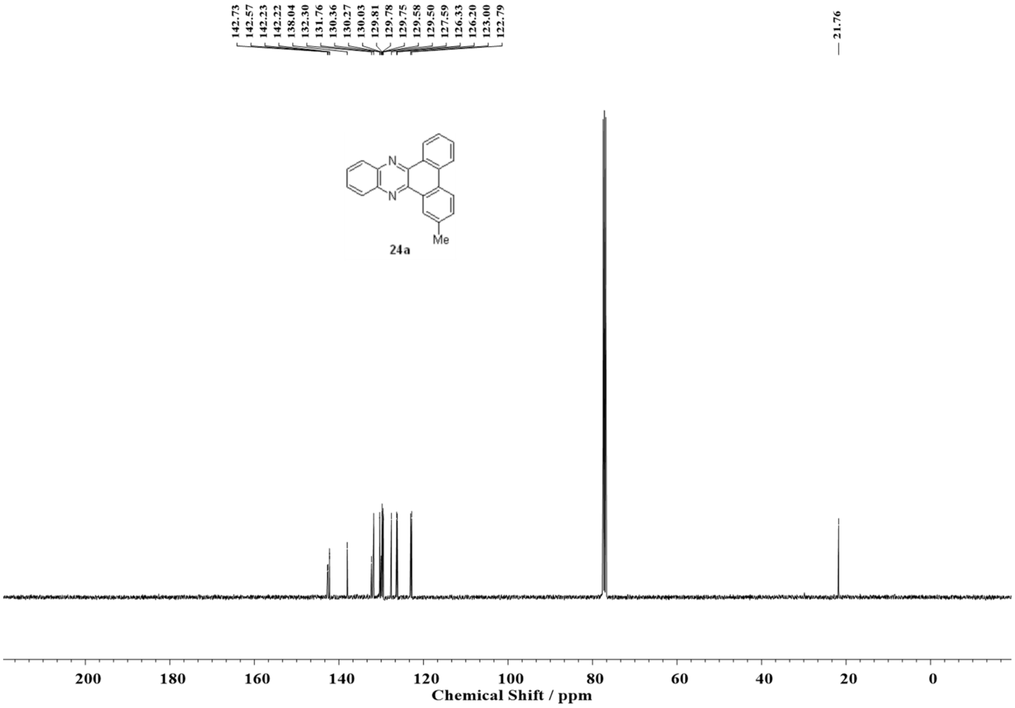

Supplementary Figure 87 ^{13}C NMR (100 MHz, $CDCl_3$) of compound **24a**

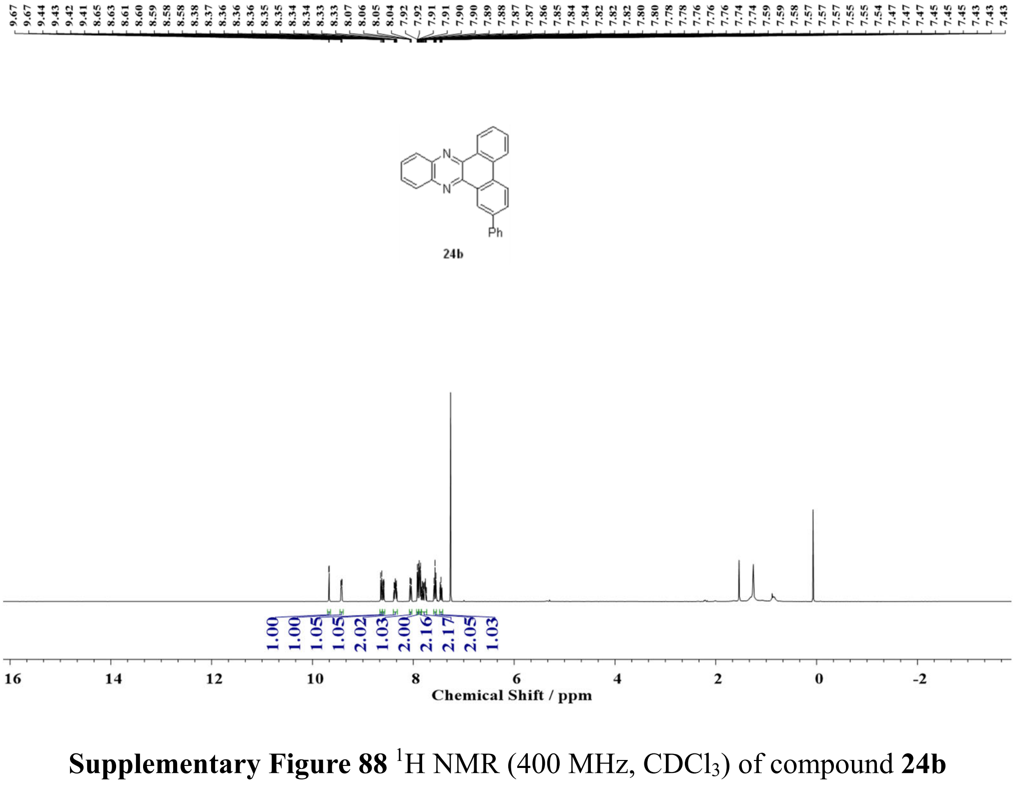

Supplementary Figure 88 ^{1}H NMR (400 MHz, $CDCl_3$) of compound **24b**

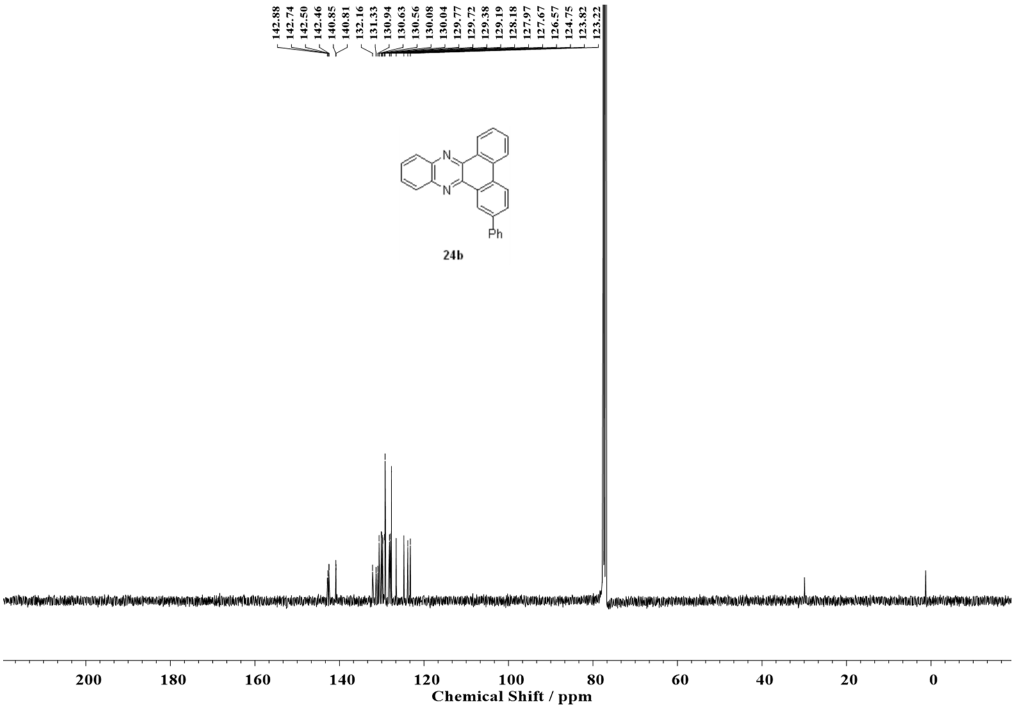

Supplementary Figure 89 ^{13}C NMR (100 MHz, $CDCl_3$) of compound **24b**

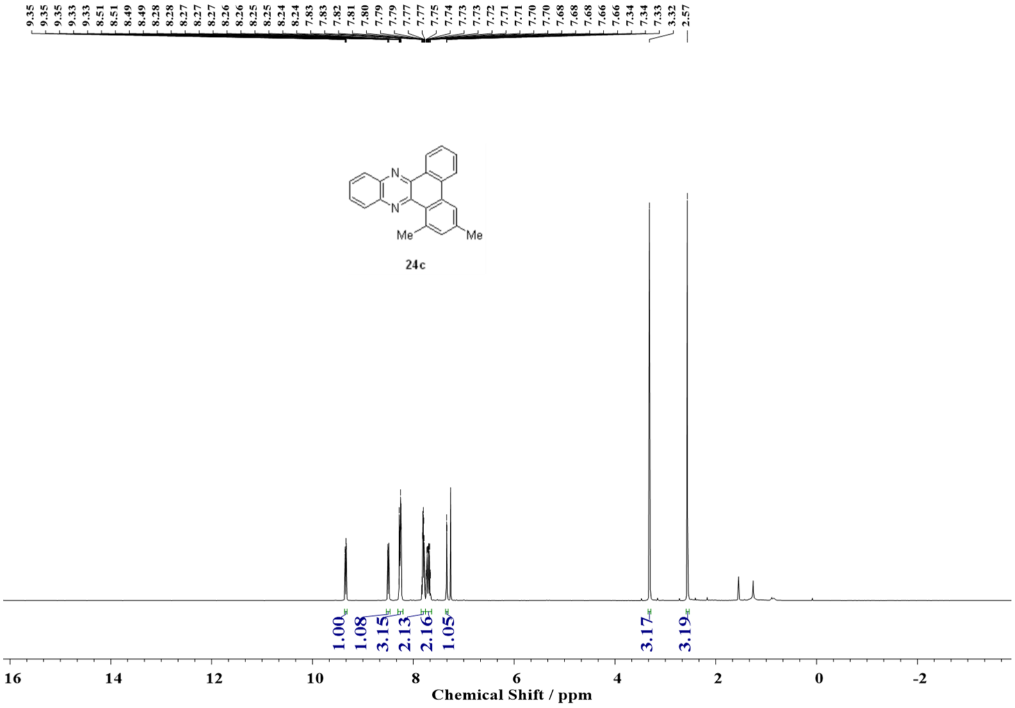

Supplementary Figure 90 ^{1}H NMR (400 MHz, $CDCl_3$) of compound **24c**

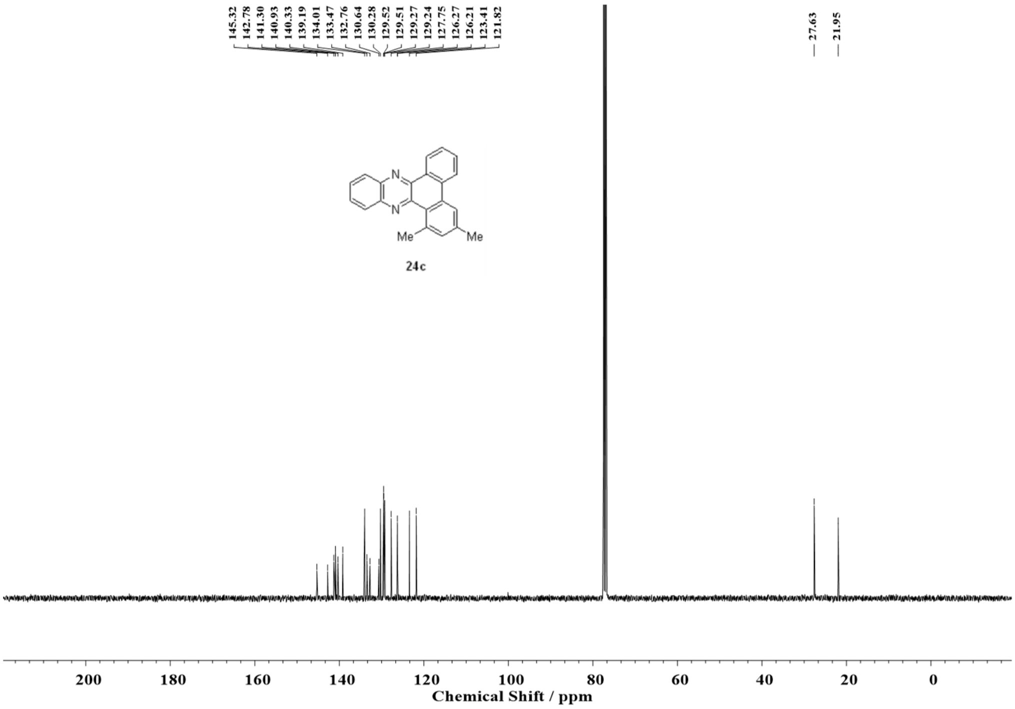

Supplementary Figure 91 ^{13}C NMR (100 MHz, $CDCl_3$) of compound **24c**

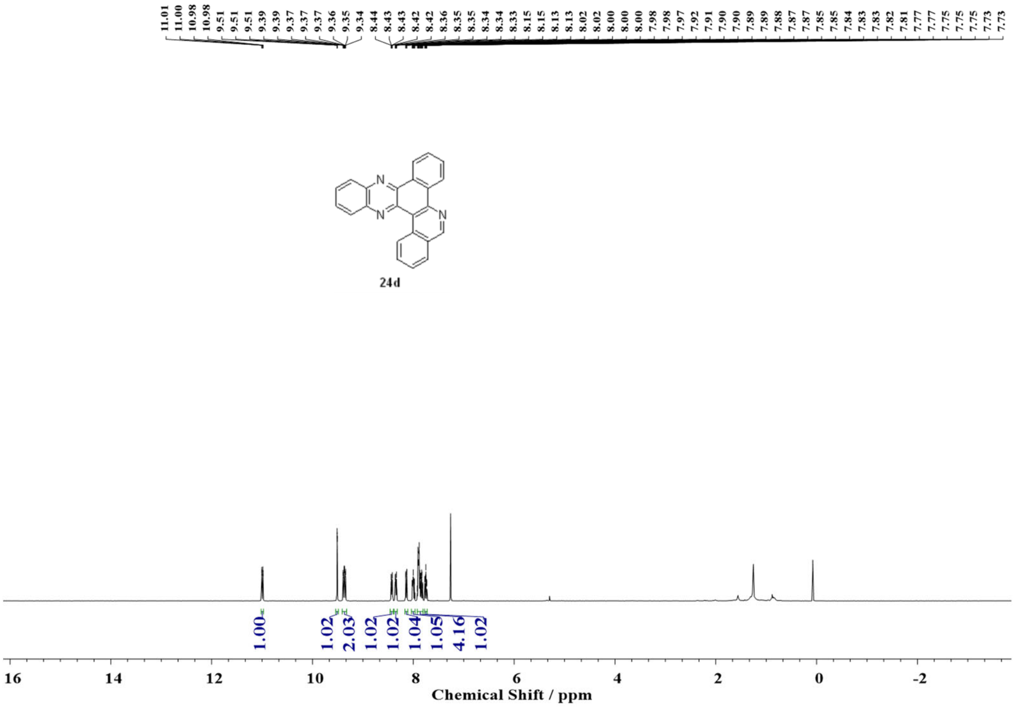

Supplementary Figure 92 ^{1}H NMR (400 MHz, $CDCl_3$) of compound **24d**

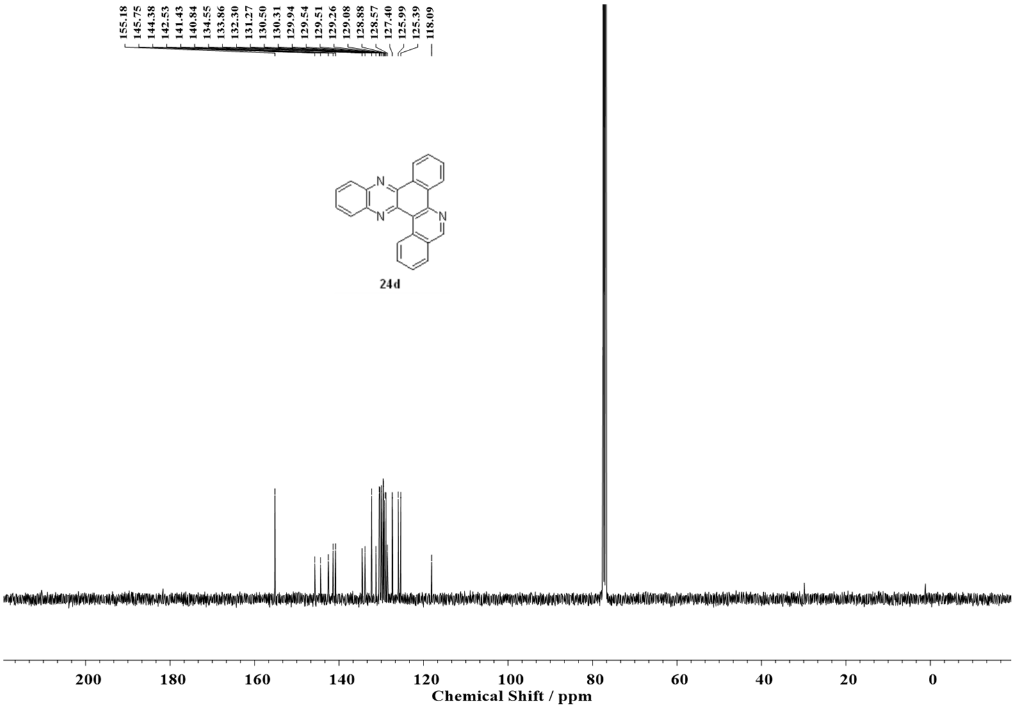

Supplementary Figure 93 ^{13}C NMR (100 MHz, $CDCl_3$) of compound **24d**

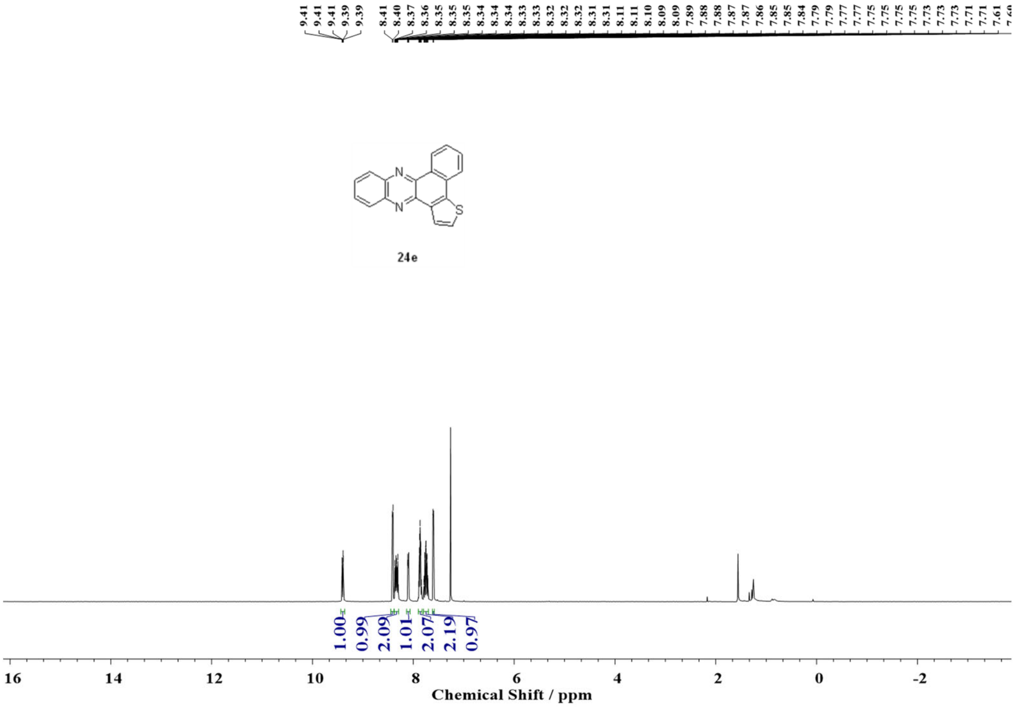

Supplementary Figure 94 ^{1}H NMR (400 MHz, $CDCl_3$) of compound **24e**

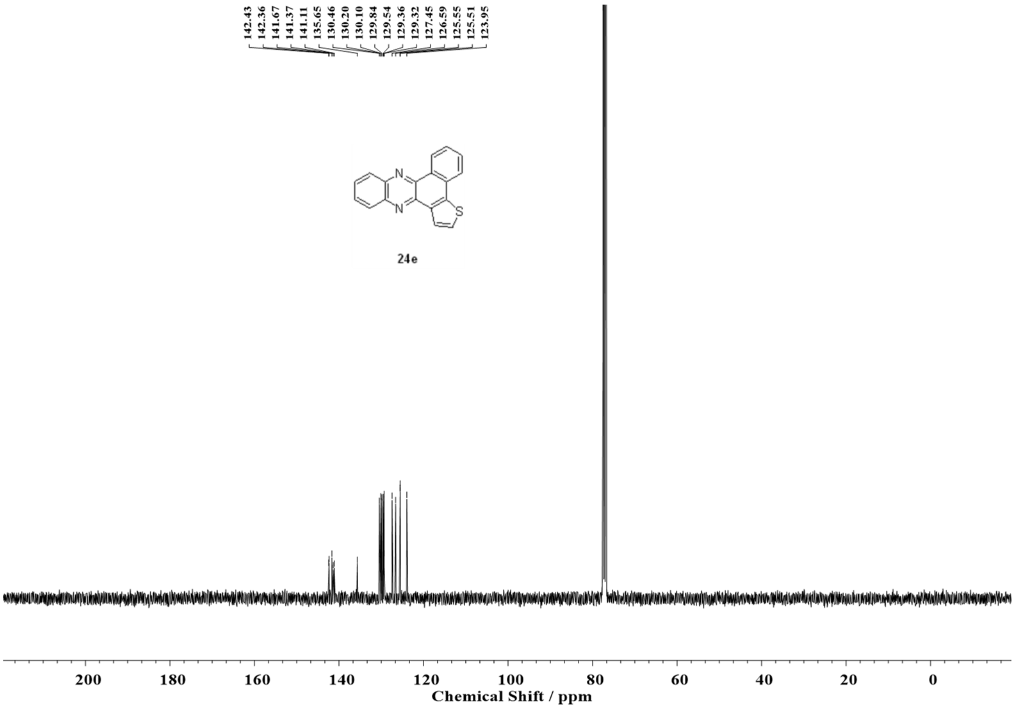

Supplementary Figure 95 ^{13}C NMR (100 MHz, $CDCl_3$) of compound **24e**

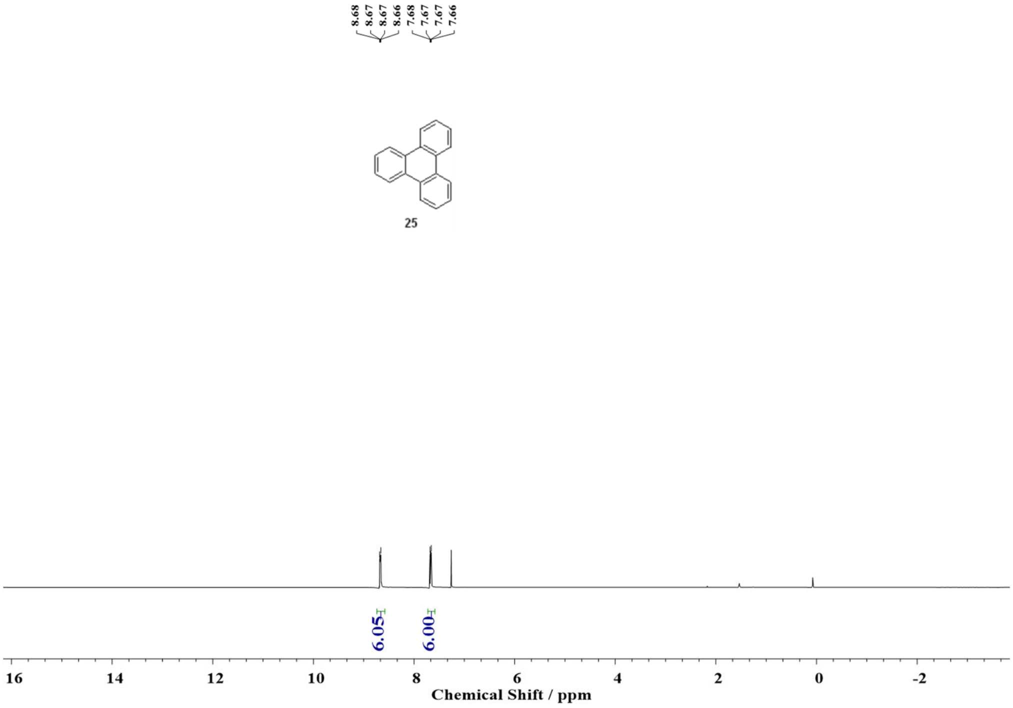

Supplementary Figure 96 ^{1}H NMR (400 MHz, $CDCl_3$) of compound **25**

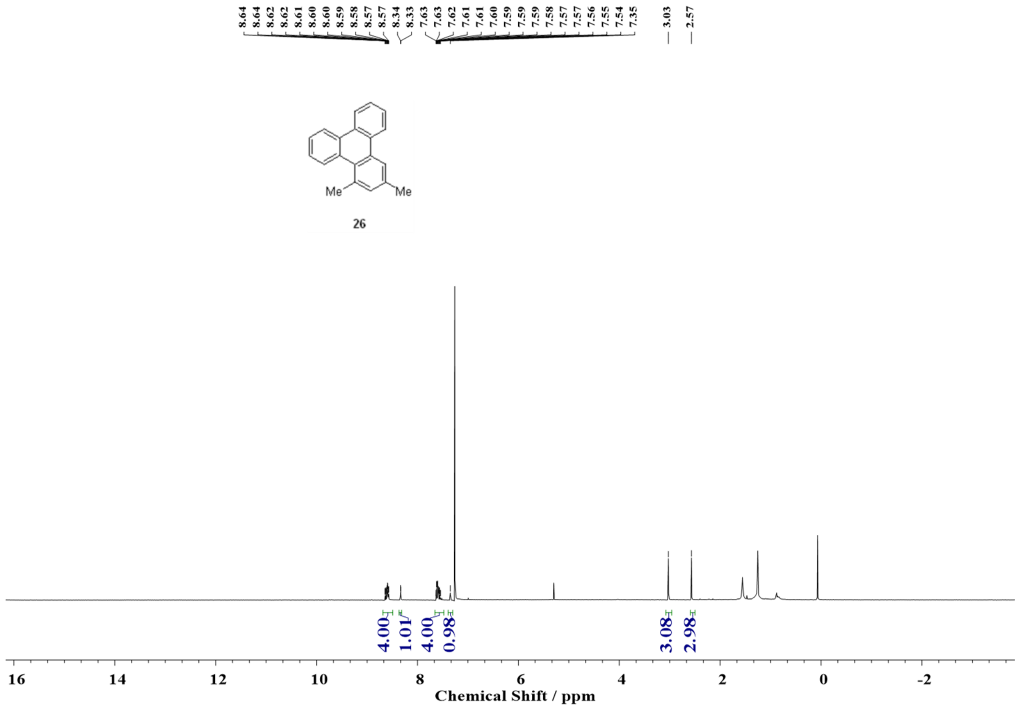

Supplementary Figure 97 ^{1}H NMR (400 MHz, $CDCl_3$) of compound **26**

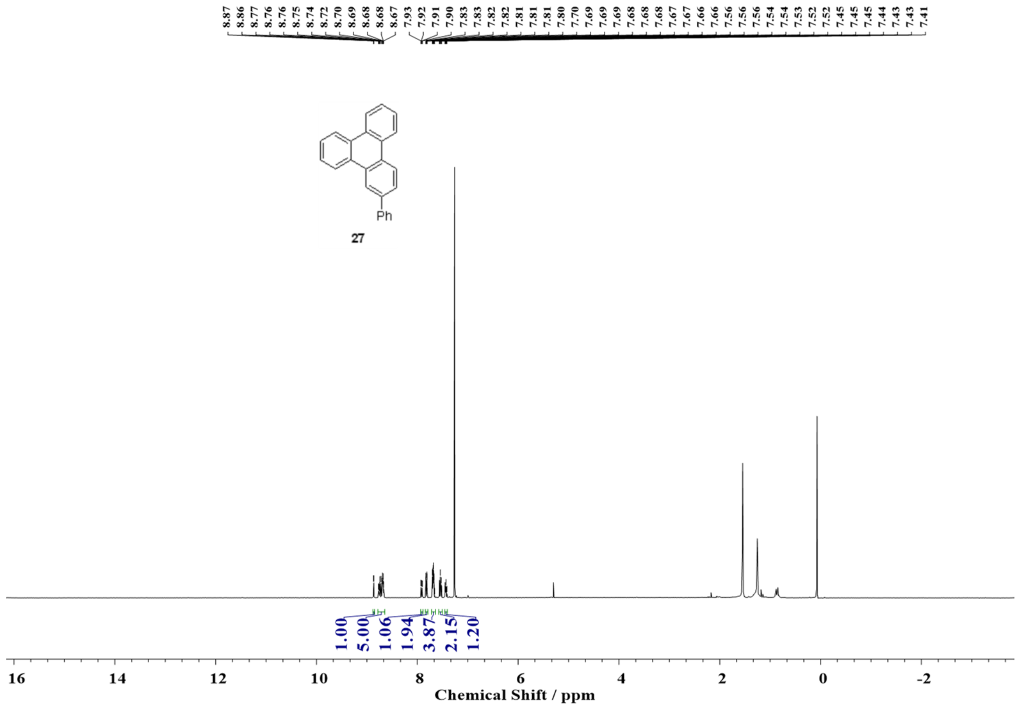

Supplementary Figure 98 ^{1}H NMR (400 MHz, $CDCl_3$) of compound **27**

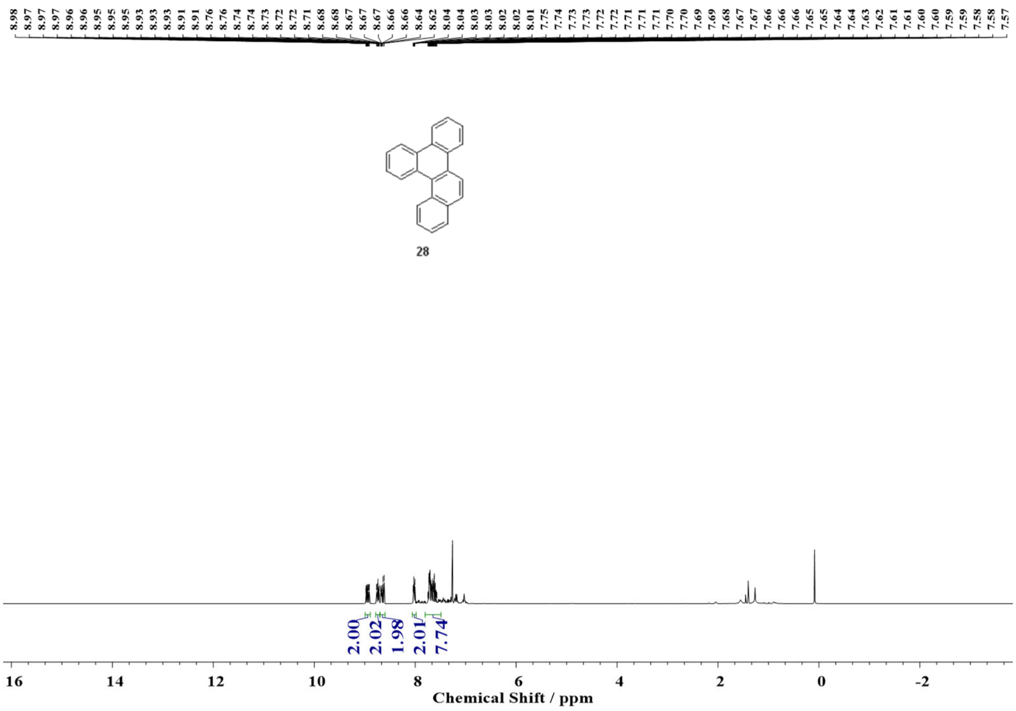

Supplementary Figure 99 ^{1}H NMR (400 MHz, $CDCl_3$) of compound **28**

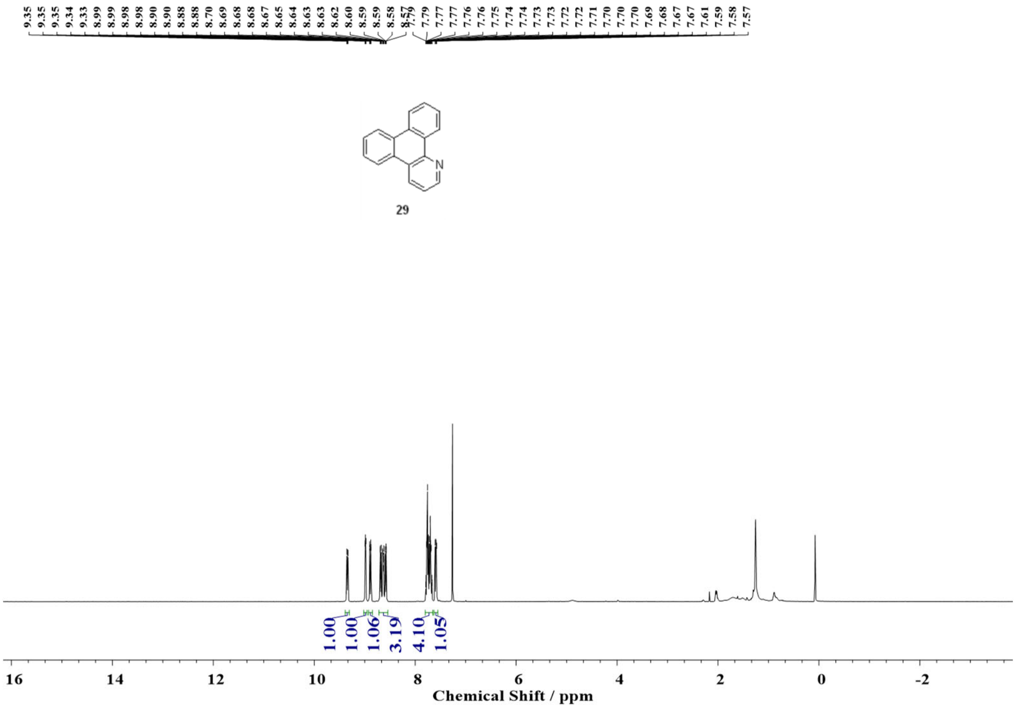

Supplementary Figure 100 ^{1}H NMR (400 MHz, $CDCl_3$) of compound **29**

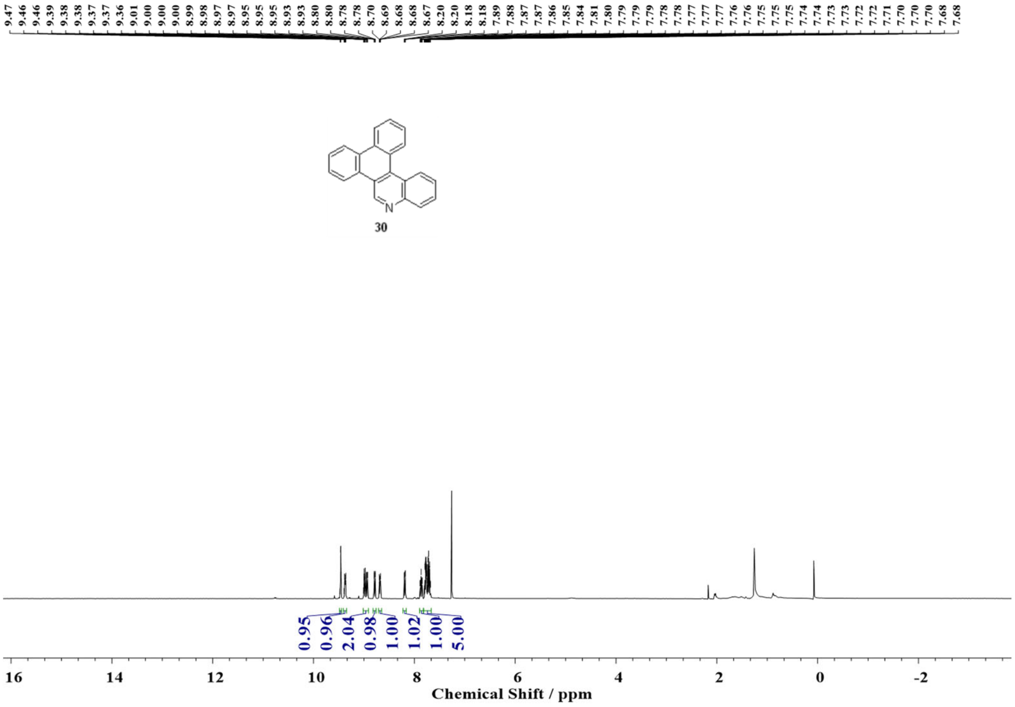

Supplementary Figure 101 ^{1}H NMR (400 MHz, $CDCl_3$) of compound **30**

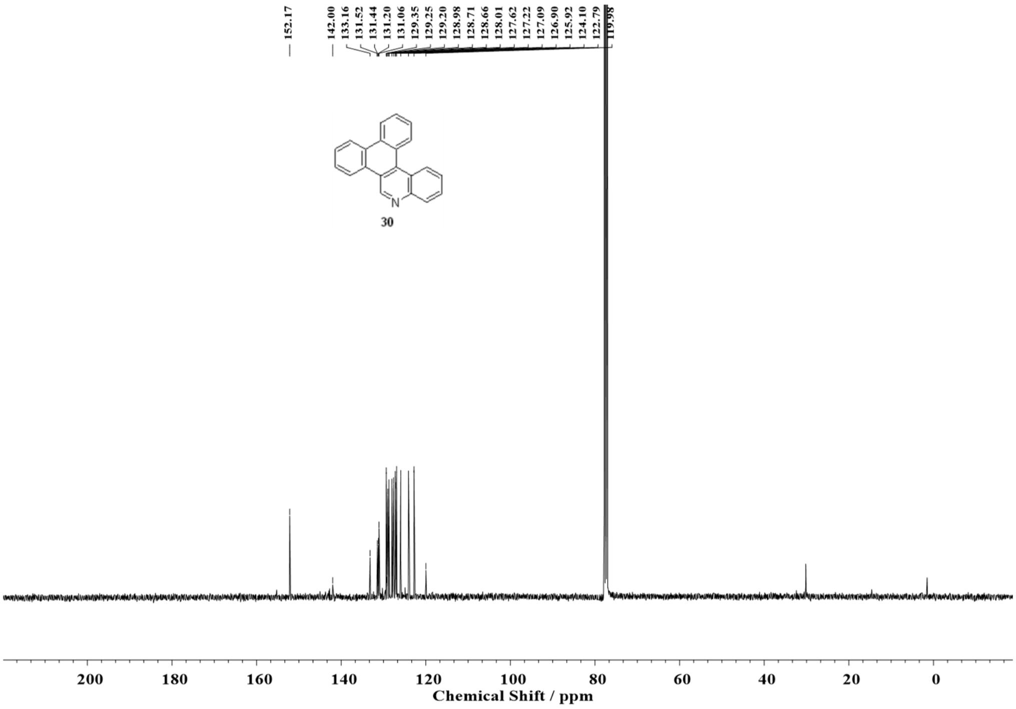

Supplementary Figure 102 ^{13}C NMR (100 MHz, $CDCl_3$) of compound **30**

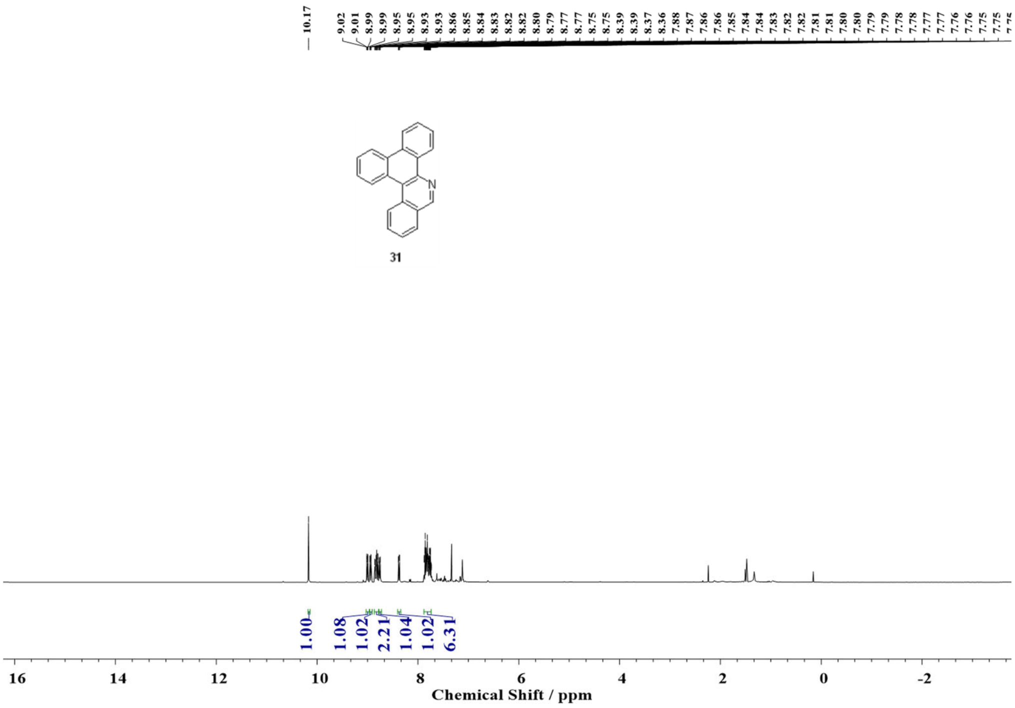

Supplementary Figure 103 ^{1}H NMR (400 MHz, $CDCl_3$) of compound **31**

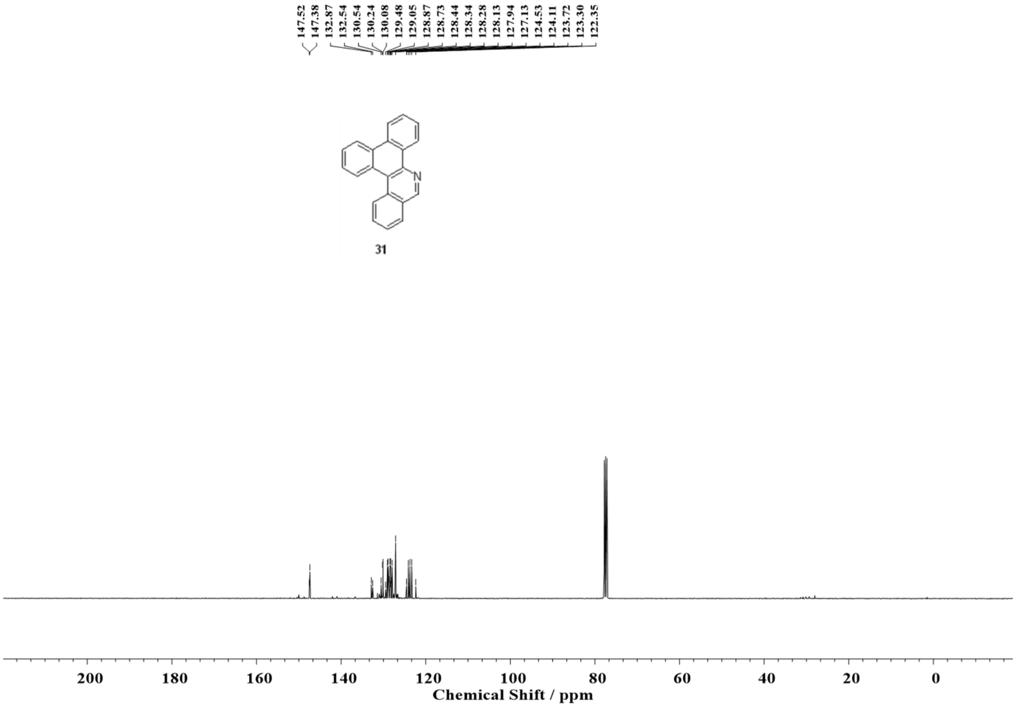

Supplementary Figure 104 ^{13}C NMR (100 MHz, $CDCl_3$) of compound **31**

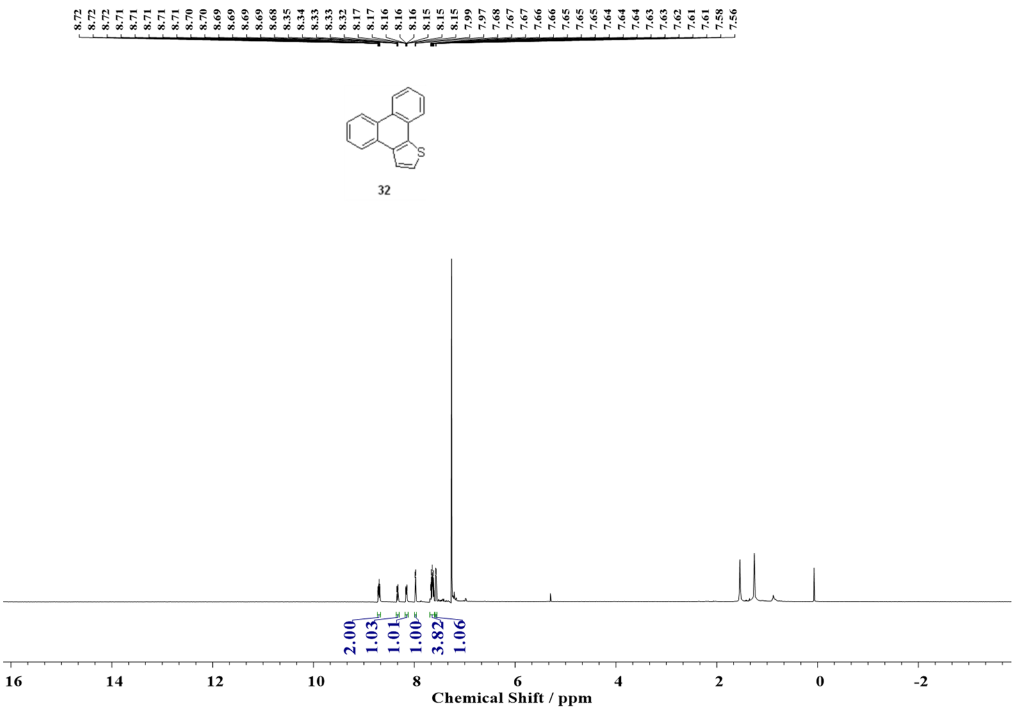

Supplementary Figure 105 ^{1}H NMR (400 MHz, $CDCl_3$) of compound **32**

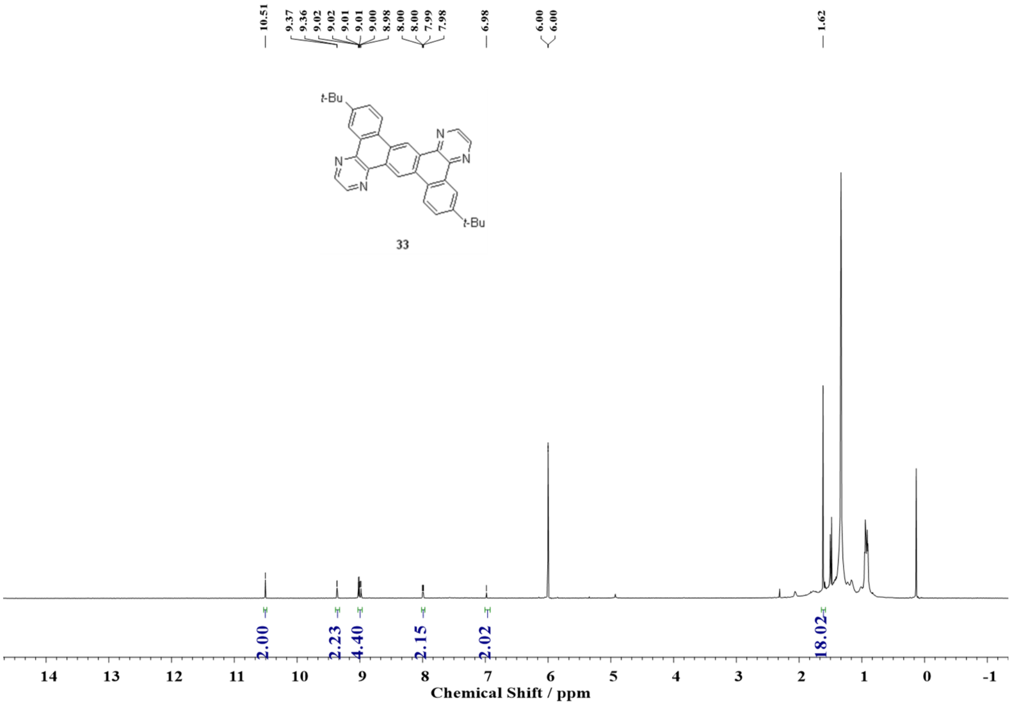

Supplementary Figure 106 ^{1}H NMR (600 MHz, $C_2D_2Cl_4$) of compound **33**

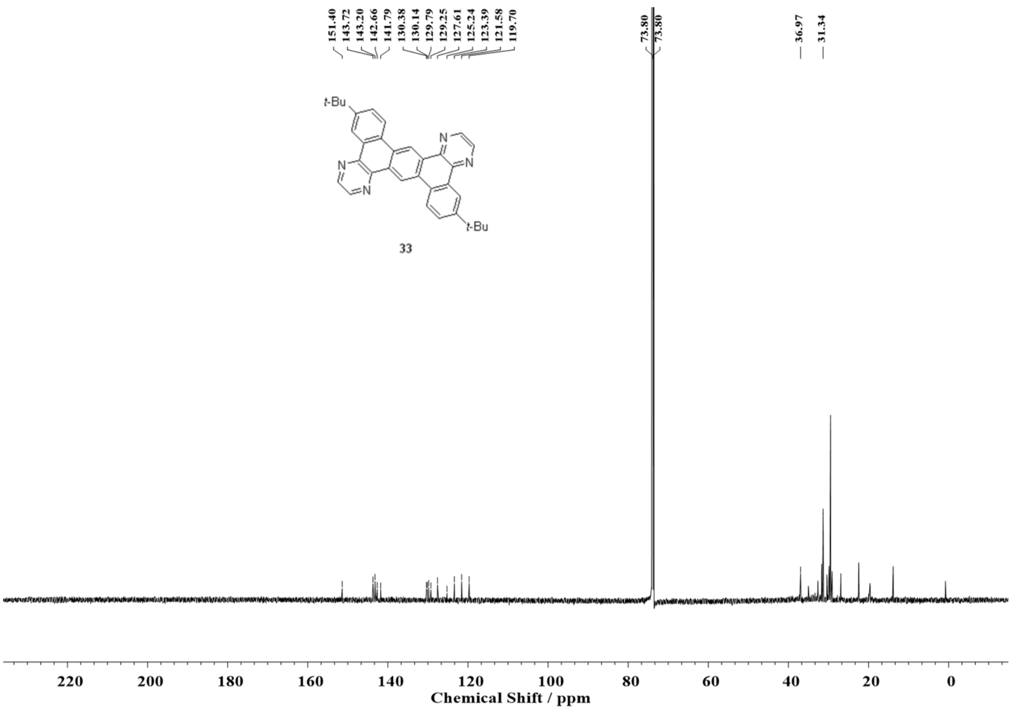

Supplementary Figure 107 ^{13}C NMR (150 MHz, $C_2D_2Cl_4$) of compound **33**

2. Supplementary References

1. Rosenau, C. P., Jelier, B. J., Gossert, A. D. & Togni, A. Exposing the origins of irreproducibility in fluorine NMR spectroscopy. *Angew. Chem. Int. Ed.* **57**, 9528–9533 (2018).
2. Barham, J. P. et al. KO*t*Bu: a privileged reagent for electron transfer reactions? *J. Am. Chem. Soc.* **138**, 7402–7410 (2016).
3. Lee, J. B. et al. Annulative pi-extension of unactivated benzene derivatives through nondirected C–H arylation. *Org. Lett.* **21**, 7004–7008 (2019).
4. Liu, Z. et al. Mild Cu(I)-catalyzed cascade reaction of cyclic diaryliodoniums, sodium azide, and alkynes: efficient synthesis of triazolophenanthridines. *Org. Lett.* **16**, 5600–5603 (2014).
5. Wu, B. & Yoshikai, N. Conversion of 2-iodobiaryls into 2,2'-diiodobiaryls via oxidation-iodination sequences: a versatile route to ladder-type heterofluorenes. *Angew. Chem. Int. Ed.* **54**, 8736–8739 (2015).
6. Letessier, J. & Detert, H. First synthesis of benzopyridoiodolium salts and twofold buchwald-hartwig amination for the total synthesis of quindoline. *Synthesis* **2012**, 290-296 (2011).
7. Zhu, D. et al. Heterocyclic iodoniums for the assembly of oxygen-bridged polycyclic heteroarenes with water as the oxygen source. *Org. Lett.* **20**, 4815–4818 (2018).
8. Wang, M., Chen, S. & Jiang, X. Construction of functionalized annulated sulfone via SO_2/I exchange of cyclic diaryliodonium salts. *Org. Lett.* **19**, 4916–4919 (2017).
9. Lulinski, S. & Serwatowski, J. Bromine as the *ortho*-directing group in the aromatic metalation/silylation of substituted bromobenzenes. *J. Org. Chem.* **68**, 9384-9388 (2003).
10. Ozaki, K., Kawasumi, K., Shibata, M., Ito, H. & Itami, K. One-shot *K*-region-selective annulative π-extension for nanographene synthesis and functionalization. *Nat. Commun.* **6**, 6251 (2015).

11. Mathew, B. P. et al. An annulative synthetic strategy for building triphenylene frameworks by multiple C–H bond activations. *Angew. Chem. Ed. Int.* **56**, 5007–5011 (2017).

12. Yang, S. et al. Site-selective synthesis of functionalized dibenzo[*f*,*h*]quinolines and their derivatives involving cyclic diaryliodonium salts via a decarboxylative annulation strategy. *Chem. Commun.* **54**, 3239–3242 (2018).

13. Sajjadifar, S. et al. Facile method of quinoxaline synthesis using phenol as a new, efficient and cheap catalyst at room temperature. *Am. J. Org. Chem.* **2**, 97–104 (2012).

14. Yang, Y. et al. Palladium-catalyzed synthesis of triphenylenes via sequential C–H activation and decarboxylation. *Org. Lett.* **20**, 5402–5405 (2018).

15. Zhou, S. et al. Organic super-electron-donors: initiators in transition metal-free haloarene–arene coupling. *Chem. Sci.* **5**, 476–482 (2014).

16. Graaf, M. D. & Moeller, K. D. Photoredox catalysts: synthesis of the bipyrazine ligand. *J. Org. Chem.* **80**, 2032–2035 (2015).

17. Zhou, S. et al. Identifying the roles of amino acids, alcohols and 1,2-diamines as mediators in coupling of haloarenes to arenes. *J. Am. Chem. Soc.* **136**, 17818–17826 (2014).

18. Pan, S., Jiang, H., Zhang, Y., Chen, D. & Zhang, Y. Synthesis of triphenylenes starting from 2-iodobiphenyls and iodobenzenes via palladium-catalyzed dual C–H activation and double C–C bond formation. *Org. Lett.* **18**, 5192–5195 (2016).

19. Sobhani, S., Moghadam, H. H., Skibsted, J. & Sansano, J. M. A hydrophilic heterogeneous cobalt catalyst for fluoride-free Hiyama, Suzuki, Heck and Hirao cross-coupling reactions in water. *Green Chem.* **22**, 1353–1365 (2020).

20. Sheldrick, G. M. A short history of SHELX. *Acta Cryst.* **A64**, 112–122 (2008).
